# Comparative accuracy of 1-hour post-load plasma glucose, glycated albumin, and conventional glycemic measures for the diagnosis of type 2 diabetes mellitus: a systematic review and network meta-analysis

**DOI:** 10.3389/fendo.2026.1846553

**Published:** 2026-07-08

**Authors:** Jianzhou Tian, Ping Yuan, Liguo Tan, Youen Zhang, Jianing Wang, Ziheng Cui, Baopeng Tang, Jun Shen

**Affiliations:** 1 Department of Cardiology, Renmin Hospital, Hubei University of Medicine, Shiyan, China; 2 Cardiac Pacing and Electrophysiology Department, The First Affiliated Hospital of Xinjiang Medical University, Urumqi, China

**Keywords:** 1-hour plasma glucose, 2-hour plasma glucose, diagnostic accuracy, fasting plasma glucose, glycated albumin, glycated hemoglobin, network meta-analysis, type 2 diabetes mellitus

## Abstract

**Objective:**

The International Diabetes Federation has proposed 1-hour plasma glucose (1-h PG) as a potential indicator for the diagnosis of type 2 diabetes mellitus (T2DM). This study aimed to systematically compare the diagnostic performance of 1-h PG, glycated albumin (GA), fasting plasma glucose (FPG), glycated hemoglobin (HbA1c), and the combined HbA1c-or-FPG strategy for identifying T2DM using a network meta-analysis of diagnostic test accuracy.

**Methods:**

We systematically searched PubMed, Embase, Web of Science, the Cochrane Library, Scopus, and gray literature for studies evaluating the diagnostic accuracy of 1-h PG, GA, FPG ≥126 mg/dL, HbA1c ≥6.5%, and the combined HbA1c-or-FPG strategy for identifying T2DM, with 2-hour plasma glucose (2-h PG) ≥200 mg/dL during the oral glucose tolerance test (OGTT) as the reference standard. Data extraction and quality assessment were performed independently by two reviewers according to predefined criteria. Statistical analyses were conducted using the Stan package in R and Stata 14.0.

**Results:**

A total of 78 studies involving 183,902 participants were included. The network meta-analysis showed that the pooled sensitivity of 1-h PG, GA, FPG, HbA1c, and the combined HbA1c-or-FPG strategy was 0.87 [95% credible interval (CrI), 0.82–0.91], 0.53 (95% CrI, 0.36–0.71), 0.51 (95% CrI, 0.47–0.55), 0.53 (95% CrI, 0.47–0.58), and 0.64 (95% CrI, 0.54–0.73), respectively. The corresponding pooled specificity values were 0.88 (95% CrI, 0.82–0.92), 0.86 (95% CrI, 0.71–0.95), 0.96 (95% CrI, 0.95–0.97), 0.92 (95% CrI, 0.89–0.94), and 0.89 (95% CrI, 0.81–0.95). The pooled positive likelihood ratios (LR+) were 7.68 (95% CrI, 4.97–11.28) for 1-h PG, 4.39 (95% CrI, 1.86–9.30) for GA, 12.63 (95% CrI, 9.33–16.01) for FPG, 6.75 (95% CrI, 4.81–9.05) for HbA1c, and 6.28 (95% CrI, 3.25–11.26) for the combined strategy. The pooled negative likelihood ratios (LR−) were 0.15 (95% CrI, 0.11–0.20), 0.55 (95% CrI, 0.36–0.74), 0.51 (95% CrI, 0.47–0.55), 0.52 (95% CrI, 0.46–0.57), and 0.41 (95% CrI, 0.31–0.53), respectively. The pooled diagnostic odds ratios (DORs) were 52.44 (95% CrI, 30.17–84.00), 8.53 (95% CrI, 2.70–20.94), 24.94 (95% CrI, 17.90–32.30), 13.15 (95% CrI, 8.94–18.51), and 15.83 (95% CrI, 6.62–31.47), respectively. The areas under the summary receiver operating characteristic curves (SROC-AUCs) were 0.757, 0.714, 0.717, 0.706, and 0.726, respectively. Sensitivity analyses restricted to low-risk-of-bias studies and studies using a 1-h PG threshold ≥11.6 mmol/L, as well as subgroup analyses stratified by general and high-risk populations, yielded results and rankings broadly consistent with the primary analysis. Pairwise meta-analysis findings were also generally consistent with those from the network meta-analysis.

**Conclusions:**

Among the evaluated diagnostic measures, 1-h PG appeared to provide the best overall diagnostic performance for T2DM, with the highest sensitivity, lowest LR−, and highest DOR. It performed better overall than GA, FPG, HbA1c, and the combined HbA1c-or-FPG strategy. By contrast, FPG showed the highest specificity and LR +. The consistency of the findings across sensitivity analyses, subgroup analyses, and pairwise meta-analysis supports the robustness of the main results. However, given the residual heterogeneity across included studies in terms of study design, population characteristics, and diagnostic thresholds, these findings should be interpreted with caution and confirmed by further large-scale, high-quality studies.

**Systematic review registration**: https://www.crd.york.ac.uk/PROSPERO/, identifier CRD420261354518.

## Introduction

The International Diabetes Federation (IDF) estimated that approximately 537 million adults worldwide were living with diabetes in 2021, and this number is projected to increase to 783 million by 2045 (1). Diabetes has become one of the most important global public health challenges, with type 2 diabetes mellitus (T2DM) accounting for the vast majority of cases (2). These figures underscore the importance of early and accurate diagnosis of T2DM for effective disease prevention and control.

At present, the major diagnostic criteria for T2DM include fasting plasma glucose (FPG) ≥126 mg/dL (7.0 mmol/L), 2-hour plasma glucose (2-h PG) ≥200 mg/dL (11.1 mmol/L) during a 75-g oral glucose tolerance test (OGTT), and glycated hemoglobin (HbA1c) ≥6.5% (48 mmol/mol). FPG is simple and convenient to measure, but its ability to detect early dysglycemia is limited. OGTT provides a more comprehensive assessment of glucose metabolism, but it is relatively cumbersome to perform in clinical practice. HbA1c reflects long-term average glycemic exposure, but its measurement may be influenced by erythrocyte lifespan and hemoglobin-related factors (3–5). Moreover, these conventional indicators often identify high-risk individuals only after a certain degree of β-cell dysfunction has already occurred (6). In addition, the combined use of FPG and HbA1c has been suggested to improve the sensitivity of diabetes diagnosis (4).

In recent years, 1-hour plasma glucose (1-h PG) and glycated albumin (GA) have attracted increasing attention. Previous meta-analyses have suggested that, when 2-h PG during OGTT is used as the reference standard for diagnosing T2DM in adults, a 1-h PG threshold of 11.6 mmol/L (209 mg/dL) may represent an optimal diagnostic cutoff (6). In its 2024 position statement, the IDF proposed that 1-h PG ≥11.6 mmol/L (209 mg/dL) during a 75-g OGTT may be used as a diagnostic threshold for T2DM (7). GA reflects average glycemic status over the preceding 2 to 3 weeks and may serve as a useful complement to HbA1c, with potential clinical utility in diabetes diagnosis (8, 9). Existing meta-analyses have also indicated that GA has some value in identifying diabetes (10).

Most previous meta-analyses of diagnostic accuracy have focused on a single test using conventional pairwise approaches. For example, Ahuja et al. conducted a meta-analysis of the diagnostic accuracy of 1-h PG (6), while other studies have evaluated the diagnostic performance of GA (10). More recently, a network meta-analysis compared the accuracy of FPG, HbA1c, and the combined HbA1c-or-FPG strategy for diagnosing diabetes, showing that FPG had superior specificity, whereas the combined HbA1c-or-FPG strategy yielded improved sensitivity (4). However, to date, no network meta-analysis has comprehensively compared 1-h PG, GA, FPG, HbA1c, and the combined HbA1c-or-FPG strategy within a single analytical framework.

Therefore, the present study conducted a network meta-analysis of diagnostic test accuracy to systematically compare the accuracy of these diagnostic approaches using 2-h PG ≥200 mg/dL during OGTT as the common reference standard. Specifically, we compared and ranked their performance in terms of sensitivity, specificity, positive likelihood ratio (LR+), negative likelihood ratio (LR−), diagnostic odds ratio (DOR), and the area under the summary receiver operating characteristic curve (AUC of the SROC curve), with the aim of providing more comprehensive evidence to inform the diagnosis and screening of T2DM.

## Methods

### Study protocol and registration

This study was reported in accordance with the Preferred Reporting Items for a Systematic Review and Meta-analysis of Diagnostic Test Accuracy Studies (PRISMA-DTA) and the PRISMA extension statement for Network Meta-Analyses (PRISMA-NMA) (11, 12). The study protocol was registered in PROSPERO under registration number CRD420261354518.

### Literature search strategy

A systematic search was conducted in PubMed, Embase, Web of Science, the Cochrane Library, and Scopus, with additional searches of gray literature, to identify studies evaluating the diagnostic performance of 1-h PG, GA, FPG ≥126 mg/dL, HbA1c ≥6.5%, and the combined HbA1c-or-FPG strategy for identifying T2DM, using 2-h PG ≥200 mg/dL during OGTT as the reference standard. The search was limited to English-language studies published from database inception to July 2, 2025. The search strategy combined controlled vocabulary and free-text terms, including but not limited to diabetes mellitus, 1-hour plasma glucose, 1-h PG, glycated albumin, HbA1c, fasting plasma glucose, FPG, oral glucose tolerance test, OGTT, sensitivity, and specificity. In addition, the reference lists of included studies were manually screened to identify potentially eligible studies. The full search strategy is provided in **Supplementary File S1**.

### Eligibility criteria

Studies were included if they met all of the following criteria: (1) participants were adults aged ≥18 years from either general or high-risk populations, with no prior diagnosis of diabetes at enrollment; (2) the study had a cross-sectional or cohort design and evaluated at least one of the following diagnostic or screening strategies for T2DM: 1-h PG, GA, FPG ≥126 mg/dL, HbA1c ≥6.5%, or the combined HbA1c-or-FPG strategy; (3) 2-h PG during a 75-g OGTT was used as the common reference standard, with T2DM defined as 2-h PG ≥200 mg/dL (11.1 mmol/L); and (4) sufficient data were available to extract or reconstruct the numbers of true positives (TP), false positives (FP), false negatives (FN), and true negatives (TN), or to calculate diagnostic accuracy measures such as sensitivity and specificity. The combined HbA1c-or-FPG strategy was defined as test-positive when either HbA1c ≥6.5% or FPG ≥126 mg/dL was present.

### Exclusion criteria

Studies were excluded if any of the following criteria were met: 1) the study population included patients with type 1 diabetes, gestational diabetes mellitus, cystic fibrosis-related diabetes, or individuals with previously diagnosed T2DM who were already receiving glucose-lowering therapy; 2) a composite reference standard was used in which the diagnostic definition after a 75-g OGTT incorporated FPG, HbA1c, or other index tests in addition to 2-h PG; or 3) the study was a case-control study, meta-analysis, case report, conference abstract, or duplicate publication. For duplicate reports, only the study with the largest sample size was included.

### Data extraction and quality assessment

Data were independently extracted by two reviewers and cross-checked for accuracy. Any discrepancies were resolved through discussion with a third reviewer until consensus was reached. The following information was extracted: first author, publication year, country, study design, study setting, sample size, participant age, target population, index test(s) and corresponding diagnostic threshold(s), reference standard, and the numbers of TP, FP, FN, and TN. Methodological quality was assessed using the Quality Assessment of Diagnostic Accuracy Studies-2 (QUADAS-2) tool, which evaluates risk of bias across four domains: patient selection, index test, reference standard, and flow and timing. Applicability concerns were assessed in the domains of patient selection, index test, and reference standard (13, 14).

### Statistical analysis

#### Network meta-analysis

A network plot was generated using R 4.5.1. Within a Bayesian framework, the network meta-analysis was performed using an analysis-of-variance (ANOVA) model implemented in the rstan package. A random-effects model was used to compare the diagnostic strategies both directly and indirectly in terms of sensitivity, specificity, LR+, LR−, and DOR. The model parameters were specified as follows: four chains, 10,000 iterations, 1,000 warm-up iterations, and a thinning interval of 5. Results were reported as pooled estimates with 95% credible intervals (CrIs). Forest plots and league tables were generated for the network meta-analysis. To rank the diagnostic strategies, the surface under the cumulative ranking curve (SUCRA) was calculated, with a higher SUCRA value indicating a better relative ranking. Consistency between direct and indirect evidence was assessed using the node-splitting method, with P > 0.05 indicating no statistically significant evidence of inconsistency. SROC curves were also plotted to evaluate the overall diagnostic performance of each strategy (15, 16).

Pairwise meta-analysis

A conventional pairwise meta-analysis was conducted using the midas command in Stata 14.0 under a random-effects model. Pooled estimates of sensitivity, specificity, LR+, LR−, and DOR were calculated and presented with 95% CIs (17).

Sensitivity and subgroup analyses

Sensitivity and subgroup analyses: For the sensitivity analysis, the network meta-analysis was repeated after restricting the dataset to studies judged to be at low risk of bias. Subgroup analyses were performed by restricting studies to those with 1-h PG thresholds ≥11.6 mmol/L, those conducted in general populations, and those conducted in high-risk populations.

## Results

### Literature search results

**Figure 1:**
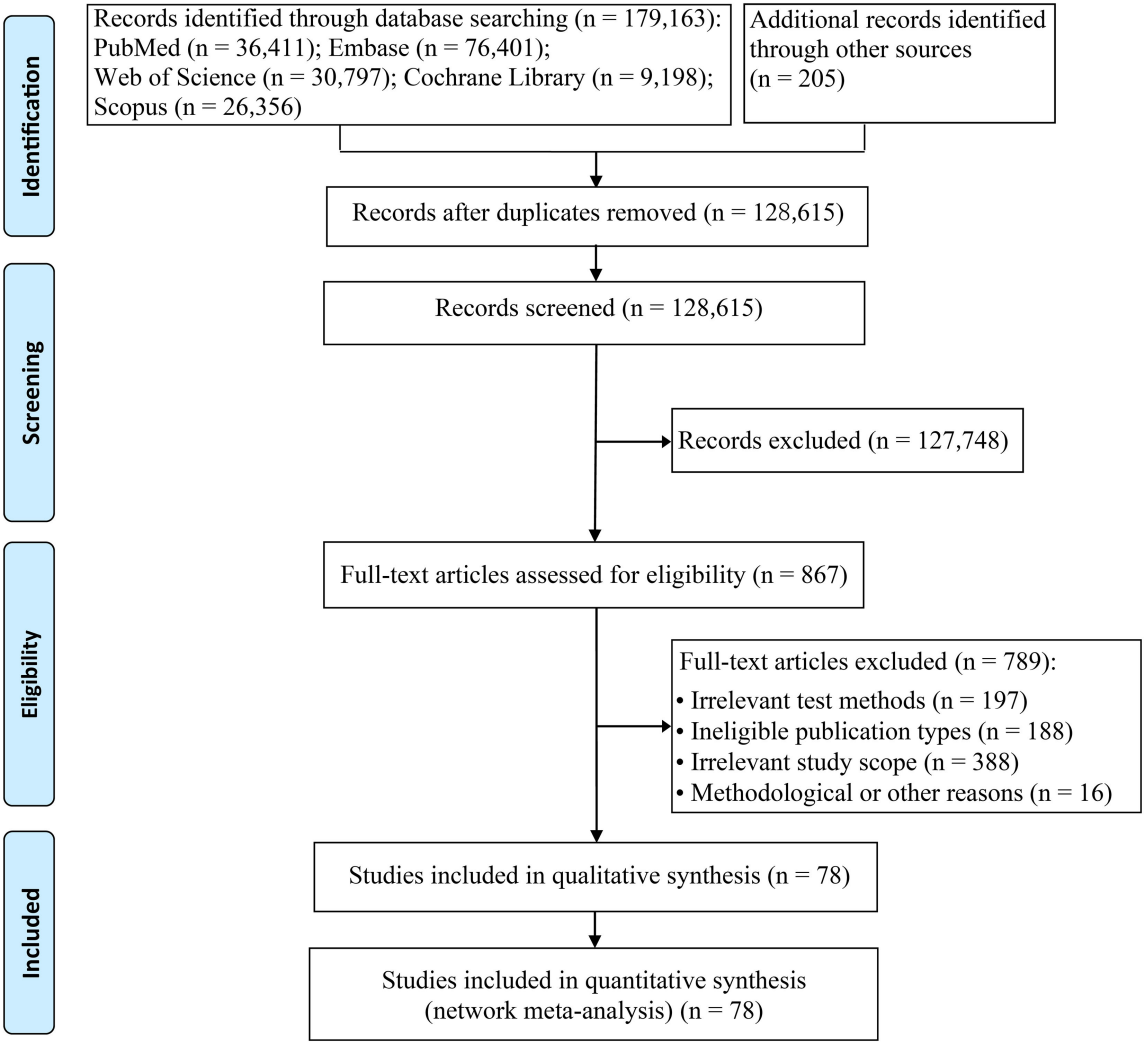
PRISMA flow diagram of the study selection process.

A total of 179,368 records were identified through the database and gray-literature searches. After deduplication and stepwise screening, 78 studies met the eligibility criteria for inclusion. The study selection process is shown in **Figure 1**, and the citation details of the included studies are provided in **Supplementary File S2**.

### Study characteristics

A total of 78 studies involving 183,902 participants were included, comprising 72 cross-sectional studies and 6 cohort studies. Of these, 16 studies evaluated 1-h PG, 4 evaluated GA, 47 evaluated FPG, 30 evaluated HbA1c, and 7 evaluated the combined HbA1c-or-FPG strategy for diagnosing T2DM. Participants were recruited from clinical settings in 37 studies and from community settings in 41 studies. In addition, 49 studies enrolled general populations, whereas 29 studies enrolled high-risk populations. The main characteristics of the included studies are summarized in **Table 1**.

**Table 1:** Summary of the main characteristics of the included studies.

Author	Year	Country	Study setting	Study design	Type of population	Sample (N)	Age at baseline (years)	Test	Reference test
Tripathy	1990	Finland	Clinic	Cross-sectional	General population	2995	46.2 ± 13.7	1-h PG	OGTT
Succurro	2005	Italy	Clinic	Cross-sectional	High-risk population	3324	48.4 ± 13.9	1-h PG	OGTT
Abdul-Ghani	1996	Germany	Community	Cross-sectional	General population	2679	52.6 ± 16.5	1-h PG	OGTT
La Sala	2014	Italy	Clinic	Cross-sectional	High-risk population	531	59.4 ± 9.9	1-h PG	OGTT
Bianchi	2003	Italy	Clinic	Cross-sectional	High-risk population	916	49.3 ± 11.3	1-h PG	OGTT
Bergman	1979	Israel	Community	Cross-sectional	General population	2092	51.3 ± 8.0	1-h PG	OGTT
Pyörälä	1966	Finland	Community	Cross-sectional	General population	1026	44.0 ± 7.7	1-h PG	OGTT
Sai Prasanna	1991	India	Clinic	Cross-sectional	High-risk population	9651	45.0 ± 12.0	1-h PG	OGTT
Mutt	2001	Finland	Community	Cross-sectional	General population	933	56.8 ± 0.6	1-h PG	OGTT
Paddock	1966	USA	Community	Cohort	High-risk population	2664	32.2 ± 15.1	1-h PG	OGTT
Oka	2006	Japan	Community	Cross-sectional	General population	2085	52.6 ± 7.2	1-h PG	OGTT
Abdul-Ghani	1992	USA	Community	Cross-sectional	General population	689	49.8 ± 12.1	1-h PG	OGTT
Kim	2017	Korea	Community	Cohort	General population	8518	51.6 ± 8.0	1-h PG, HbA1c, FPG, HbA1c or FPG	OGTT
Castro	2025	Brazil	Clinic	Cross-sectional	High-risk population	1797	49.6 ± 15.0	1-h PG	OGTT
Ferrannini	2022	27countries	Clinic	Cross-sectional	High-risk population	918	62.8 ± 10.1	1-h PG	OGTT
Thenmozhi	2025	Indian	Clinic	Cross-sectional	High-risk population	113	48.0 ± 12.6	1-h PG	OGTT
Wiener	1998	UK	Clinic	Cross-sectional	General population	401	52.0	HbA1c, FPG	OGTT
Tanaka	2001	Japan	Clinic	Cross-sectional	High-risk population	866	56 ± 0.4	HbA1c, FPG, HbA1c or FPG	OGTT
Adamu	2011	Nigeria	Clinic	Cross-sectional	High-risk population	31	54.0 ± 6.3	HbA1c, FPG	OGTT
Valentine	2011	Australia	Clinic	Cross-sectional	General population	43	63.8 ± 19.6	HbA1c	OGTT
Zemlin	2011	South Africa	Community	Cross-sectional	General population	819	43.0 – 68.0	HbA1c	OGTT
Homko	2012	USA	Community	Cross-sectional	High-risk population	195	53.0 ± 10.4	HbA1c, FPG	OGTT
Marini	2012	Italy	Community	Cross-sectional	High-risk population	1019	44.0	HbA1c	OGTT
Adamska	2012	Poland	Clinic	Cross-sectional	General population	441	18.0 -79.0	HbA1c	OGTT
Lee	2013	Korea	Clinic	Cross-sectional	General population	4616	50.0 ± 13.0	HbA1c, FPG, HbA1c or FPG	OGTT
Alqahtani	2013	Saudi Arabia	Clinic	Cross-sectional	General population	1814	54.3 ± 13.6	HbA1c	OGTT
Franco	2014	Brazil	Community	Cross-sectional	General population	548	≥ 20.0	HbA1c	OGTT
Karnchanasorn	2016	USA	Community	Cross-sectional	General population	5764	46.0 ± 19.0	HbA1c	OGTT
Aviles-Santa	2016	USA	Community	Cross-sectional	General population	15507	18.0 -74.0	HbA1c, FPG, HbA1c or FPG	OGTT
Camacho	2016	USA	Community	Cross-sectional	High-risk population	175	40.0 ± 14.0	HbA1c	OGTT
Herath	2017	Sri Lanka	Community	Cross-sectional	General population	254	50.5 ± 12.0	HbA1c, FPG, HbA1c or FPG	OGTT
Joung	2018	Korea	Clinic	Cross-sectional	High-risk population	515	47.2 ± 11.9	HbA1c, FPG, HbA1c or FPG	OGTT
Lopez	2018	Colombia	Community	Cross-sectional	General population	1113	53.8 ± 15.2	HbA1c, FPG, HbA1c or FPG	OGTT
Prakaschandra	2018	South Africa	Clinic	Cross-sectional	General population	1076	40.0 ± 14.0	HbA1c, FPG	OGTT
Aamir	2019	Pakistan	Community	Cross-sectional	General population	1029	45.2 ± 14.0	HbA1c	OGTT
Thewjitcharoen	2019	Thailand	Clinic	Cross-sectional	High-risk population	512	50.3 ± 12.7	HbA1c	OGTT
Basit	2020	Pakistan	Community	Cohort	General population	6836	31.0 – 50.0	HbA1c	OGTT
Cetin	2020	Turkey	Clinic	Cross-sectional	General population	201	49.3 ± 10.4	HbA1c, FPG	OGTT
Tucker	2020	USA	Community	Cross-sectional	General population	7412	20.0 – 80.0	HbA1c, FPG	OGTT
Araneta	2010	Japan	Community	Cross-sectional	General population	933	54.2	HbA1c	OGTT
Hird	2016	South Africa	Community	Cross-sectional	General population	1077	39.7	HbA1c	OGTT
Huang	2013	China	Community	Cross-sectional	General population	6540	52.0	HbA1c	OGTT
Kramer	2010	Brazil	Community	Cross-sectional	General population	2107	69.4 ± 11.1	HbA1c	OGTT
Lim	2018	Singapore	Community	Cross-sectional	General population	3540	42.5 ± 14.5	HbA1c	OGTT
van’t Riet	2010	Netherlands	Community	Cross-sectional	General population	2753	53.5 ± 6.7	HbA1c	OGTT
Lee	1997	Singapore	Clinic	Cross-sectional	General population	865	18.0 – 67.0	FPG	OGTT
Ko	1998	Hong Kong	Clinic	Cross-sectional	High-risk population	2877	36.6 ± 0.2	FPG	OGTT
Nitiyanant	1998	Thailand	Clinic	Cross-sectional	High-risk population	496	45.0 ± 12.2	FPG	OGTT
Chang	1998	China	Community	Cross-sectional	General population	5303	50.3 ± 12.6	FPG	OGTT
Croxson	1998	UK	Clinic	Cross-sectional	General population	599	47.0	FPG	OGTT
Puavilai	1999	Thailand	Clinic	Cross-sectional	High-risk population	1051	50.0 ± 12.6	FPG	OGTT
Shaw	1999	Mauritius	Community	Cohort	General population	3527	25.0 – 74.0	FPG	OGTT
Lujan	2000	Spain	Clinic	Cross-sectional	High-risk population	580	58.1 ± 10.7	FPG	OGTT
Tai	2000	Singapore	Community	Cross-sectional	General population	3407	18.0 – 69.0	FPG	OGTT
Hwu	2001	China	Clinic	Cross-sectional	High-risk population	247	41.0	FPG	OGTT
Moran	2001	Mexico	Community	Cross-sectional	General population	712	45.2 ± 7.0	FPG	OGTT
Perry	2001	USA	Community	Cohort	High-risk population	244	53.6 ± 11.4	FPG	OGTT
Gatling	2001	UK	Clinic	Cohort	High-risk population	1868	56.1 ± 18.2	FPG	OGTT
Drzewoski	2001	Poland	Clinic	Cross-sectional	High-risk population	1360	65.5 ± 6.9	FPG	OGTT
Nakagami	2002	Multiple countries	Clinic	Cross-sectional	General population	17512	30.0 – 89.0	FPG	OGTT
Mannucci	2003	Italy	Community	Cross-sectional	General population	333	30.0 – 70.0	FPG	OGTT
Daniel	2006	Australia	Community	Cross-sectional	General population	3249	34.9 ± 15.7	FPG	OGTT
Shrestha	2006	Nepal	Community	Cross-sectional	General population	924	≥ 40.0	FPG	OGTT
Soma	2006	South Africa	Clinic	Cross-sectional	High-risk population	120	58.0	FPG	OGTT
Al-Lawati	2007	Oman	Community	Cross-sectional	General population	4917	≥ 20.0	FPG	OGTT
Gao	2008	China	Community	Cross-sectional	General population	1856	20.0 – 74.0	FPG	OGTT
Koike	2009	Japan	Clinic	Cross-sectional	General population	143	20.0 – 39.0	FPG	OGTT
Hofsø	2010	Norway	Clinic	Cross-sectional	High-risk population	670	31.0 – 50.0	FPG	OGTT
Wen	2012	China	Clinic	Cross-sectional	High-risk population	994	20.0 – 80.0	FPG	OGTT
Huang	2015	USA	Community	Cross-sectional	General population	5782	46.0 ± 19.0	FPG	OGTT
Aekplakorn	2015	Thailand	Clinic	Cross-sectional	High-risk population	6884	35.0 – 65.0	FPG	OGTT
Kim	2016	Korea	Clinic	Cross-sectional	High-risk population	236	54.5 ± 10.9	FPG	OGTT
Kengne	2017	South Africa	Clinic	Cross-sectional	General population	793	50.5 ± 15.0	FPG	OGTT
Katulanda	2019	Sri Lanka	Community	Cross-sectional	General population	4014	45.3 ± 15.0	FPG	OGTT
Chume	2019	Brazil	Clinic	Cross-sectional	High-risk population	242	54.4 ± 13.0	GA	OGTT
Zemlin	2019	South Africa	Community	Cross-sectional	General population	1294	47.8	GA	OGTT
Wu	2016	China	Community	Cohort	General population	1559	50.4 ± 12.6	GA	OGTT
Ikezaki	2015	Japan	Community	Cross-sectional	General population	176	59.0	GA	OGTT

1-h PG, 1-hour plasma glucose; OGTT, oral glucose tolerance test; HbA1c, glycated hemoglobin; FPG, fasting plasma glucose; GA, glycated albumin.

### Risk-of-bias assessment

According to QUADAS-2, 39 studies were judged to be at low risk of bias across all domains, 41 studies were rated as having low risk in all bias domains, and 69 studies were considered to have low applicability concerns across all relevant domains. Detailed results of the risk-of-bias assessment are shown in **Supplementary Table 1**.

### Network meta-analysis

#### Network plot

**Figure 2:**
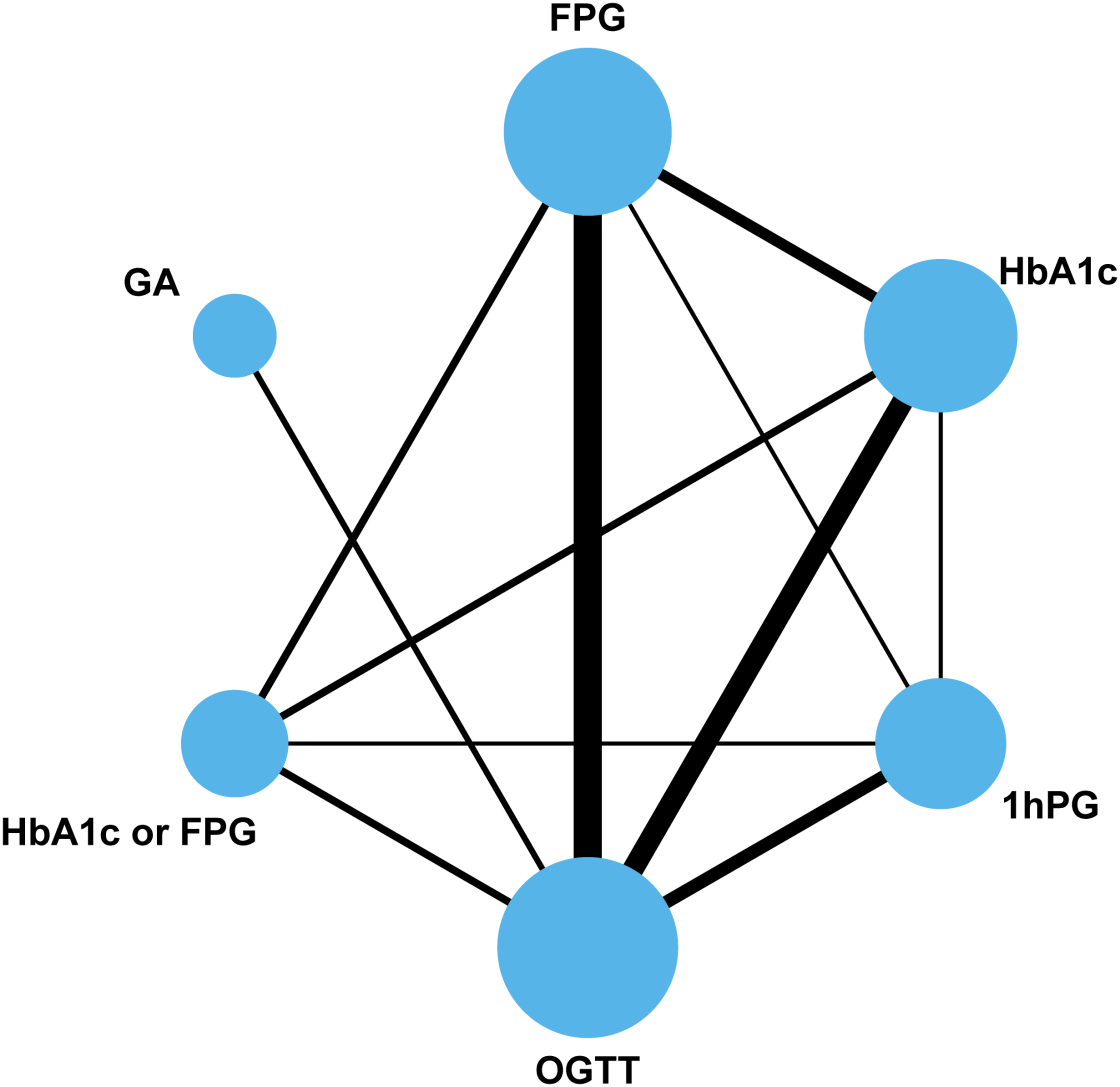
Network plot of the diagnostic accuracy network meta-analysis. Nodes represent the index tests and the reference standard, and connecting lines indicate direct evidence contributed by the included studies. The diagnostic strategies included 1-h PG, HbA1c, FPG, GA, and the combined HbA1c-or-FPG strategy, with OGTT as the reference standard. 1-h PG, 1-hour plasma glucose; HbA1c, glycated hemoglobin; FPG, fasting plasma glucose; GA, glycated albumin; OGTT, oral glucose tolerance test.

The network plot (**Figure 2**) included five diagnostic strategies: 1-h PG, GA, FPG, HbA1c, and the combined HbA1c-or-FPG strategy, all linked through OGTT as the common reference standard. Each node represents a diagnostic strategy, with node size proportional to the corresponding sample size. Lines between nodes indicate direct comparisons, and line thickness is proportional to the number of studies providing such comparisons.

Convergence diagnostics and consistency assessment

Bayesian convergence diagnostics showed that all R-hat values were approximately 1.00, indicating satisfactory convergence. The node-splitting results for sensitivity, specificity, LR+, LR−, and DOR across the five diagnostic strategies are presented in **Supplementary Figure 1**. Overall, the node-splitting results showed no substantial evidence of inconsistency.

#### Diagnostic sensitivity

**Figure 3:**
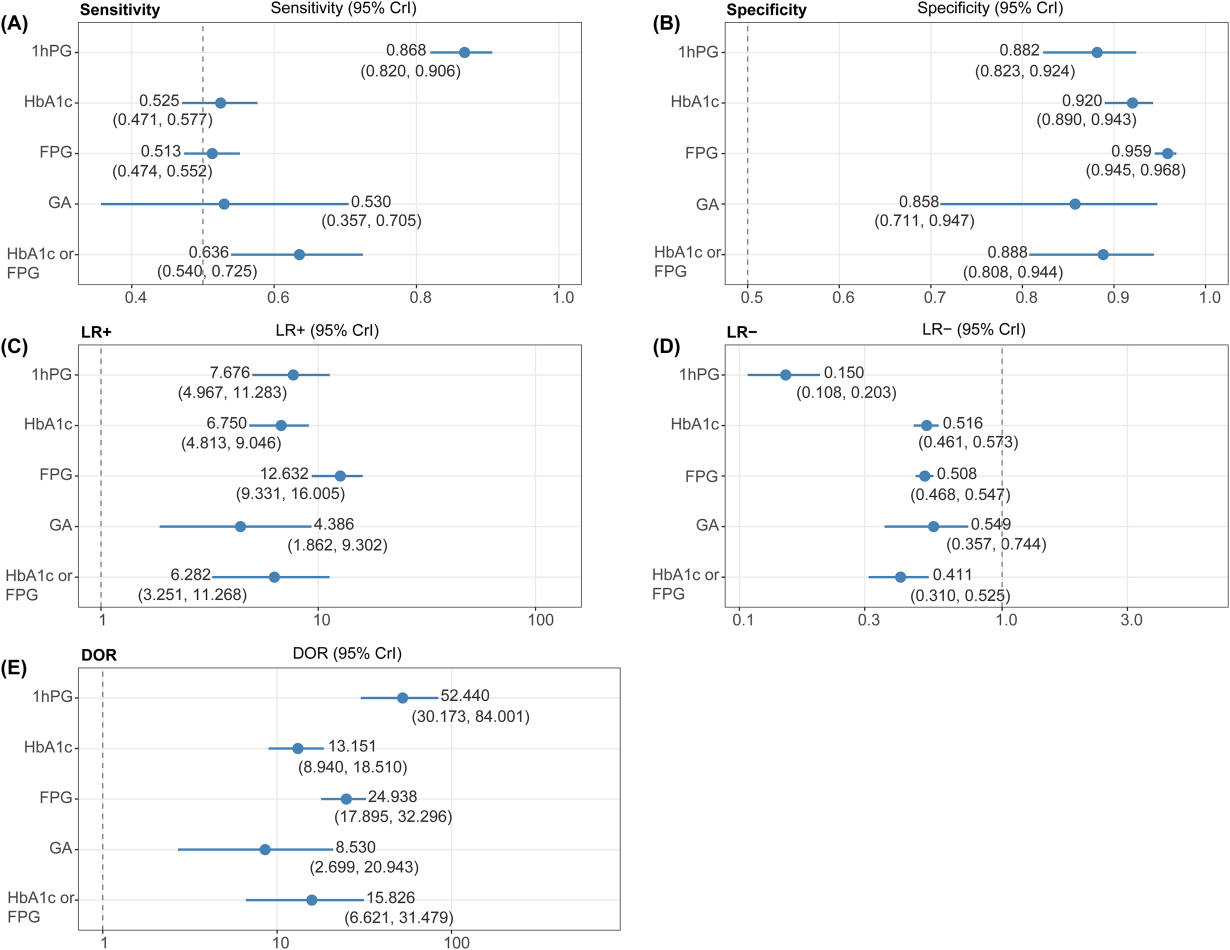
Forest plots from the Bayesian diagnostic test accuracy network meta-analysis showing the pooled posterior mean estimates with 95% credible intervals for sensitivity **(A)**, specificity **(B)**, positive likelihood ratio (LR+) **(C)**, negative likelihood ratio (LR−) **(D)**, and diagnostic odds ratio (DOR) **(E)** across the five diagnostic strategies. 1-h PG, 1-hour plasma glucose; HbA1c, glycated hemoglobin; FPG, fasting plasma glucose; GA, glycated albumin.

**Figure 4:**
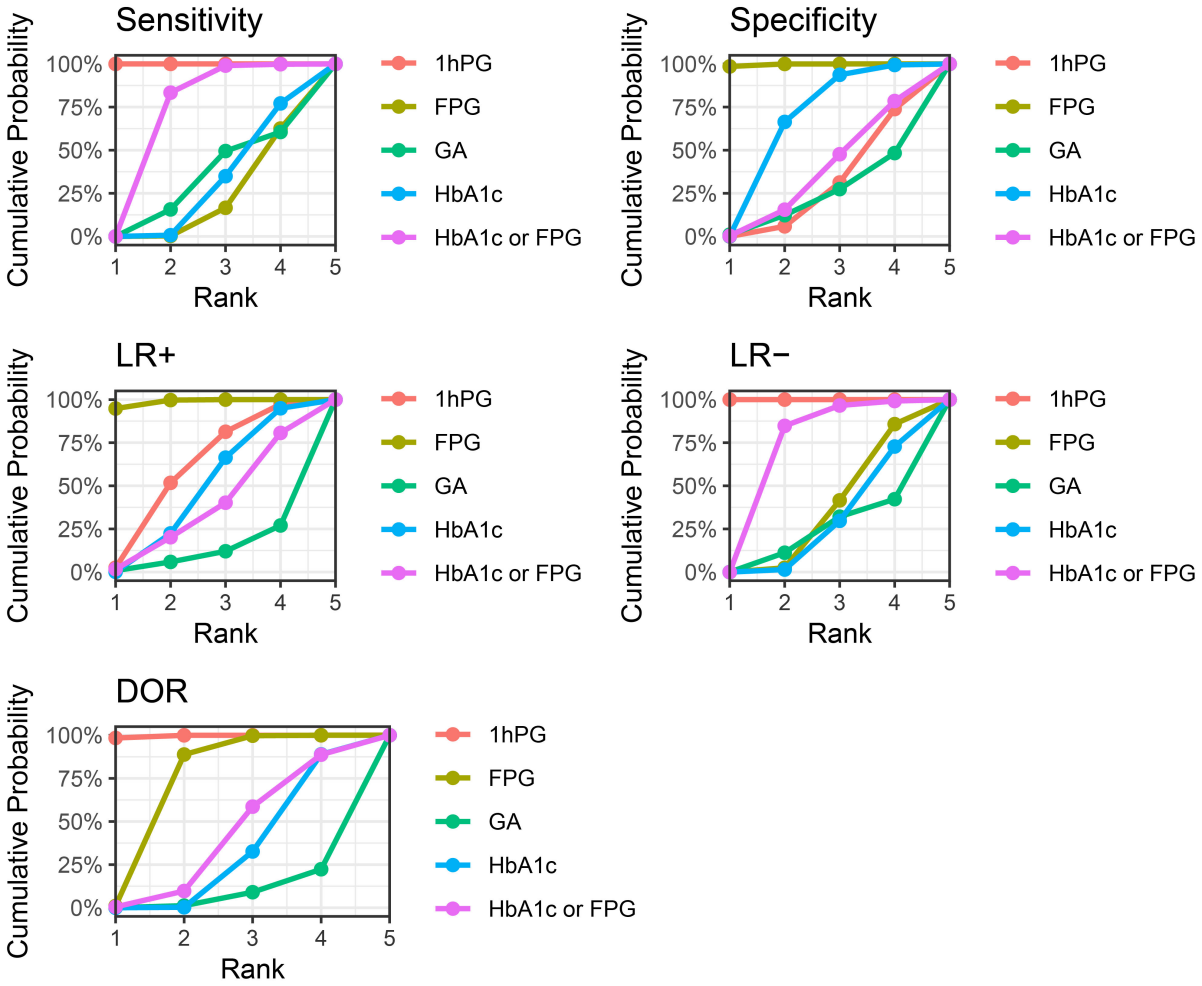
Cumulative ranking curves for the five diagnostic strategies in the Bayesian diagnostic test accuracy network meta-analysis. Ranking probabilities are shown for sensitivity, specificity, positive likelihood ratio (LR+), negative likelihood ratio (LR−), and diagnostic odds ratio (DOR). 1-h PG, 1-hour plasma glucose; HbA1c, glycated hemoglobin; FPG, fasting plasma glucose; GA, glycated albumin.

**Figure 5:**
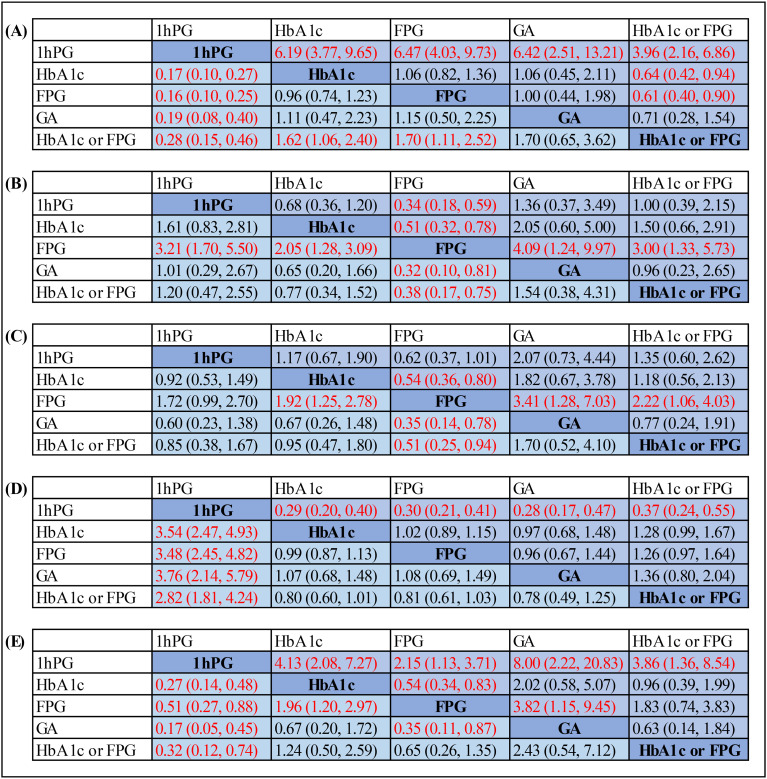
League tables of relative diagnostic performance from the Bayesian diagnostic test accuracy network meta-analysis. Relative comparisons among 1-h PG, HbA1c, FPG, GA, and the combined HbA1c-or-FPG strategy are presented for sensitivity **(A)**, specificity **(B)**, positive likelihood ratio (LR+) **(C)**, negative likelihood ratio (LR−) **(D)**, and diagnostic odds ratio (DOR) **(E)**, with posterior estimates and 95% credible intervals. 1-h PG, 1-hour plasma glucose; HbA1c, glycated hemoglobin; FPG, fasting plasma glucose; GA, glycated albumin.

The network meta-analysis showed that the pooled sensitivity of 1-h PG, GA, FPG, HbA1c, and the combined HbA1c-or-FPG strategy was 0.87 (95% CrI, 0.82–0.91), 0.53 (95% CrI, 0.36–0.71), 0.51 (95% CrI, 0.47–0.55), 0.53 (95% CrI, 0.47–0.58), and 0.64 (95% CrI, 0.54–0.73), respectively (**Figure 3A**). The corresponding SUCRA values were 1.00, 0.31, 0.20, 0.28, and 0.71, respectively (**Figure 4**). The league table for pairwise comparisons is shown in **Figure 5A**. Overall, 1-h PG had the highest sensitivity, followed by the combined HbA1c-or-FPG strategy, whereas the remaining three strategies showed relatively lower sensitivity.

#### Diagnostic specificity

The pooled specificity of 1-h PG, GA, FPG, HbA1c, and the combined HbA1c-or-FPG strategy was 0.88 (95% CrI, 0.82–0.92), 0.86 (95% CrI, 0.71–0.95), 0.96 (95% CrI, 0.95–0.97), 0.92 (95% CrI, 0.89–0.94), and 0.89 (95% CrI, 0.81–0.95), respectively (**Figure 3B**). The corresponding SUCRA values were 0.28, 0.22, 1.00, 0.65, and 0.36 (**Figure 4**). The league table is shown in **Figure 5B**. Overall, FPG had the highest specificity, followed by HbA1c, whereas the remaining strategies showed relatively similar specificity.

#### Positive likelihood ratio

The pooled LR+ of 1-h PG, GA, FPG, HbA1c, and the combined HbA1c-or-FPG strategy was 7.68 (95% CrI, 4.97–11.28), 4.39 (95% CrI, 1.86–9.30), 12.63 (95% CrI, 9.33–16.01), 6.75 (95% CrI, 4.81–9.05), and 6.28 (95% CrI, 3.25–11.26), respectively (**Figure 3C**). The corresponding SUCRA values were 0.58, 0.12, 0.99, 0.46, and 0.36 (**Figure 4**). The league table is shown in **Figure 5C**. Overall, FPG had the highest LR+, suggesting the strongest rule-in performance.

#### Negative likelihood ratio

The pooled LR− of 1-h PG, GA, FPG, HbA1c, and the combined HbA1c-or-FPG strategy was 0.15 (95% CrI, 0.11–0.20), 0.55 (95% CrI, 0.36–0.74), 0.51 (95% CrI, 0.47–0.55), 0.52 (95% CrI, 0.46–0.57), and 0.41 (95% CrI, 0.31–0.53), respectively (**Figure 3D**). The corresponding SUCRA values were 1.00, 0.21, 0.33, 0.26, and 0.70 (**Figure 4**). The league table is shown in **Figure 5D**. Overall, 1-h PG had the lowest LR−, indicating the best rule-out performance, followed by the combined HbA1c-or-FPG strategy.

#### Diagnostic odds ratio

The pooled DORs of 1-h PG, GA, FPG, HbA1c, and the combined HbA1c-or-FPG strategy were 52.44 (95% CrI, 30.17–84.00), 8.53 (95% CrI, 2.70–20.94), 24.94 (95% CrI, 17.90–32.30), 13.15 (95% CrI, 8.94–18.51), and 15.83 (95% CrI, 6.62–31.47), respectively (**Figure 3E**). The corresponding SUCRA values were 1.00, 0.08, 0.72, 0.31, and 0.39 (**Figure 4**). The league table is shown in **Figure 5E**. Overall, 1-h PG had the highest DOR, indicating the best overall diagnostic performance.

#### SROC curve

**Figure 6:**
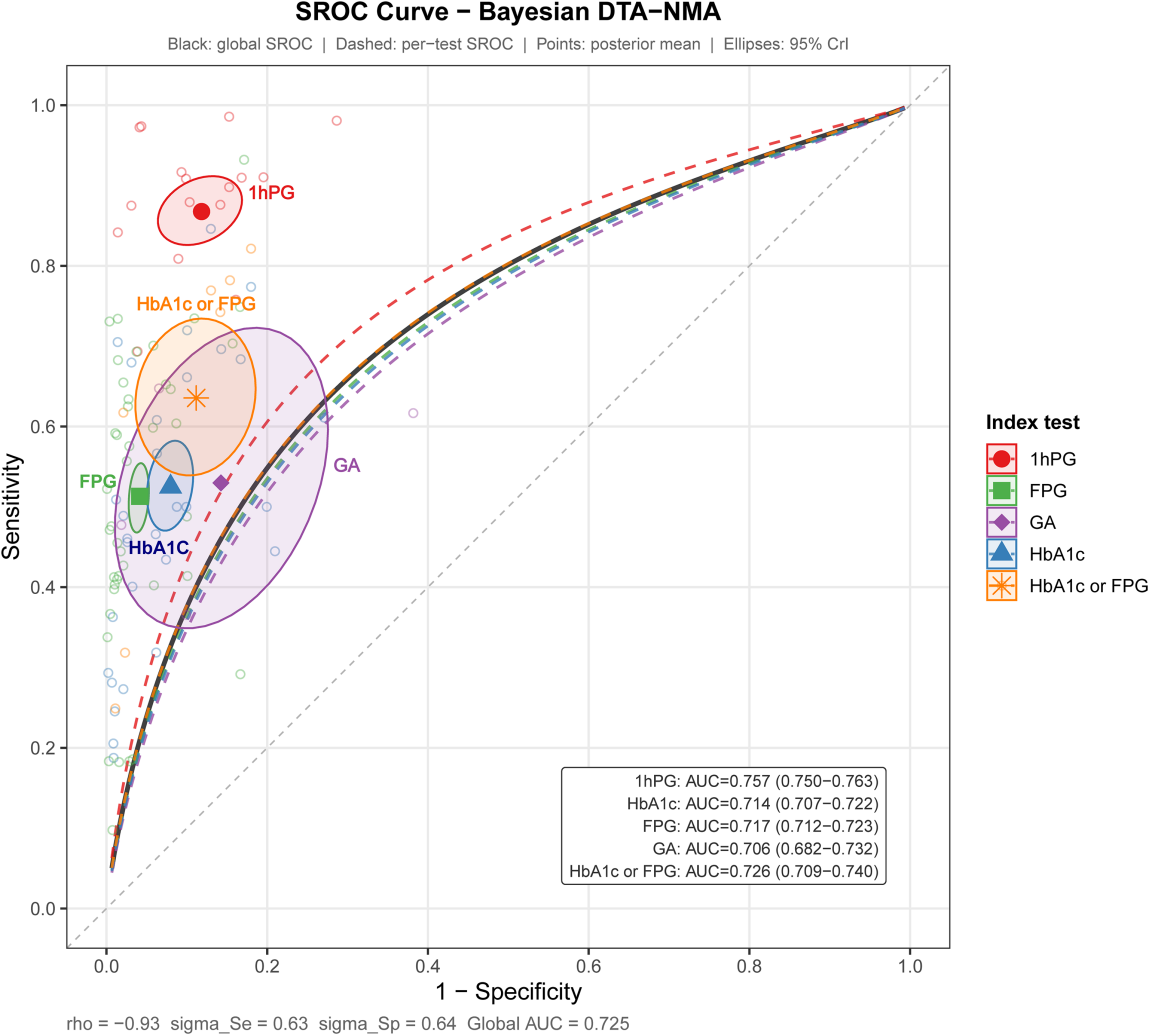
Summary receiver operating characteristic (SROC) curves from the Bayesian diagnostic test accuracy network meta-analysis. The black solid line represents the global SROC curve, dashed lines represent test-specific SROC curves, points indicate posterior mean estimates, and ellipses indicate 95% credible intervals. The figure also presents the area under the curve (AUC) for each diagnostic strategy and the global AUC. 1-h PG, 1-hour plasma glucose; HbA1c, glycated hemoglobin; FPG, fasting plasma glucose; GA, glycated albumin.

The SROC curves showed that 1-h PG had the highest AUC (0.76), indicating the best overall diagnostic performance. It was followed by the combined HbA1c-or-FPG strategy, FPG, HbA1c, and GA (**Figure 6**).

#### Comparative results from the ANOVA model

The ANOVA model showed that 1-h PG had the best overall diagnostic performance, with the highest sensitivity, DOR, and superiority index. FPG had the highest specificity but relatively low sensitivity, whereas HbA1c showed high specificity but limited sensitivity. GA ranked relatively low in terms of both overall diagnostic performance and superiority index. The combined HbA1c-or-FPG strategy improved sensitivity and the superiority index to some extent, but its overall performance remained inferior to that of 1-h PG. Detailed results are presented in **Supplementary Table 2**.

### Pairwise meta-analysis

1-h PG

The pairwise meta-analysis showed that the pooled sensitivity of 1-h PG was 0.91 (95% CI, 0.87–0.94; I² = 87.62%) and the pooled specificity was 0.91 (95% CI, 0.87–0.94; I² = 99.10%) (**Supplementary Figure 2A**). The AUC of the SROC curve was 0.96 (95% CI, 0.94–0.98) (**Supplementary Figure 3A**). The pooled LR+ was 10.16 (95% CI, 6.95–14.85; I² = 98.65%), the pooled LR− was 0.10 (95% CI, 0.07–0.15; I² = 85.31%) (**Supplementary Figure 4A**), and the pooled DOR was 103.02 (95% CI, 58.08–182.72) (**Supplementary Figure 5A**), indicating high diagnostic accuracy for T2DM, although substantial between-study heterogeneity was present. The Fagan nomogram suggested potential clinical utility (**Supplementary Figure 6A**). Deeks’ funnel plot asymmetry test showed no significant publication bias (P = 0.46) (**Supplementary Figure 7A**). The likelihood-ratio scatter plot suggested that 1-h PG had both rule-in and rule-out value for T2DM (**Supplementary Figure 8A**).

The pairwise meta-analysis showed that the pooled sensitivity of GA was 0.53 (95% CI, 0.41–0.64; I² = 90.14%) and the pooled specificity was 0.91 (95% CI, 0.75–0.97; I² = 98.81%) (**Supplementary Figure 2B**). The AUC of the SROC curve was 0.70 (95% CI, 0.66–0.74) (**Supplementary Figure 3B**). The pooled LR+ was 6.11 (95% CI, 2.07–18.07; I² = 96.58%), the pooled LR− was 0.52 (95% CI, 0.40–0.66; I² = 86.03%) (**Supplementary Figure 4B**), and the pooled DOR was 11.81 (95% CI, 3.60–38.70) (**Supplementary Figure 5B**), indicating modest diagnostic accuracy for T2DM, with substantial heterogeneity across studies. The Fagan nomogram suggested limited clinical utility (**Supplementary Figure 6B**). Deeks’ funnel plot asymmetry test showed no significant publication bias (P = 0.06) (**Supplementary Figure 7B**). The likelihood-ratio scatter plot suggested that GA had limited value as a stand-alone rule-in or rule-out test for T2DM (**Supplementary Figure 8B**).

FPG

The pairwise meta-analysis showed that the pooled sensitivity of FPG was 0.51 (95% CI, 0.44–0.57; I² = 97.06%) and the pooled specificity was 0.98 (95% CI, 0.97–0.98; I² = 99.33%) (**Supplementary Figure 2C**). The AUC of the SROC curve was 0.89 (95% CI, 0.86–0.92) (**Supplementary Figure 3C**). The pooled LR+ was 22.85 (95% CI, 16.34–31.96; I² = 98.40%), the pooled LR− was 0.50 (95% CI, 0.44–0.57; I² = 98.48%) (**Supplementary Figure 4C**), and the pooled DOR was 45.44 (95% CI, 32.06–64.40) (**Supplementary Figure 5C**), indicating high specificity and moderate diagnostic accuracy for T2DM, although sensitivity was relatively low and heterogeneity was substantial. The Fagan nomogram suggested that a positive result had good clinical utility, whereas a negative result had limited value for ruling out T2DM (**Supplementary Figure 6C**). Deeks’ funnel plot asymmetry test showed no significant publication bias (P = 0.29) (**Supplementary Figure 7C**). The likelihood-ratio scatter plot indicated that FPG was more suitable as a rule-in test than as a rule-out test (**Supplementary Figure 8C**).

HbA1c

The pairwise meta-analysis showed that the pooled sensitivity of HbA1c was 0.50 (95% CI, 0.43–0.57; I² = 97.19%) and the pooled specificity was 0.96 (95% CI, 0.94–0.97; I² = 99.37%) (**Supplementary Figure 2D**). The AUC of the SROC curve was 0.83 (95% CI, 0.79–0.86) (**Supplementary Figure 3D**). The pooled LR+ was 11.98 (95% CI, 8.36–17.18; I² = 98.03%), the pooled LR− was 0.52 (95% CI, 0.46–0.59; I² = 97.33%) (**Supplementary Figure 4D**), and the pooled DOR was 23.06 (95% CI, 16.30–32.61) (**Supplementary Figure 5D**), indicating moderate diagnostic accuracy for T2DM, with high specificity but limited sensitivity and substantial heterogeneity. The Fagan nomogram suggested reasonable rule-in value but limited rule-out value (**Supplementary Figure 6D**). Deeks’ funnel plot asymmetry test showed no significant publication bias (P = 0.53) (**Supplementary Figure 7D**). The likelihood-ratio scatter plot indicated that HbA1c had good confirmatory value but was not suitable as a stand-alone rule-out test (**Supplementary Figure 8D**).

Combined HbA1c-or-FPG strategy

The pairwise meta-analysis showed that the pooled sensitivity of the combined HbA1c-or-FPG strategy was 0.63 (95% CI, 0.45–0.78; I² = 99.11%) and the pooled specificity was 0.94 (95% CI, 0.86–0.97; I² = 99.51%) (**Supplementary Figure 2E**). The AUC of the SROC curve was 0.89 (95% CI, 0.86–0.91) (**Supplementary Figure 3E**). The pooled LR+ was 9.88 (95% CI, 5.67–17.23; I² = 97.44%), the pooled LR− was 0.40 (95% CI, 0.27–0.60; I² = 99.42%) (**Supplementary Figure 4E**), and the pooled DOR was 24.71 (95% CI, 17.71–34.47) (**Supplementary Figure 5E**), indicating favorable diagnostic accuracy for T2DM, although substantial heterogeneity remained. The Fagan nomogram suggested some clinical utility, but its rule-out value was still limited (**Supplementary Figure 6E**). Deeks’ funnel plot asymmetry test showed no significant publication bias (P = 0.21) (**Supplementary Figure 7E**). The likelihood-ratio scatter plot indicated that the combined HbA1c-or-FPG strategy had good rule-in value but limited rule-out performance (**Supplementary Figure 8E**).

### Subgroup analyses

The network plot (**Supplementary Figures 9A–C**) included 1-h PG, GA, FPG, HbA1c, and the combined HbA1c-or-FPG strategy, all linked through OGTT.

Studies with a 1-h PG threshold ≥11.6 mmol/L

After restricting the analysis to studies using a 1-h PG threshold ≥11.6 mmol/L, the network meta-analysis results were generally consistent with the primary analysis. Only minor changes were observed in the SUCRA ranking for specificity, whereas the other metrics remained essentially unchanged (**Supplementary Figure 10**, **11**; **Supplementary Tables 3A–E**). Overall, the results remained robust under this restriction.

Studies in general populations

After restricting the analysis to studies conducted in general populations, the network meta-analysis results were broadly consistent with the primary analysis. Only slight changes were observed in the SUCRA rankings for sensitivity and specificity, whereas the other indicators remained largely unchanged (**Supplementary Figures 12**, **13**; **Supplementary Tables 4A–E**). Overall, the findings remained robust in this subgroup.

Studies in high-risk populations

After restricting the analysis to studies conducted in high-risk populations, the SUCRA rankings for sensitivity, specificity, LR+, and LR− changed slightly compared with the primary analysis, but the numerical differences were small. The ranking for DOR remained unchanged (**Supplementary Figures 14**, **15**; **Supplementary Tables 5A–E**). Among the evaluated strategies, GA showed relatively greater changes in the effect estimates and 95% CrIs for sensitivity and LR+; however, the overall findings remained broadly consistent with those of the primary analysis.

Sensitivity analysis restricted to low-risk-of-bias studies

The network plot (**Supplementary Figure 16**) included 1-h PG, GA, FPG, HbA1c, and the combined HbA1c-or-FPG strategy, all linked through OGTT.

In the sensitivity analysis restricted to studies at low risk of bias, the network meta-analysis was repeated. The results showed slight changes in the SUCRA rankings for sensitivity, specificity, and LR+ compared with the primary analysis, although the magnitude of these changes was small. The rankings for LR− and DOR remained unchanged. The forest plots for GA showed some differences in sensitivity, specificity, and LR+ relative to the primary analysis, suggesting a degree of variability in its effect estimates (**Supplementary Figures 17**, **18**; **Supplementary Tables 6A–E**).

## Discussion

T2DM typically develops after a prolonged period of subclinical dysglycemia, and different diagnostic measures capture distinct aspects of glucose dysregulation (18). FPG mainly reflects glucose homeostasis under fasting steady-state conditions, whereas HbA1c and GA reflect average glycemic exposure over different time windows (19, 20). In contrast, 1-h PG and 2-h PG measured during OGTT reflect the dynamic metabolic response to a glucose challenge (3, 7). Recent evidence suggests that a single measure may not adequately identify individuals at an early stage of disease, particularly those with relatively normal fasting glucose but abnormal post-load glycemia (21). Therefore, a systematic comparison of the diagnostic performance of different measures is of clear importance for the screening and diagnosis of T2DM. In the present study, using 2-h PG ≥200 mg/dL during a 75-g OGTT as the common reference standard, we applied an ANOVA-based network meta-analysis to comprehensively compare the diagnostic accuracy of 1-h PG, GA, FPG, HbA1c, and the combined HbA1c-or-FPG strategy. The results showed that 1-h PG performed best in terms of sensitivity, LR−, and DOR, whereas FPG showed advantages in specificity and LR +. The overall performance of the combined HbA1c-or-FPG strategy lay between these two approaches, while GA showed relatively limited overall diagnostic performance. Taken together, these findings indicate that no single measure is uniformly superior across all diagnostic dimensions; rather, the relative advantage of each measure depends on the intended diagnostic purpose. In this context, 1-h PG appears to be more suitable for early detection and reducing missed diagnoses, whereas FPG appears to be more suitable for confirmatory diagnosis. Accordingly, the main contribution of this study is not to establish an absolute hierarchy of diagnostic strategies, but to provide evidence for selecting diagnostic approaches according to different clinical needs.

These findings are broadly consistent with the pathophysiology of dysglycemia in diabetes. Because 1-h PG reflects the early glycemic response after glucose loading, it is more likely to identify impaired first-phase insulin secretion and early postprandial hyperglycemia, which may explain its superior sensitivity and lower LR− (7, 22). By contrast, FPG reflects glucose levels under fasting steady-state conditions, and values reaching the diagnostic threshold often indicate more overt metabolic disturbance; this may account for its higher specificity and LR+ (23, 24). HbA1c reflects longer-term average glycemia, whereas GA reflects shorter-term average glycemia (5, 25). Although both provide integrated glycemic information, their ability to detect early abnormalities in glucose metabolism may be relatively limited (26, 27). The combined HbA1c-or-FPG strategy showed higher sensitivity than either FPG or HbA1c alone, suggesting that combining measures may improve case detection to some extent (4). However, its overall diagnostic performance did not exceed that of 1-h PG.

Our findings are also generally consistent with previous meta-analyses. A previous meta-analysis that evaluated the diagnostic accuracy of 1-h PG against 2-h PG during OGTT showed that 1-h PG had good diagnostic accuracy for T2DM in adults and suggested that 11.6 mmol/L may be an appropriate diagnostic cutoff (6). In 2024, the IDF issued a statement proposing that 1-h PG ≥11.6 mmol/L during a 75-g OGTT may be used as a diagnostic threshold for T2DM (7). A recent pooled analysis including five independent cohorts and 11,968 participants further supported this view, reporting pooled AUC, sensitivity, and specificity values of 0.97, 88.9%, and 98.5%, respectively, for 1-h PG. In that analysis, the AUC of 1-h PG was substantially higher than that of the combined FPG-plus-HbA1c strategy (0.85), and its overall diagnostic performance was superior to that of FPG, 2-h PG, HbA1c, and their combined strategies (28). At present, the ADA still regards FPG, 2-h PG, and HbA1c as the principal diagnostic criteria for T2DM (29). Taken together, these data suggest that 1-h PG is already supported by a growing body of evidence and that current research is strengthening its diagnostic value. However, its place within standardized diagnostic pathways still appears to be in transition from research evidence to routine clinical application. Therefore, the finding of superior overall diagnostic performance for 1-h PG in the present study should currently be interpreted as supporting a complementary role alongside existing major diagnostic strategies, rather than as evidence for replacing conventional diagnostic measures.

Compared with previous meta-analyses, the main value of this study lies in the inclusion of a broader range of diagnostic strategies, the integrated comparison across these strategies, and the relatively comprehensive subgroup and sensitivity analyses. Earlier conventional meta-analyses mainly focused on whether a single measure had diagnostic value and on the magnitude of that value, whereas the previous network meta-analysis compared only the relative performance of FPG, HbA1c, and the combined HbA1c-or-FPG strategy (4, 6, 10, 26). In the present study, we restricted the analysis to adults aged ≥18 years and used OGTT as the common reference standard to compare 1-h PG, GA, FPG, HbA1c, and the combined HbA1c-or-FPG strategy within a single evidence network. This allowed a clearer evaluation of the relative position and role of each diagnostic strategy in clinical decision-making. A previous network meta-analysis showed that FPG had advantages in specificity and LR+, whereas the combined HbA1c-or-FPG strategy improved sensitivity to some extent (4). By additionally incorporating 1-h PG and GA, our study confirmed that the advantage of FPG in confirmatory diagnosis remained stable, while 1-h PG showed superior overall diagnostic performance. Moreover, we extended previous work by performing subgroup analyses according to population risk, subgroup analyses based on the 1-h PG threshold, and analyses restricted to studies at low risk of bias, thereby further examining the robustness of the network meta-analysis findings. In addition, we conducted pairwise meta-analyses for all five measures, and the results were broadly consistent with those of the network meta-analysis, which indirectly supports the reliability of the overall conclusions.

The findings for GA deserve particular attention. In the present study, GA was not advantageous in terms of sensitivity, LR−, or DOR, and its overall diagnostic performance was inferior to that of the other evaluated strategies. Previous meta-analyses have suggested that GA has some diagnostic value for diabetes; however, the included studies used inconsistent thresholds and showed substantial heterogeneity (10). This suggests that the diagnostic performance of GA may depend on the threshold applied, the population under study, and the testing context. At present, the available evidence does not support GA as an independent diagnostic measure for diabetes (30). Nevertheless, it may still serve as a complementary measure in situations in which HbA1c is less reliable, such as disorders affecting erythrocyte lifespan or the presence of hemoglobin variants (31). Given the limited number of relevant studies, the results for GA remain somewhat unstable and should therefore be interpreted cautiously.

The interpretation of our findings should also take into account the pooled estimates, the comparability of the evaluated strategies, and the consistency between direct and indirect evidence. By adopting 2-h PG ≥200 mg/dL during a 75-g OGTT as the common reference standard, our study improved comparability across diagnostic strategies and enhanced internal consistency within the evidence network. At the same time, however, this choice necessarily narrows the scope of interpretation. More specifically, our analysis compared the degree of agreement between different diagnostic measures and the T2DM definition based on 2-h PG as the reference standard, rather than comparing the absolute diagnostic capability of each measure in a broader conceptual sense. Because 1-h PG and 2-h PG are dynamic glycemic measures, whereas FPG reflects fasting glycemia, HbA1c reflects longer-term average glycemia, and GA reflects shorter-term average glycemia, these measures do not convey identical physiological information (32–34). Accordingly, the ranking results should be interpreted within the context of this shared reference framework, rather than as a universal ordering of intrinsic diagnostic superiority.

Several limitations should be acknowledged. First, most included studies were cross-sectional, and residual bias related to patient selection, ethnicity, methodological quality, and diagnostic thresholds cannot be excluded. Second, the number of studies evaluating 1-h PG, and especially GA and the combined HbA1c-or-FPG strategy, was relatively limited, which may have affected the precision of pooled estimates and the stability of rankings. Third, although the included studies covered multiple countries and ethnic groups, inconsistent or unclear reporting of ethnicity limited the feasibility of ethnicity-based subgroup analyses. Fourth, thresholds were not fully consistent within the same diagnostic category. Although FPG ≥126 mg/dL and HbA1c ≥6.5% are well-established thresholds, standardized thresholds for 1-h PG and GA remain less certain. Therefore, the subgroup analysis restricted to studies using a 1-h PG threshold ≥11.6 mmol/L should not be interpreted as defining universally applicable cutoffs. Fifth, limitations in the standardization and reproducibility of OGTT across studies may have affected the stability and accuracy of the results. Sixth, we used the ANOVA model proposed. for the diagnostic test accuracy network meta-analysis. This published model provides a practical and transparent framework for synthesizing direct and indirect evidence and comparing multiple diagnostic strategies within a connected network, which is consistent with the aim of our study. Nevertheless, as with other diagnostic accuracy models, sensitivity and specificity may be correlated, particularly in the presence of threshold effects. Therefore, the model-based rankings and comparative estimates should be interpreted together with LR+, LR−, DOR, SROC curves, credible intervals, heterogeneity, consistency assessments, subgroup analyses, and sensitivity analyses. Seventh, although the node-splitting analysis generally supported the consistency assumption, some node comparisons showed inconsistency. Finally, although the sensitivity, subgroup, and pairwise meta-analyses were generally consistent with the primary analysis, substantial between-study heterogeneity remained. These factors may have affected network transitivity and the stability of the conclusions. Therefore, the relative ranking of diagnostic measures should not be interpreted solely on the basis of point estimates, but should be judged together with the stability and consistency of the available evidence.

The applicability of our findings is also bounded. They are primarily relevant to the diagnosis and screening of previously undiagnosed diabetes in adults, including both general and high-risk populations. The results should not be directly extrapolated to patients with type 1 diabetes, gestational diabetes mellitus, cystic fibrosis-related diabetes, or individuals already receiving glucose-lowering therapy. In addition, the findings for HbA1c and GA should be interpreted cautiously in special populations with severe anemia, hemoglobinopathies, hypoalbuminemia, or liver dysfunction. From the perspective of clinical implementation, the main advantage of 1-h PG lies in settings where OGTT is feasible, where shortening the testing time relative to 2-h PG is desirable, and where the clinical objective is early identification and reduction of missed diagnosis (35). However, in settings where OGTT is impractical, FPG and HbA1c remain highly applicable, and the combined HbA1c-or-FPG strategy may improve sensitivity to some extent (36, 37). Thus, the statistical advantage of 1-h PG does not imply that it can directly replace existing standards; rather, it may be better viewed as a means of supplementing and optimizing current diagnostic pathways.

In conclusion, when 2-h PG ≥200 mg/dL during a 75-g OGTT is used as the common reference standard, 1-h PG appears to have superior overall diagnostic performance among the evaluated strategies, supporting its potential value in the early diagnosis of T2DM. FPG showed clear advantages in specificity and rule-in performance, underscoring its important role in confirmatory diagnosis. The combined HbA1c-or-FPG strategy may improve case detection to some extent, whereas GA may have a complementary role under selected circumstances. Although current evidence supports the overall diagnostic value of 1-h PG, further high-quality studies are still needed to better define its applicability across populations, its clinical boundaries, and its role within diagnostic pathways. Future optimization of T2DM diagnostic strategies should be based on integrated consideration of diagnostic purpose, implementation setting, and population characteristics.

## Data Availability

The original contributions presented in the study are included in the article/**Supplementary Material**. Further inquiries can be directed to the corresponding authors.
