# Supplementary material for: Comparative accuracy of 1-hour post-load plasma glucose, glycated albumin, and conventional glycemic measures for the diagnosis of type 2 diabetes mellitus: a systematic review and network meta-analysis
Source: Front Endocrinol (Lausanne). 2026 Jul 8;17:1846553. doi: 10.3389/fendo.2026.1846553 (PMC13388173; doi:10.3389/fendo.2026.1846553)
Supplement: Supplementary file 1 [file DataSheet1.docx]

**Supplementary File S1.** Detailed search strategies for each database

**PubMed**

((("Diabetes Mellitus"[Mesh])

OR (("Diabetes Mellitus"[Title/Abstract]) OR (diabetes[Title/Abstract]) OR (diabetic[Title/Abstract])))

AND

((("Glucose Tolerance Test"[Mesh])

OR ("Blood Glucose"[Mesh])

OR ("Glycated Hemoglobin"[Mesh])

OR ("Glycated Serum Albumin"[Mesh]))

OR (("Glucose Tolerance Test"[Title/Abstract])

OR ("Blood Glucose"[Title/Abstract])

OR ("Glycated Hemoglobin"[Title/Abstract])

OR ("Glycated Serum Albumin"[Title/Abstract])

OR ("1-hour plasma glucose"[Title/Abstract])

OR ("1 hour plasma glucose"[Title/Abstract])

OR ("1h plasma glucose"[Title/Abstract])

OR ("1-h plasma glucose"[Title/Abstract])

OR ("1-h PG"[Title/Abstract])

OR ("1h PG"[Title/Abstract])

OR ("1-hour glucose"[Title/Abstract])

OR ("1 hour glucose"[Title/Abstract])

OR ("1-h glucose"[Title/Abstract])

OR ("1h glucose"[Title/Abstract])

OR ("one-hour glucose"[Title/Abstract])

OR ("one hour glucose"[Title/Abstract])

OR ("glycated albumin"[Title/Abstract])

OR ("glycosylated albumin"[Title/Abstract])

OR (HbA1c[Title/Abstract])

OR ("Hemoglobin A1c"[Title/Abstract])

OR ("Haemoglobin A1c"[Title/Abstract])

OR ("glycosylated hemoglobin"[Title/Abstract])

OR ("Fasting plasma glucose"[Title/Abstract])

OR ("Fasting blood glucose"[Title/Abstract])

OR (FPG[Title/Abstract])

OR (FBG[Title/Abstract])))

AND

((("Sensitivity and Specificity"[Mesh])

OR ("Mass Screening"[Mesh])

OR ("Predictive Value of Tests"[Mesh])

OR ("ROC Curve"[Mesh]))

OR (("Sensitivity and Specificity"[Title/Abstract])

OR ("Mass Screening"[Title/Abstract])

OR ("Predictive Value of Tests"[Title/Abstract])

OR ("ROC Curve"[Title/Abstract])

OR (sensitivity[Title/Abstract])

OR (specificity[Title/Abstract])

OR ("diagnostic accuracy"[Title/Abstract])

OR (screening[Title/Abstract])

OR ("false positive"[Title/Abstract])

OR ("false negative"[Title/Abstract])

OR ("predictive value"[Title/Abstract])

OR (ROC[Title/Abstract])

OR (AUC[Title/Abstract])

OR ("diagnostic test"[Title/Abstract])))

**Embase**

('diabetes mellitus'/exp

OR 'diabetes mellitus':ti,ab

OR diabetes:ti,ab

OR diabetic:ti,ab)

AND

('glucose tolerance test'/exp

OR 'blood glucose'/exp

OR 'glycosylated hemoglobin'/exp

OR 'glycated serum albumin'/exp

OR 'glucose tolerance test':ti,ab

OR 'blood glucose':ti,ab

OR 'glycated hemoglobin':ti,ab

OR 'glycated serum albumin':ti,ab

OR '1-hour plasma glucose':ti,ab

OR '1 hour plasma glucose':ti,ab

OR '1h plasma glucose':ti,ab

OR '1-h plasma glucose':ti,ab

OR '1-h pg':ti,ab

OR '1h pg':ti,ab

OR '1-hour glucose':ti,ab

OR '1 hour glucose':ti,ab

OR '1-h glucose':ti,ab

OR '1h glucose':ti,ab

OR 'one-hour glucose':ti,ab

OR 'one hour glucose':ti,ab

OR 'glycated albumin':ti,ab

OR 'glycosylated albumin':ti,ab

OR hba1c:ti,ab

OR 'hemoglobin a1c':ti,ab

OR 'haemoglobin a1c':ti,ab

OR 'glycosylated hemoglobin':ti,ab

OR 'fasting plasma glucose':ti,ab

OR 'fasting blood glucose':ti,ab

OR fpg:ti,ab

OR fbg:ti,ab)

AND

('sensitivity and specificity'/exp

OR 'mass screening'/exp

OR 'predictive value'/exp

OR 'roc curve'/exp

OR 'sensitivity and specificity':ti,ab

OR 'mass screening':ti,ab

OR 'predictive value of tests':ti,ab

OR 'predictive value':ti,ab

OR 'roc curve':ti,ab

OR sensitivity:ti,ab

OR specificity:ti,ab

OR 'diagnostic accuracy':ti,ab

OR screening:ti,ab

OR 'false positive':ti,ab

OR 'false negative':ti,ab

OR roc:ti,ab

OR auc:ti,ab

OR 'diagnostic test':ti,ab)

**Web of Science**

((TI=("diabetes mellitus" OR diabetes OR diabetic)

OR AB=("diabetes mellitus" OR diabetes OR diabetic))

AND

(TI=("glucose tolerance test" OR "blood glucose" OR "glycated hemoglobin" OR "glycated serum albumin" OR "1-hour plasma glucose" OR "1 hour plasma glucose" OR "1h plasma glucose" OR "1-h plasma glucose" OR "1-h pg" OR "1h pg" OR "1-hour glucose" OR "1 hour glucose" OR "1-h glucose" OR "1h glucose" OR "one-hour glucose" OR "one hour glucose" OR "glycated albumin" OR "glycosylated albumin" OR HbA1c OR "hemoglobin A1c" OR "haemoglobin A1c" OR "glycosylated hemoglobin" OR "fasting plasma glucose" OR "fasting blood glucose" OR FPG OR FBG)

OR

AB=("glucose tolerance test" OR "blood glucose" OR "glycated hemoglobin" OR "glycated serum albumin" OR "1-hour plasma glucose" OR "1 hour plasma glucose" OR "1h plasma glucose" OR "1-h plasma glucose" OR "1-h pg" OR "1h pg" OR "1-hour glucose" OR "1 hour glucose" OR "1-h glucose" OR "1h glucose" OR "one-hour glucose" OR "one hour glucose" OR "glycated albumin" OR "glycosylated albumin" OR HbA1c OR "hemoglobin A1c" OR "haemoglobin A1c" OR "glycosylated hemoglobin" OR "fasting plasma glucose" OR "fasting blood glucose" OR FPG OR FBG))

AND

(TI=("sensitivity and specificity" OR "mass screening" OR "predictive value of tests" OR "predictive value" OR "ROC curve" OR sensitivity OR specificity OR "diagnostic accuracy" OR screening OR "false positive" OR "false negative" OR ROC OR AUC OR "diagnostic test")

OR

AB=("sensitivity and specificity" OR "mass screening" OR "predictive value of tests" OR "predictive value" OR "ROC curve" OR sensitivity OR specificity OR "diagnostic accuracy" OR screening OR "false positive" OR "false negative" OR ROC OR AUC OR "diagnostic test")))

**the Cochrane Library**

#1 MeSH descriptor: [Diabetes Mellitus] explode all trees

#2 ("diabetes mellitus":ti,ab OR diabetes:ti,ab OR diabetic:ti,ab)

#3 #1 OR #2

#4 MeSH descriptor: [Glucose Tolerance Test] explode all trees

#5 MeSH descriptor: [Blood Glucose] explode all trees

#6 MeSH descriptor: [Glycated Hemoglobin] explode all trees

#7 MeSH descriptor: [Glycated Serum Albumin] explode all trees

#8 ("glucose tolerance test":ti,ab OR "blood glucose":ti,ab OR "glycated hemoglobin":ti,ab OR "glycated serum albumin":ti,ab OR "1-hour plasma glucose":ti,ab OR "1 hour plasma glucose":ti,ab OR "1h plasma glucose":ti,ab OR "1-h plasma glucose":ti,ab OR "1-h pg":ti,ab OR "1h pg":ti,ab OR "1-hour glucose":ti,ab OR "1 hour glucose":ti,ab OR "1-h glucose":ti,ab OR "1h glucose":ti,ab OR "one-hour glucose":ti,ab OR "one hour glucose":ti,ab OR "glycated albumin":ti,ab OR "glycosylated albumin":ti,ab OR HbA1c:ti,ab OR "hemoglobin A1c":ti,ab OR "haemoglobin A1c":ti,ab OR "glycosylated hemoglobin":ti,ab OR "fasting plasma glucose":ti,ab OR "fasting blood glucose":ti,ab OR FPG:ti,ab OR FBG:ti,ab)

#9 #4 OR #5 OR #6 OR #7 OR #8

#10 MeSH descriptor: [Sensitivity and Specificity] explode all trees

#11 MeSH descriptor: [Mass Screening] explode all trees

#12 MeSH descriptor: [Predictive Value of Tests] explode all trees

#13 MeSH descriptor: [ROC Curve] explode all trees

#14 ("sensitivity and specificity":ti,ab OR "mass screening":ti,ab OR "predictive value of tests":ti,ab OR "predictive value":ti,ab OR "ROC curve":ti,ab OR sensitivity:ti,ab OR specificity:ti,ab OR "diagnostic accuracy":ti,ab OR screening:ti,ab OR "false positive":ti,ab OR "false negative":ti,ab OR ROC:ti,ab OR AUC:ti,ab OR "diagnostic test":ti,ab)

#15 #10 OR #11 OR #12 OR #13 OR #14

#16 #3 AND #9 AND #15

**Scopus**

TITLE-ABS(

("diabetes mellitus" OR diabetes OR diabetic)

AND

("glucose tolerance test" OR "blood glucose" OR "glycated hemoglobin" OR "glycated serum albumin" OR "1-hour plasma glucose" OR "1 hour plasma glucose" OR "1h plasma glucose" OR "1-h plasma glucose" OR "1-h pg" OR "1h pg" OR "1-hour glucose" OR "1 hour glucose" OR "1-h glucose" OR "1h glucose" OR "one-hour glucose" OR "one hour glucose" OR "glycated albumin" OR "glycosylated albumin" OR HbA1c OR "hemoglobin A1c" OR "haemoglobin A1c" OR "glycosylated hemoglobin" OR "fasting plasma glucose" OR "fasting blood glucose" OR FPG OR FBG)

AND

("sensitivity and specificity" OR "mass screening" OR "predictive value of tests" OR "predictive value" OR "ROC curve" OR sensitivity OR specificity OR "diagnostic accuracy" OR screening OR "false positive" OR "false negative" OR ROC OR AUC OR "diagnostic test")

)

**Supplementary File S2.** Citation details of the included studies

1. Tripathy D, Carlsson M, Almgren P, et al. Insulin secretion and insulin sensitivity in relation to glucose tolerance: lessons from the Botnia Study. Diabetes. 2000;49:975–980.

2. Succurro E, Arturi F, Caruso V, et al. Low insulin-like growth factor-1 levels are associated with anaemia in adult non-diabetic subjects. Thromb Haemost. 2011;105:365–370.

3. Abdul-Ghani MA, Abdul-Ghani T, Müller G, et al. Role of glycated hemoglobin in the prediction of future risk of T2DM. J Clin Endocrinol Metab. 2011;96:2596–2600.

4. La Sala L, Mrakic-Sposta S, Tagliabue E, et al. Circulating microRNA-21 is an early predictor of ROS-mediated damage in subjects with high risk of developing diabetes and in drug-naïve T2D. Cardiovasc Diabetol. 2019;18:18.

5. Bianchi C, Miccoli R, Bonadonna RC, et al. Pathogenetic mechanisms and cardiovascular risk: differences between HbA1c and oral glucose tolerance test for the diagnosis of glucose tolerance. Diabetes Care. 2012;35:2607–2612. doi:10.2337/dc11-2504.

6. Bergman M, Chetrit A, Roth J, Dankner R. One-hour post-load plasma glucose level during the OGTT predicts mortality: observations from the Israel Study of Glucose Intolerance, Obesity and Hypertension. Diabet Med. 2016;33:1060–1066.

7. Pyörälä M, Miettinen H, Laakso M, Pyörälä K. Hyperinsulinemia predicts coronary heart disease risk in healthy middle-aged men: the 22-year follow-up results of the Helsinki Policemen Study. Circulation. 1998;98:398–404.

8. Sai Prasanna N, Amutha A, Pramodkumar TA, et al. The 1 h post-glucose value best predicts future dysglycemia among normal glucose tolerance subjects. J Diabetes Complications. 2017;31:1592–1596.

9. Mutt SJ, Jokelainen J, Sebert S, et al. Vitamin D status and components of metabolic syndrome in older subjects from Northern Finland (latitude 65° North). Nutrients. 2019;11:1229.

10. Paddock E, Looker HC, Piaggi P, Knowler WC, Krakoff J, Chang DC. One-hour plasma glucose compared with two-hour plasma glucose in relation to diabetic retinopathy in American Indians. Diabetes Care. 2018;41:1212–1217.

11. Oka R, Yagi K, Sakurai M, et al. Insulin secretion and insulin sensitivity on the oral glucose tolerance test (OGTT) in middle-aged Japanese. Endocr J. 2012;59:55–64.

12. Abdul-Ghani MA, Williams K, DeFronzo RA, Stern M. What is the best predictor of future type 2 diabetes? Diabetes Care. 2007;30:1544–1548.

13. Kim Y, Han BG; KoGES group. Cohort profile: the Korean Genome and Epidemiology Study (KoGES) Consortium. Int J Epidemiol. 2017;46:e20. doi:10.1093/ije/dyx105.

14. Castro JPFC, Macedo DB, Magalhaes RA, Figueirêdo CMM, Petrola R, Teles MG. Diagnostic performance of 1-hour plasma glucose and glucose curve shape during oral glucose tolerance test: a cross-sectional study in a Brazilian cohort. Diabetol Metab Syndr. 2025;17:428. doi:10.1186/s13098-025-01977-1.

15. Ferrannini G, De Bacquer D, Gyberg V, et al. Saving time by replacing the standardised two-hour oral glucose tolerance test with a one-hour test: validation of a new screening algorithm in patients with coronary artery disease from the ESC-EORP EUROASPIRE V registry. Diabetes Res Clin Pract. 2022;183:109156.

16. Thenmozhi, et al. Enhanced detection of type 2 diabetes mellitus in an at-risk prediabetic population using one-hour oral glucose tolerance test (OGTT): a comparison with HbA1c and two-hour OGTT. Cureus. 2025.

17. Wiener K, Roberts NB. The relative merits of haemoglobin A1c and fasting plasma glucose as first-line diagnostic tests for diabetes mellitus in non-pregnant subjects. Diabet Med. 1998;15:558–563. doi:10.1002/(SICI)1096-9136(199807)15:7<558::AID-DIA669>3.0.CO;2-Q.

18. Tanaka Y, Atsumi Y, Matsuoka K, et al. Usefulness of stable HbA1c for supportive marker to diagnose diabetes mellitus in Japanese subjects. Diabetes Res Clin Pract. 2001;53:41–45. doi:10.1016/S0168-8227(01)00226-1.

19. Adamu AN. Comparative performance of HbA1c 6.5% for FPG ≥7.0 vs 2hr PG ≥11.1 criteria for diagnosis of type 2 diabetes. Afr Health Sci. 2011;11:421–426.

20. Valentine NA, Alhawassi TM, Roberts GW, Vora PP, Stranks SN, Doogue MP. Detecting undiagnosed diabetes using glycated haemoglobin: an automated screening test in hospitalised patients. Med J Aust. 2011;194:160–164. doi:10.5694/j.1326-5377.2011.tb02954.x.

21. Zemlin AE, Matsha TE, Hassan MS, Erasmus RT. HbA1c of 6.5% to diagnose diabetes mellitus--does it work for us?--the Bellville South Africa study. PLoS One. 2011;6:e22558. doi:10.1371/journal.pone.0022558.

22. Homko CJ, Zamora LC, Kerper MM, Mozzoli M, Kresge K, Boden G. A single A1C ≥6.5% accurately identifies type 2 diabetes/impaired glucose tolerance in African Americans. J Prim Care Community Health. 2012;3:235–238. doi:10.1177/2150131911435526.

23. Marini MA, Succurro E, Arturi F, Ruffo MF, Andreozzi F, Sciacqua A, et al. Comparison of A1C, fasting and 2-h post-load plasma glucose criteria to diagnose diabetes in Italian Caucasians. Nutr Metab Cardiovasc Dis. 2012;22:561–566. doi:10.1016/j.numecd.2011.04.009.

24. Adamska E, Waszczeniuk M, Gościk J, Golonko A, Wilk J, Pliszka J, et al. The usefulness of glycated hemoglobin A1c (HbA1c) for identifying dysglycemic states in individuals without previously diagnosed diabetes. Adv Med Sci. 2012;57:296–301. doi:10.2478/v10039-012-0030-x.

25. Lee H, Oh JY, Sung YA, Kim DJ, Kim SH, Kim SG, et al. Optimal hemoglobin A1C cutoff value for diagnosing type 2 diabetes mellitus in Korean adults. Diabetes Res Clin Pract. 2013;99:231–236. doi:10.1016/j.diabres.2012.09.030.

26. Alqahtani N, Khan WA, Alhumaidi MH, Ahmed YA. Use of glycated hemoglobin in the diagnosis of diabetes mellitus and pre-diabetes and role of fasting plasma glucose, oral glucose tolerance test. Int J Prev Med. 2013;4:1025–1029.

27. Franco LJ, Dal Fabbro AL, Martinez EZ, Sartorelli DS, Silva AS, Soares LP, et al. Performance of glycated haemoglobin (HbA1c) as a screening test for diabetes and impaired glucose tolerance (IGT) in a high risk population--the Brazilian Xavante Indians. Diabetes Res Clin Pract. 2014;106:337–342. doi:10.1016/j.diabres.2014.08.027.

28. Karnchanasorn R, Huang J, Ou HY, Feng W, Chuang LM, Chiu KC, et al. Comparison of the current diagnostic criterion of HbA1c with fasting and 2-hour plasma glucose concentration. J Diabetes Res. 2016;2016:6195494. doi:10.1155/2016/6195494.

29. Avilés-Santa ML, Schneiderman N, Savage PJ, Kaplan RC, Teng Y, Pérez CM, et al. Identifying probable diabetes mellitus among Hispanics/Latinos from four U.S. cities: findings from the Hispanic Community Health Study/Study of Latinos. Endocr Pract. 2016;22:1151–1160. doi:10.4158/EP151144.OR.

30. Camacho JE, Shah VO, Schrader R, Wong CS, Burge MR. Performance of A1C versus OGTT for the diagnosis of prediabetes in a community-based screening. Endocr Pract. 2016;22:1288–1295. doi:10.4158/EP161267.OR.

31. Herath HMM, Weerarathna TP, Dahanayake MU, Weerasinghe NP. Use of HbA1c to diagnose type 2 diabetes mellitus among high risk Sri Lankan adults. Diabetes Metab Syndr. 2017;11:251–255. doi:10.1016/j.dsx.2016.08.021.

32. Joung KH, Ju SH, Kim JM, Choung S, Lee JM, Park KS, et al. Clinical implications of using post-challenge plasma glucose levels for early diagnosis of type 2 diabetes mellitus in older individuals. Diabetes Metab J. 2018;42:147–154. doi:10.4093/dmj.2018.42.2.147.

33. Lopez-Lopez J, Garay J, Wandurraga E, Camacho PA, Higuera-Escalante F, Cohen D, et al. The simultaneous assessment of glycosylated hemoglobin, fasting plasma glucose and oral glucose tolerance test does not improve the detection of type 2 diabetes mellitus in Colombian adults. PLoS One. 2018;13:e0194446. doi:10.1371/journal.pone.0194446.

34. Prakaschandra R, Naidoo DP. Fasting plasma glucose and the HbA1c are not optimal screening modalities for the diagnosis of new diabetes in previously undiagnosed Asian Indian community participants. Ethn Dis. 2018;28:19–24. doi:10.18865/ed.28.1.19.

35. Aamir AH, Ul-Haq Z, Mahar SA, Qureshi FM, Ahmad I, Jawa A, et al. Diabetes prevalence survey of Pakistan (DPS-PAK): prevalence of type 2 diabetes mellitus and prediabetes using HbA1c: a population-based survey from Pakistan. BMJ Open. 2019;9:e025300. doi:10.1136/bmjopen-2018-025300.

36. Thewjitcharoen Y, Jones EA, Butadej S, Nakasatien S, Chotwanvirat P, Wanothayaroj E, et al. Performance of HbA1c versus oral glucose tolerance test (OGTT) as a screening tool to diagnose dysglycemic status in high-risk Thai patients. BMC Endocr Disord. 2019;19:23. doi:10.1186/s12902-019-0339-6.

37. Basit A, Fawwad A, Abdul Basit K, Waris N, Tahir B, Siddiqui IA. Glycated hemoglobin (HbA1c) as diagnostic criteria for diabetes: the optimal cut-off points values for the Pakistani population; a study from second National Diabetes Survey of Pakistan (NDSP) 2016-2017. BMJ Open Diabetes Res Care. 2020;8:e001058. doi:10.1136/bmjdrc-2019-001058.

38. Cetin EG, Demir N, Kalkan K, Ozturkmen YA, Nazif P, Yucelen SY, et al. The compatibility of hemoglobin A1c with oral glucose tolerance test and fasting plasma glucose. Sisli Etfal Hastan Tip Bul. 2020;54:351–356. doi:10.14744/SEMB.2018.97992.

39. Tucker LA. Limited agreement between classifications of diabetes and prediabetes resulting from the OGTT, hemoglobin A1c, and fasting glucose tests in 7412 U.S. adults. J Clin Med. 2020;9:2207. doi:10.3390/jcm9072207.

40. Araneta MRG, Grandinetti A, Chang HK. A1C and diabetes diagnosis among Filipino Americans, Japanese Americans, and Native Hawaiians. Diabetes Care. 2010;33:2626–2628. doi:10.2337/dc10-0958.

41. Hird TR, Pirie FJ, Esterhuizen TM, O’Leary B, McCarthy MI, Young EH, et al. Burden of diabetes and first evidence for the utility of HbA1c for diagnosis and detection of diabetes in urban black South Africans: the Durban Diabetes Study. PLoS One. 2016;11:e0161966. doi:10.1371/journal.pone.0161966.

42. Huang J, Ou HY, Karnchanasorn R, Samoa R, Chuang LM, Chiu KC, et al. Clinical implication of fasting and post-challenged plasma glucose in diagnosis of diabetes mellitus. Endocrine. 2015;48:511–518. doi:10.1007/s12020-014-0301-3.

43. Kramer CK, Araneta MRG, Barrett-Connor E. A1C and diabetes diagnosis: the Rancho Bernardo Study. Diabetes Care. 2010;33:101–103. doi:10.2337/dc09-1366.

44. Lim WY, Ma S, Heng D, Tai ES, Khoo CM, Loh TP. Screening for diabetes with HbA1c: test performance of HbA1c compared to fasting plasma glucose among Chinese, Malay and Indian community residents in Singapore. Sci Rep. 2018;8:12419. doi:10.1038/s41598-018-29998-z.

45. van ’t Riet E, Alssema M, Rijkelijkhuizen JM, Kostense PJ, Nijpels G, Dekker JM. Relationship between A1C and glucose levels in the general Dutch population: the New Hoorn Study. Diabetes Care. 2010;33:61–66. doi:10.2337/dc09-0677.

46. Lee CH, Fook-Chong S. Evaluation of fasting plasma glucose as a screening test for diabetes mellitus in Singaporean adults. Diabet Med. 1997;14:119–122.

47. Ko GT, Chan JC, Yeung VT, et al. Combined use of a fasting plasma glucose concentration and HbA1c or fructosamine predicts the likelihood of having diabetes in high-risk subjects. Diabetes Care. 1998;21:1221–1225.

48. Nitiyanant W, Ploybutr S, Sriussadaporn S, Yamwong P, Vannasaeng S. Evaluation of the new fasting plasma glucose cutpoint of 7.0 mmol/l in detection of diabetes mellitus in the Thai population. Diabetes Res Clin Pract. 1998;41:171–176. doi:10.1016/S0168-8227(98)00082-5.

49. Chang CJ, Wu JS, Lu FH, Lee HL, Yang YC, Wen MJ. Fasting plasma glucose in screening for diabetes in the Taiwanese population. Diabetes Care. 1998;21:1856–1860. doi:10.2337/diacare.21.11.1856.

50. Croxson S, Thomas P. Glucose tolerance test results reappraised using recent ADA criteria. Pract Diabetes Int. 1998;15:178–180. doi:10.1002/pdi.1960150608.

51. Puavilai G, Chanprasertyotin S, Sriphrapradaeng A. Diagnostic criteria for diabetes mellitus and other categories of glucose intolerance: 1997 criteria by the Expert Committee on the Diagnosis and Classification of Diabetes Mellitus (ADA), 1998 WHO consultation criteria, and 1985 WHO criteria. Diabetes Res Clin Pract. 1999;44:21–26. doi:10.1016/S0168-8227(99)00008-X.

52. Shaw JE, Zimmet PZ, de Courten M, Dowse GK, Chitson P, Gareeboo H, et al. Impaired fasting glucose or impaired glucose tolerance. What best predicts future diabetes in Mauritius? Diabetes Care. 1999;22:399–402. doi:10.2337/diacare.22.3.399.

53. Martín Luján F, Costa Pinel B, Donado-Mazarrón Romero A, Basora Gallisà T, Basora Gallisà J, Piñol Moreso JL. ADA criteria undervalues the impact of diabetes in a high-risk Spanish population. Aten Primaria. 2000;26:517–524. doi:10.1016/S0212-6567(00)78716-6.

54. Tai ES, Lim SC, Tan BY, Chew SK, Heng D, Tan CE. Screening for diabetes mellitus--a two-step approach in individuals with impaired fasting glucose improves detection of those at risk of complications. Diabet Med. 2000;17:771–775. doi:10.1046/j.1464-5491.2000.00382.x.

55. Hwu CM, Kwok CF, Ku BI, Lin YT, Lee YS, Hsiao LC, et al. Undiagnosed glucose intolerance encountered in clinical practice: reappraisal of the use of the oral glucose tolerance test. Zhonghua Yi Xue Za Zhi (Taipei). 2001;64:435–442.

56. Rodríguez-Morán M, Guerrero-Romero F. Fasting plasma glucose diagnostic criterion, proposed by the American Diabetes Association, has low sensitivity for diagnoses of diabetes in Mexican population. J Diabetes Complications. 2001;15:171–173. doi:10.1016/S1056-8727(01)00150-7.

57. Perry RC, Shankar RR, Fineberg N, McGill J, Baron AD. HbA1c measurement improves the detection of type 2 diabetes in high-risk individuals with nondiagnostic levels of fasting plasma glucose: the Early Diabetes Intervention Program (EDIP). Diabetes Care. 2001;24:465–471. doi:10.2337/diacare.24.3.465.

58. Gatling W, Begley J. Diagnosing diabetes mellitus in clinical practice: is fasting plasma glucose a good initial test? Pract Diabetes Int. 2001;18:89–93.

59. Drzewoski J, Czupryniak L. Concordance between fasting and 2-h post-glucose challenge criteria for the diagnosis of diabetes mellitus and glucose intolerance in high risk individuals. Diabet Med. 2001;18:29–31. doi:10.1046/j.1464-5491.2001.00403.x.

60. Nakagami T, Qiao Q, Tuomilehto J, Balkau B, Carstensen B, Tajima N, et al. The fasting plasma glucose cut-point predicting a diabetic 2-h OGTT glucose level depends on the phenotype. Diabetes Res Clin Pract. 2002;55:35–43. doi:10.1016/S0168-8227(01)00270-4.

61. Mannucci E, Ognibene A, Sposato I, Brogi M, Gallori G, Bardini G, et al. Fasting plasma glucose and glycated haemoglobin in the screening of diabetes and impaired glucose tolerance. Acta Diabetol. 2003;40:181–186. doi:10.1007/s00592-003-0109-8.

62. Daniel M, Rowley KG, Marks E, O'Dea K. Test agreement for classifying diabetes in indigenous Australians. Aust N Z J Public Health. 2006;30:128–131. doi:10.1111/j.1467-842X.2006.tb00104.x.

63. Shrestha UK, Singh DL, Bhattarai MD. The prevalence of hypertension and diabetes defined by fasting and 2-h plasma glucose criteria in urban Nepal. Diabet Med. 2006;23:1130–1135. doi:10.1111/j.1464-5491.2006.01953.x.

64. Soma P, Rheeder P. Unsuspected glucose abnormalities in patients with coronary artery disease. S Afr Med J. 2006;96:216–220.

65. Al-Lawati JA, Barakat MN. Fasting cut-points in determining prevalence of diabetes in an Arab population of the Middle East. Diabetes Res Clin Pract. 2007;75:241–245. doi:10.1016/j.diabres.2006.06.018.

66. Gao W, Dong Y, Nan H, Tuomilehto J, Qiao Q. The likelihood of diabetes based on the proposed definitions for impaired fasting glucose. Diabetes Res Clin Pract. 2008;79:151–155. doi:10.1016/j.diabres.2007.07.018.

67. Koike Y, Ogushi Y, Jin D, Sato H, Yamada T. Fasting plasma glucose reference values among young Japanese women requiring 75 g oral glucose tolerance tests. Tokai J Exp Clin Med. 2009;34:15–20.

68. Hofsø D, Jenssen T, Hager H, Røislien J, Hjelmesaeth J. Fasting plasma glucose in the screening for type 2 diabetes in morbidly obese subjects. Obes Surg. 2010;20:302–307. doi:10.1007/s11695-009-0022-5.

69. Wen ZZ, Zhang XM, Mai Z, Geng DF, Wang JF. Predictive value of first fasting plasma glucose compared with admission plasma glucose for undiagnosed diabetes in a stable cardiology population. Clin Biochem. 2012;45:1057–1063. doi:10.1016/j.clinbiochem.2012.05.020.

70. Huang H, Peng G, Lin M, Zhang K, Wang Y, Yang Y, et al. The diagnostic threshold of HbA1c and impact of its use on diabetes prevalence: a population-based survey of 6898 Han participants from southern China. Prev Med. 2013;57:345–350. doi:10.1016/j.ypmed.2013.06.012.

71. Aekplakorn W, Tantayotai V, Numsangkul S, Sripho W, Tatsato N, Burapasiriwat T, et al. Detecting prediabetes and diabetes: agreement between fasting plasma glucose and oral glucose tolerance test in Thai adults. J Diabetes Res. 2015;2015:396505. doi:10.1155/2015/396505.

72. Kim DL, Kim SD, Kim SK, Park S, Song KH. Is an oral glucose tolerance test still valid for diagnosing diabetes mellitus? Diabetes Metab J. 2016;40:118–128. doi:10.4093/dmj.2016.40.2.118.

73. Kengne AP, Erasmus RT, Levitt NS, Matsha TE. Alternative indices of glucose homeostasis as biochemical diagnostic tests for abnormal glucose tolerance in an African setting. Prim Care Diabetes. 2017;11:119–131. doi:10.1016/j.pcd.2017.01.004.

74. Katulanda GW, Katulanda P, Dematapitiya C, Dissanayake HA, Wijeratne S, Sheriff MHR, et al. Plasma glucose in screening for diabetes and pre-diabetes: how much is too much? Analysis of fasting plasma glucose and oral glucose tolerance test in Sri Lankans. BMC Endocr Disord. 2019;19:11. doi:10.1186/s12902-019-0343-x.

75. Chume FC, Kieling MH, Correa Freitas PA, Cavagnolli G, Camargo JL. Glycated albumin as a diagnostic tool in diabetes: an alternative or an additional test? PLoS One. 2019;14:e0227065. doi:10.1371/journal.pone.0227065.

76. Zemlin AE, Barkhuizen M, Kengne AP, Erasmus RT, Matsha TE. Performance of glycated albumin for type 2 diabetes and prediabetes diagnosis in a South African population. Clin Chim Acta. 2019;488:122–128. doi:10.1016/j.cca.2018.11.005.

77. Wu WC, Ma WY, Wei JN, Yu TY, Lin MS, Shih SR, et al. Serum glycated albumin to guide the diagnosis of diabetes mellitus. PLoS One. 2016;11:e0146780. doi:10.1371/journal.pone.0146780.

78. Ikezaki H, Furusyo N, Ihara T, Hayashi T, Ura K, Hiramine S, et al. Glycated albumin as a diagnostic tool for diabetes in a general Japanese population. Metabolism. 2015;64:698–705. doi:10.1016/j.metabol.2015.03.003.

**Supplementary Table S1.** Detailed results of the risk-of-bias assessment.

| Author | Year |  | Risk of bias | | | | | | |  | Concerns of applicability | | | | |
| --- | --- | --- | --- | --- | --- | --- | --- | --- | --- | --- | --- | --- | --- | --- | --- |
|  |  |  | Patient selection |  | Index test |  | Reference standard |  | Flow and timing |  | Patient selection |  | Index test |  | Reference standard |
| **1-hPG** |  |  |  |  |  |  |  |  |  |  |  |  |  |  |  |
| Pyörälä | 1966 |  | Low |  | Low |  | Low |  | Low |  | Low |  | Low |  | Low |
| Paddock | 1966 |  | Low |  | Low |  | Low |  | Low |  | Low |  | Low |  | Low |
| Tripathy | 1990 |  | Low |  | Low |  | Low |  | Low |  | Low |  | Low |  | Low |
| Bergman | 1979 |  | Low |  | Low |  | Low |  | Low |  | Low |  | Low |  | Low |
| Sai Prasanna | 1991 |  | Low |  | Low |  | Low |  | Low |  | Low |  | Low |  | Low |
| Abdul-Ghani | 1992 |  | Low |  | Low |  | Low |  | Low |  | Low |  | Low |  | Low |
| Abdul-Ghani | 1996 |  | Low |  | Low |  | Low |  | Low |  | Low |  | Low |  | Low |
| Mutt | 2001 |  | Low |  | Low |  | Low |  | Low |  | Low |  | Low |  | Low |
| Bianchi | 2003 |  | High |  | Low |  | Low |  | Low |  | Low |  | Low |  | Low |
| Succurro | 2005 |  | High |  | Low |  | Low |  | Low |  | Low |  | Low |  | Low |
| Oka | 2006 |  | Low |  | Low |  | Low |  | Low |  | Low |  | Low |  | Low |
| La Sala | 2014 |  | Low |  | Low |  | Low |  | Low |  | Low |  | Low |  | Low |
| Kim | 2017 |  | Unclear |  | Low |  | Unclear |  | Unclear |  | Low |  | Low |  | Low |
| Ferrannini | 2022 |  | Unclear |  | Low |  | Low |  | Low |  | High |  | Low |  | Low |
| Castro | 2025 |  | Unclear |  | Low |  | Unclear |  | Unclear |  | High |  | Low |  | Low |
| Thenmozhi | 2025 |  | Unclear |  | Unclear |  | Low |  | Low |  | Low |  | Low |  | Low |
| **HbA1c** |  |  |  |  |  |  |  |  |  |  |  |  |  |  |  |
| Wiener | 1998 |  | Low |  | Low |  | Low |  | Low |  | Low |  | Low |  | Low |
| Tanaka | 2001 |  | Low |  | Unclear |  | Unclear |  | Unclear |  | Low |  | Low |  | Low |
| Kramer | 2010 |  | Unclear |  | Low |  | Low |  | Unclear |  | Low |  | Low |  | Low |
| van't Riet | 2010 |  | Unclear |  | Unclear |  | Low |  | Low |  | Low |  | Low |  | Low |
| Araneta | 2010 |  | Unclear |  | Low |  | Low |  | Unclear |  | Low |  | Low |  | Low |
| Zemlin | 2011 |  | Low |  | Low |  | Low |  | Low |  | Low |  | Low |  | Low |
| Adamu | 2011 |  | Low |  | Low |  | Low |  | Low |  | Low |  | Low |  | Low |
| Valentine | 2011 |  | High |  | Low |  | Low |  | High |  | Low |  | Low |  | Low |
| Adamska | 2012 |  | Low |  | Low |  | Low |  | Low |  | Low |  | Low |  | Low |
| Marini | 2012 |  | Low |  | Low |  | Low |  | High |  | Low |  | Low |  | Low |
| Homko | 2012 |  | Low |  | Low |  | Low |  | Low |  | Low |  | Low |  | Low |
| Lee | 2013 |  | Low |  | Low |  | Low |  | Unclear |  | Low |  | Low |  | Low |
| Alqahtani | 2013 |  | Low |  | Low |  | Low |  | Unclear |  | Low |  | Low |  | Low |
| Huang | 2013 |  | Unclear |  | Low |  | Low |  | Unclear |  | Low |  | Low |  | Low |
| Franco | 2014 |  | High |  | Low |  | Low |  | Low |  | High |  | Low |  | Low |
| Aviles-Santa | 2016 |  | Low |  | Low |  | Low |  | Unclear |  | Low |  | Low |  | Low |
| Karnchanasorn | 2016 |  | Low |  | Low |  | Low |  | Low |  | Low |  | Low |  | Low |
| Camacho | 2016 |  | High |  | Low |  | Low |  | Low |  | Low |  | Low |  | Low |
| Hird | 2016 |  | Low |  | Low |  | Low |  | Low |  | Low |  | Low |  | Low |
| Herath | 2017 |  | Low |  | Low |  | Low |  | Low |  | Low |  | Low |  | Low |
| Kim | 2017 |  | Unclear |  | Low |  | Unclear |  | Unclear |  | Low |  | Low |  | Low |
| Lopez | 2018 |  | Low |  | Low |  | Low |  | Low |  | Low |  | Low |  | Low |
| Joung | 2018 |  | Low |  | Low |  | Low |  | Low |  | Low |  | Low |  | Low |
| Prakashchandra | 2018 |  | Low |  | Low |  | Low |  | Unclear |  | Low |  | Low |  | Low |
| Lim | 2018 |  | Low |  | Low |  | Low |  | Low |  | Low |  | Low |  | Low |
| Aamir | 2019 |  | Low |  | Low |  | Low |  | High |  | Low |  | Low |  | Low |
| Thewjitcharoen | 2019 |  | Low |  | Low |  | High |  | High |  | Low |  | Low |  | Low |
| Basit | 2020 |  | Low |  | Low |  | Low |  | High |  | Low |  | Low |  | Low |
| Cetin | 2020 |  | Low |  | Low |  | Low |  | Low |  | Low |  | Low |  | Low |
| Tucker | 2020 |  | Low |  | Low |  | Low |  | Low |  | Low |  | Low |  | Low |
| **FPG** |  |  |  |  |  |  |  |  |  |  |  |  |  |  |  |
| Lee | 1997 |  | Unclear |  | Low |  | Low |  | Low |  | Low |  | Low |  | Low |
| Chang | 1998 |  | Low |  | Low |  | Low |  | Low |  | Low |  | Low |  | Low |
| Croxson | 1998 |  | Low |  | Low |  | Low |  | Low |  | Low |  | Low |  | Low |
| Ko | 1998 |  | Unclear |  | Low |  | Low |  | Low |  | Low |  | Low |  | Low |
| Nitiyanant | 1998 |  | Low |  | Low |  | Low |  | Low |  | Low |  | Low |  | Low |
| Wiener | 1998 |  | Low |  | Low |  | Low |  | Low |  | Low |  | Low |  | Low |
| Shaw | 1999 |  | Low |  | Low |  | Low |  | Low |  | Low |  | Low |  | Low |
| Puavilai | 1999 |  | Low |  | Low |  | Low |  | Low |  | Low |  | Low |  | Low |
| Tai | 2000 |  | Low |  | Low |  | Low |  | Low |  | Low |  | Low |  | Low |
| Lujan | 2000 |  | Low |  | Low |  | Low |  | Low |  | Low |  | Low |  | Low |
| Gatling | 2001 |  | High |  | Low |  | High |  | High |  | Low |  | Low |  | Low |
| Drzewoski | 2001 |  | High |  | Low |  | Low |  | Low |  | Low |  | Low |  | Low |
| Perry | 2001 |  | High |  | Low |  | High |  | High |  | High |  | Low |  | Low |
| Tanaka | 2001 |  | Low |  | Unclear |  | Unclear |  | Unclear |  | Low |  | Low |  | Low |
| Moran | 2001 |  | Low |  | Low |  | Low |  | Low |  | Low |  | Low |  | Low |
| Hwu | 2001 |  | Low |  | Low |  | Low |  | Low |  | Low |  | Low |  | Low |
| Nakagami | 2002 |  | Low |  | Low |  | Low |  | Unclear |  | Low |  | Low |  | Low |
| Mannucci | 2003 |  | Low |  | Low |  | Low |  | Low |  | Low |  | Low |  | Low |
| Shrestha | 2006 |  | Low |  | Low |  | Low |  | Low |  | Low |  | Low |  | Low |
| Daniel | 2006 |  | Low |  | Unclear |  | Unclear |  | Unclear |  | High |  | Low |  | Low |
| Soma | 2006 |  | Low |  | Low |  | Low |  | Low |  | High |  | Low |  | Low |
| Al-Lawati | 2007 |  | Low |  | Low |  | Low |  | Low |  | Low |  | Low |  | Low |
| Gao | 2008 |  | Low |  | Low |  | Low |  | High |  | Low |  | Low |  | Low |
| Koike | 2009 |  | Unclear |  | Low |  | Low |  | High |  | Low |  | Low |  | Low |
| Hofso | 2010 |  | Low |  | Low |  | Low |  | High |  | Low |  | Low |  | Low |
| Adamu | 2011 |  | Low |  | Low |  | Low |  | Low |  | Low |  | Low |  | Low |
| Wen | 2012 |  | Low |  | Low |  | Low |  | Low |  | High |  | Low |  | Low |
| Homko | 2012 |  | Low |  | Low |  | Low |  | Low |  | Low |  | Low |  | Low |
| Lee | 2013 |  | Low |  | Low |  | Low |  | Unclear |  | Low |  | Low |  | Low |
| Aekplakorn | 2015 |  | Low |  | Low |  | Low |  | Low |  | High |  | Low |  | Low |
| Huang | 2015 |  | Low |  | Low |  | Low |  | High |  | Low |  | Low |  | Low |
| Aviles-Santa | 2016 |  | Low |  | Low |  | Low |  | Unclear |  | Low |  | Low |  | Low |
| Kim | 2016 |  | High |  | Low |  | Low |  | Low |  | Low |  | Low |  | Low |
| Kenge | 2017 |  | High |  | Low |  | Low |  | High |  | Low |  | Low |  | Low |
| Herath | 2017 |  | Low |  | Low |  | Low |  | Low |  | Low |  | Low |  | Low |
| Kim | 2017 |  | Unclear |  | Low |  | Unclear |  | Unclear |  | Low |  | Low |  | Low |
| Lopez | 2018 |  | Low |  | Low |  | Low |  | Low |  | Low |  | Low |  | Low |
| Joung | 2018 |  | Low |  | Low |  | Low |  | Low |  | Low |  | Low |  | Low |
| Prakashchandra | 2018 |  | Low |  | Low |  | Low |  | Unclear |  | Low |  | Low |  | Low |
| Katulanda | 2019 |  | Low |  | Low |  | Low |  | High |  | Low |  | Low |  | Low |
| Tucker | 2020 |  | Low |  | Low |  | Low |  | Low |  | Low |  | Low |  | Low |
| Cetin | 2020 |  | Low |  | Low |  | Low |  | Low |  | Low |  | Low |  | Low |
| **GA** |  |  |  |  |  |  |  |  |  |  |  |  |  |  |  |
| Ikezaki | 2015 |  | High |  | Low |  | Low |  | High |  | High |  | Low |  | Low |
| Wu | 2016 |  | Low |  | Low |  | Low |  | Low |  | Low |  | Low |  | Low |
| Zemlin | 2019 |  | Low |  | Low |  | Low |  | Low |  | Low |  | Low |  | Low |
| Chume | 2019 |  | Low |  | Low |  | Low |  | Low |  | Low |  | Low |  | Low |
| **HbA1c or FPG** |  |  |  |  |  |  |  |  |  |  |  |  |  |  |  |
| Tanaka | 2001 |  | Low |  | Unclear |  | Unclear |  | Unclear |  | Low |  | Low |  | Low |
| Lee | 2013 |  | Low |  | Low |  | Low |  | Unclear |  | Low |  | Low |  | Low |
| Aviles-Santa | 2016 |  | Low |  | Low |  | Low |  | Unclear |  | Low |  | Low |  | Low |
| Herath | 2017 |  | Low |  | Low |  | Low |  | Low |  | Low |  | Low |  | Low |
| Kim | 2017 |  | Unclear |  | Low |  | Unclear |  | Unclear |  | Low |  | Low |  | Low |
| Lopez | 2018 |  | Low |  | Low |  | Low |  | Low |  | Low |  | Low |  | Low |
| Joung | 2018 |  | Low |  | Low |  | Low |  | Low |  | Low |  | Low |  | Low |

**Abbreviations:** 1-h PG, 1-hour plasma glucose;HbA1c, glycated hemoglobin; FPG, fasting plasma glucose; GA, glycated albumin

**Supplementary Table S2.** Detailed results of the comparisons under the ANOVA model.

| Tests | SEN | SPE | DOR | Superiority Index | RSEN | RSPE |
| --- | --- | --- | --- | --- | --- | --- |
| 1 h PG | 0.87  (95%(CrI): 0.82 – 0.91) | 0.88  (95%CrI: 0.82 – 0.92) | 52.44  (95%CrI: 30.17 – 84.00) | 3.22  (95%CrI: 1.00 – 7.00) | 6.47  (95%CrI: 4.03 – 9.73) | 0.34(95%CrI: 0.18 – 0.59) |
| HbA1c | 0.53  (95%CrI: 0.47 – 0.58) | 0.92  (95%CrI: 0.89 – 0.94) | 13.15  (95%CrI: 8.94 – 18.51) | 1.17  (95%CrI:0.20 – 3.00) | 1.06  (95%CrI:0.82 – 1.36) | 0.51(95%CrI:0.32 – 0.78) |
| FPG | 0.51  (95%CrI: 0.47 – 0.55) | 0.96  (95%CrI: 0.95 – 0.97) | 24.94  (95%CrI: 17.90 – 32.30) | 2.56  (95%CrI: 1.00 – 5.00) | 1 | 1 |
| GA | 0.53  (95%CrI: 0.36 – 0.71) | 0.86  (95%CrI: 0.71 – 0.95) | 8.53  (95%CrI: 2.70 – 20.94) | 0.44  (95%CrI: 0.11 – 3.00) | 1.15  (95%CrI: 0.50 – 2.25) | 0.32(95%CrI: 0.10 – 0.81) |
| HbA1c or FPG | 0.64  (95% CrI: 0.54 – 0.73) | 0.89  (95%CrI: 0.81 – 0.95) | 15.83  (95%CrI: 6.62 – 31.47) | 1.89  (95%CrI: 0.20 – 5.00) | 1.70  (95%CrI: 1.11 – 2.52) | 0.38(95%CrI:0.17 – 0.75) |

**Abbreviations:** 1-h PG, 1-hour plasma glucose; OGTT, oral glucose tolerance test; HbA1c, glycated hemoglobin; FPG, fasting plasma glucose; GA, glycated albumin; SEN, sensitivity; SPE, specificity; DOR, diagnostic odds ratio; RSEN, relative sensitivity; RSPE, relative specificity.


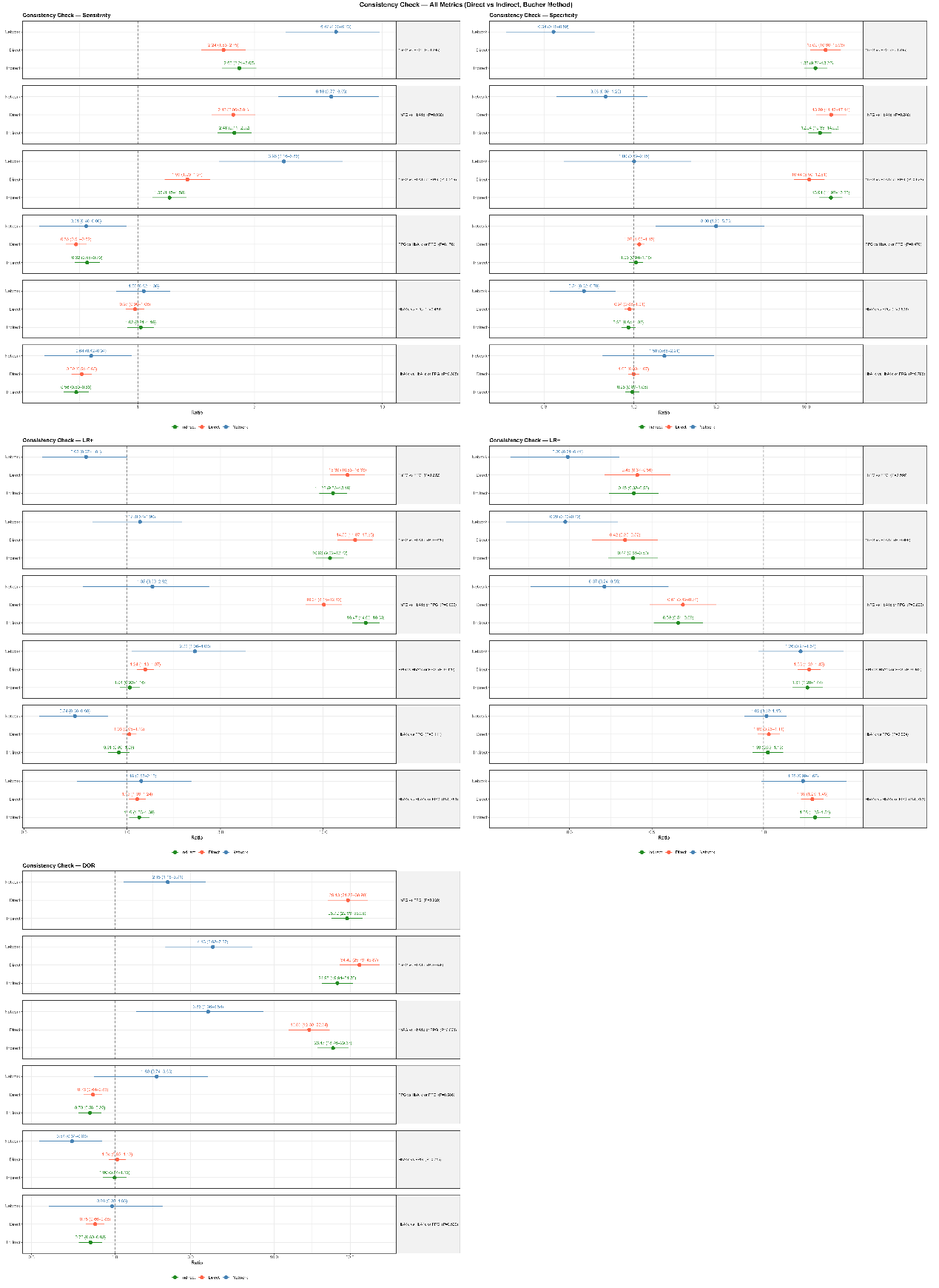
**Supplementary Figure 1.** Node-splitting results for sensitivity, specificity, LR+, LR−, and DOR across the five diagnostic strategies. LR+, positive likelihood ratio; LR−, negative likelihood ratio; DOR, diagnostic odds ratio.

1-h PG, 1-hour plasma glucose; HbA1c, glycated hemoglobin; FPG, fasting plasma glucose; GA, glycated albumin.


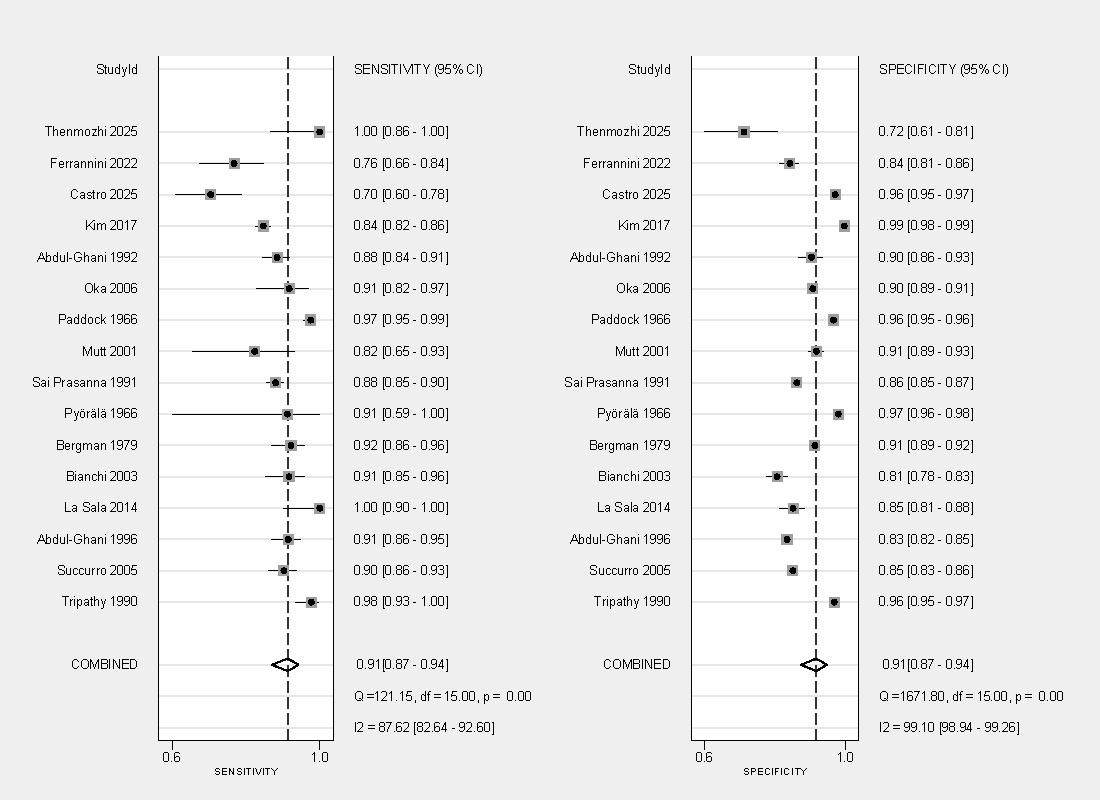


**Supplementary Figure 2A.** Forest plots of pooled sensitivity and specificity for 1-h PG in the pairwise meta-analysis.


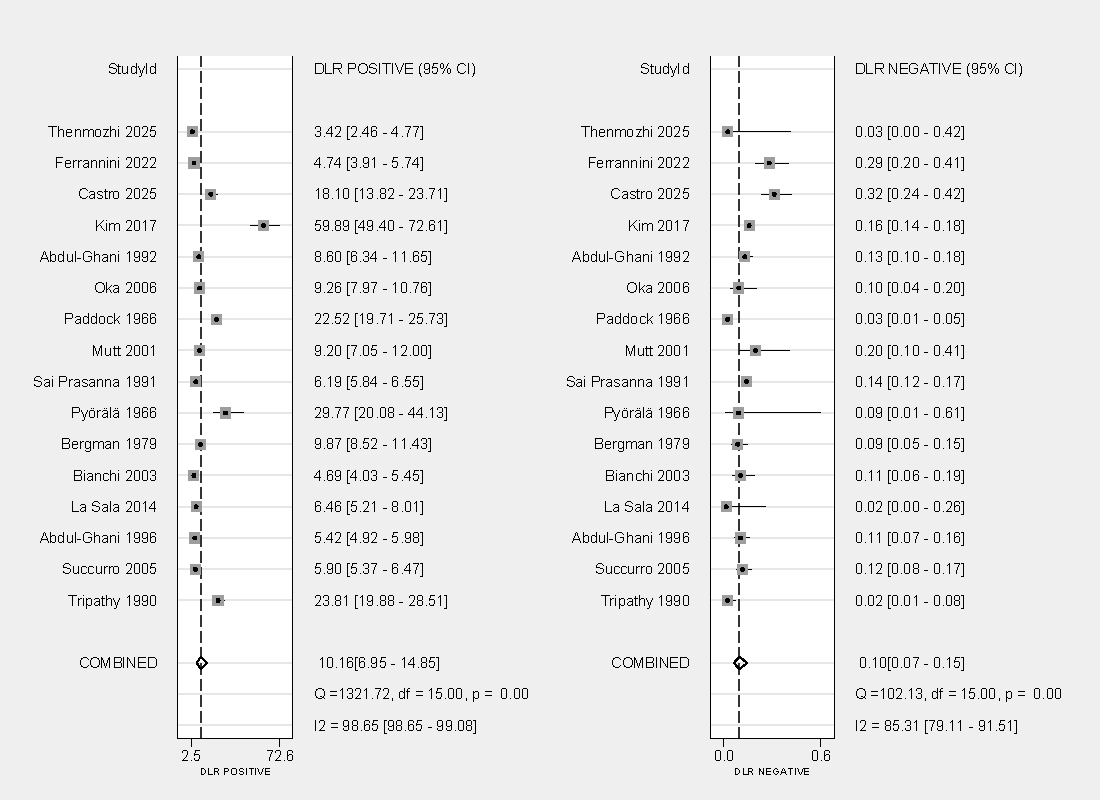
 **Supplementary Figure 3A.** Forest plots of pooled positive likelihood ratio (LR+) and negative likelihood ratio (LR−) for 1-h PG in the pairwise meta-analysis.


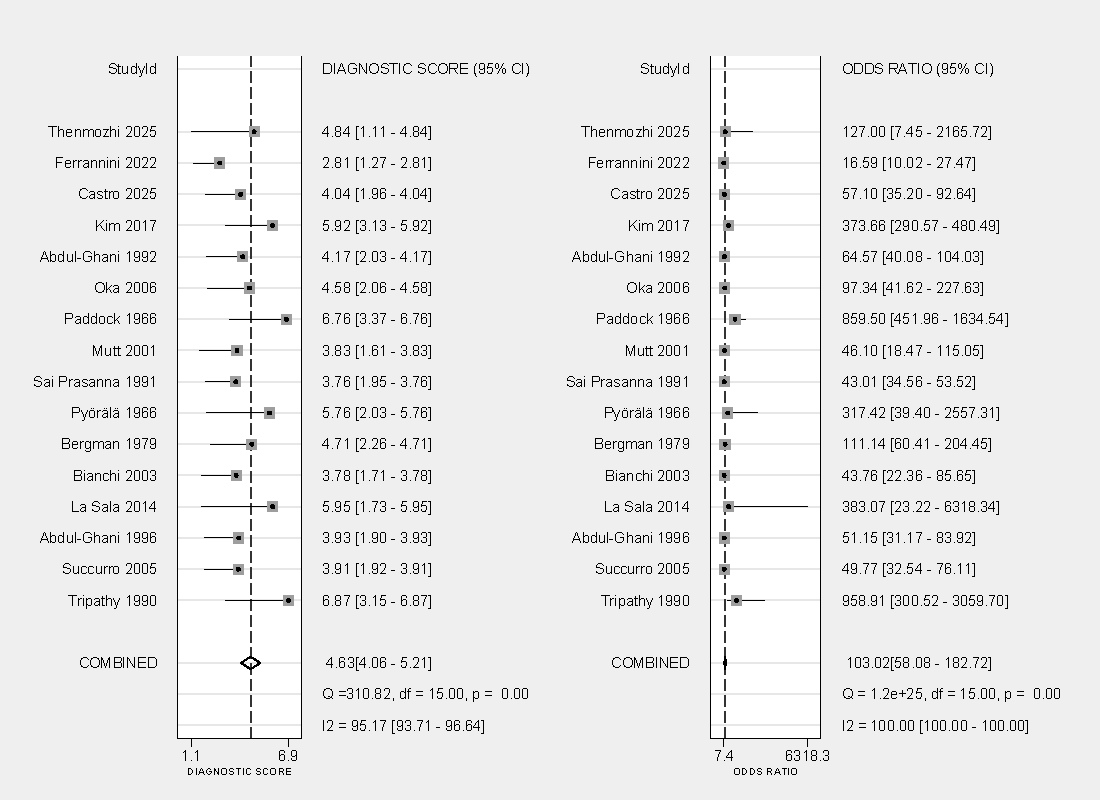


**Supplementary Figure 4A.** Forest plot of pooled diagnostic odds ratio (DOR) for 1-h PG in the pairwise meta-analysis.


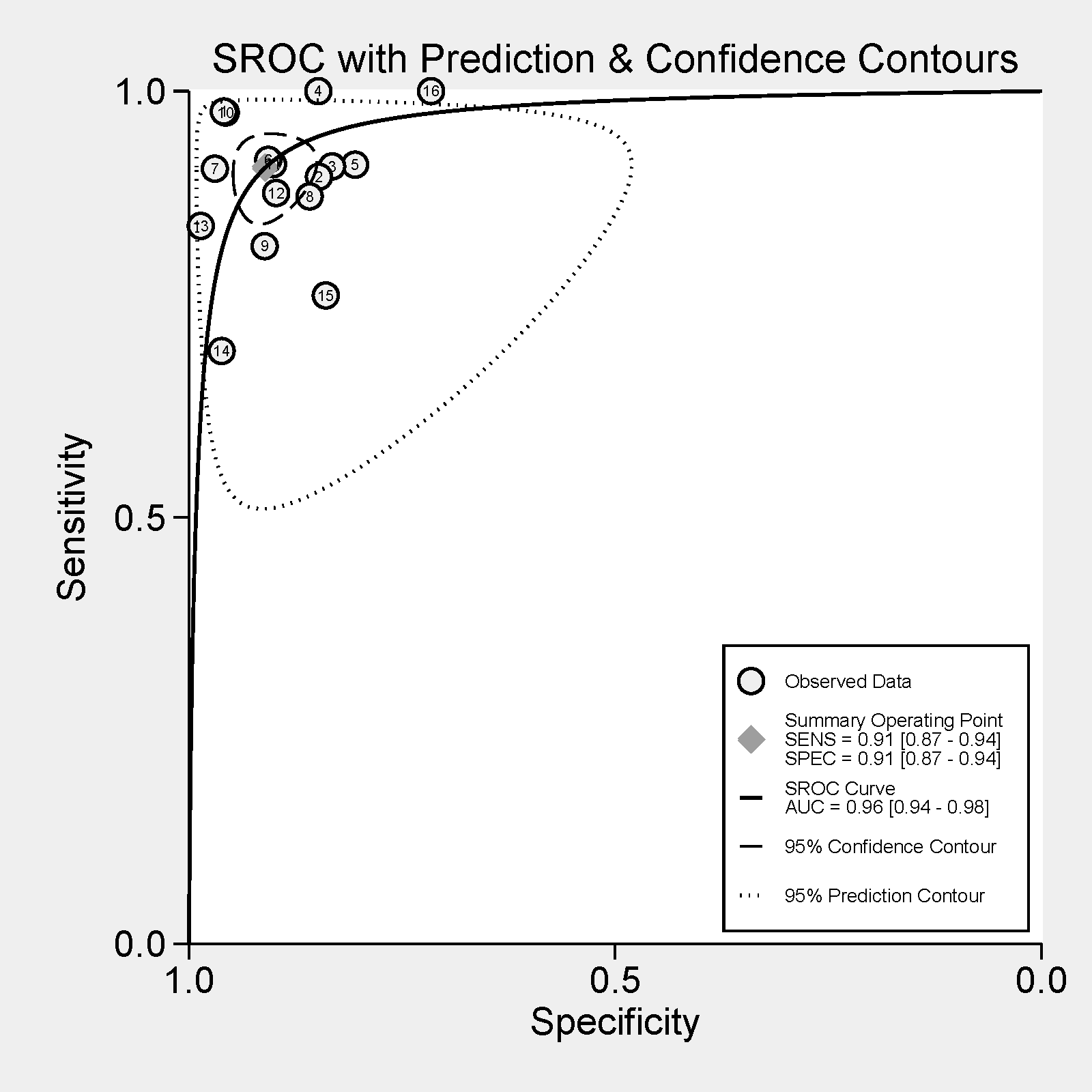


**Supplementary Figure 5A.** Summary receiver operating characteristic (SROC) curve for 1-h PG in the pairwise meta-analysis.


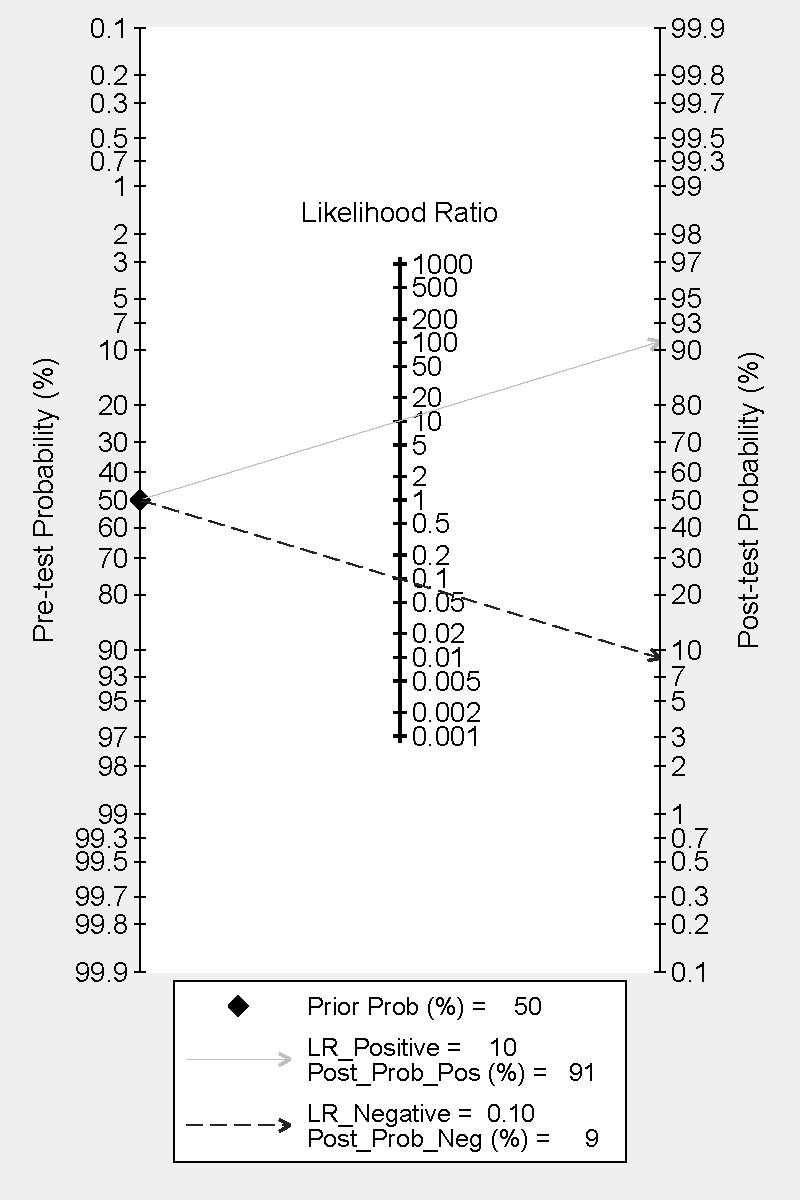


**Supplementary Figure 6A.** Fagan nomogram for 1-h PG in the pairwise meta-analysis, showing its potential clinical utility for diagnosing T2DM. Based on the pooled positive and negative likelihood ratios, a positive 1-h PG result markedly increased the post-test probability of T2DM, whereas a negative result substantially reduced the post-test probability, indicating that 1-h PG may be clinically useful for both confirming and excluding T2DM in appropriate screening settings.


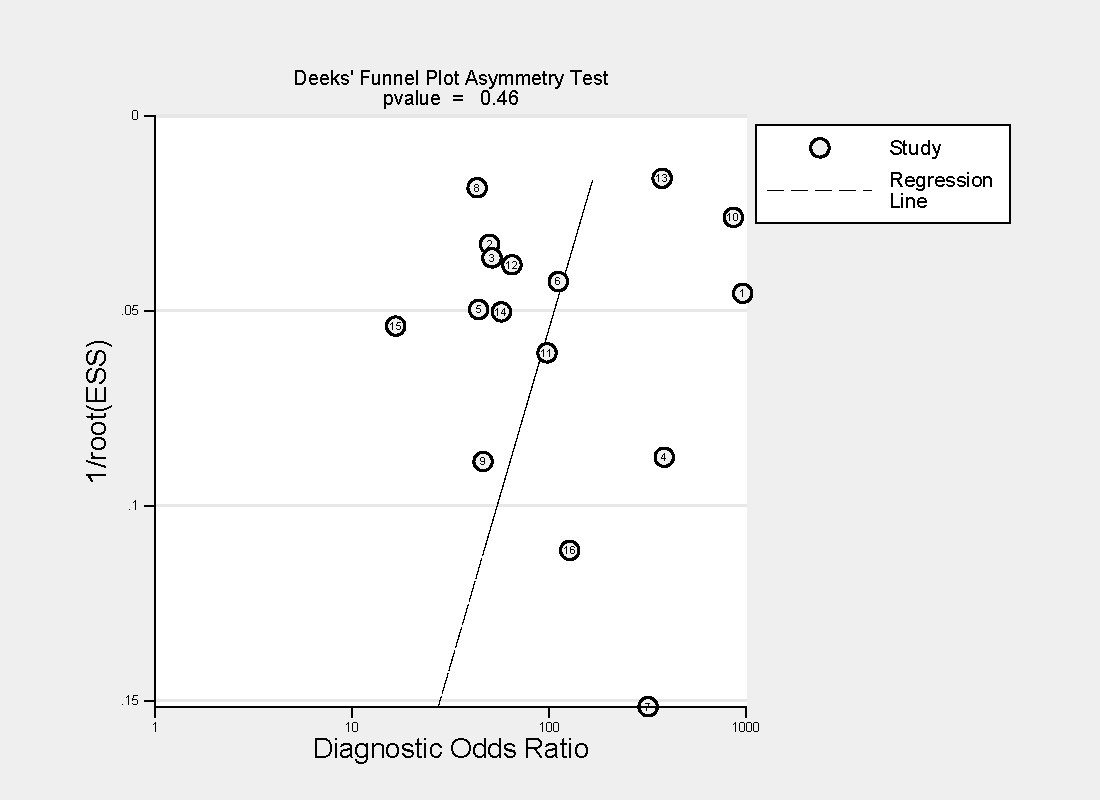
 **Supplementary Figure 7A.** Deeks’ funnel plot for publication bias assessment of 1-h PG in the pairwise meta-analysis.


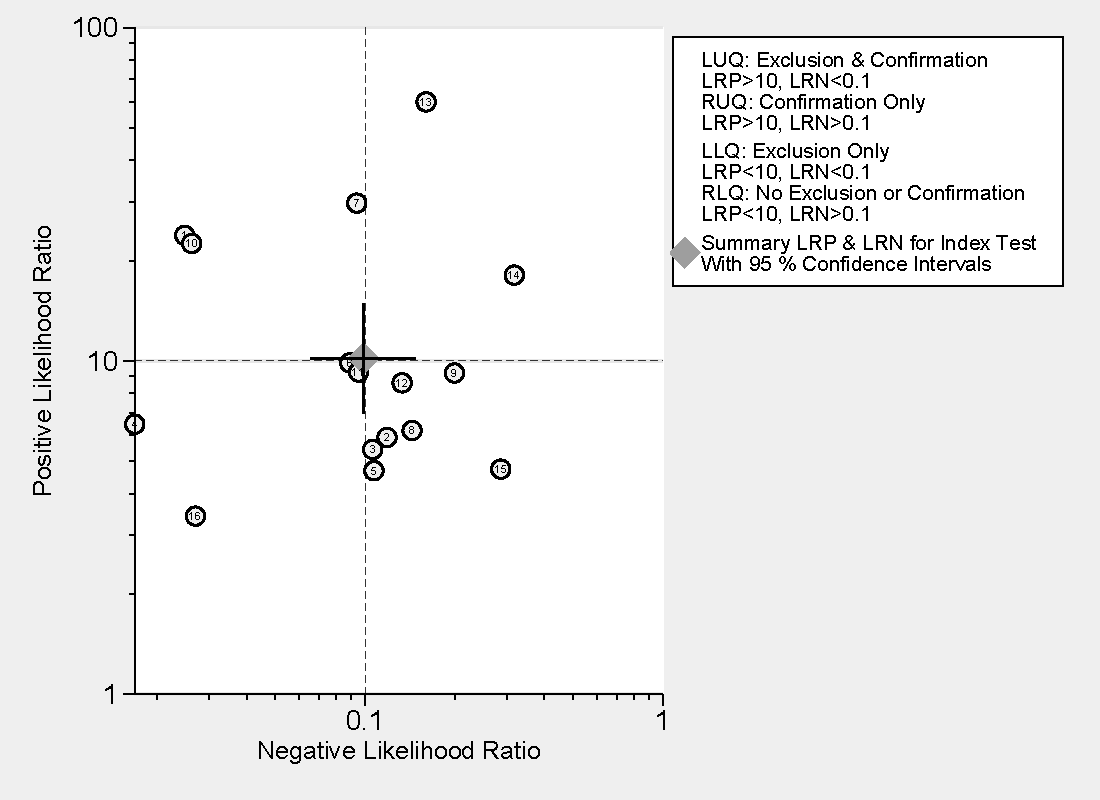
 **Supplementary Figure 8A.** Likelihood-ratio scatter plot for 1-h PG in the pairwise meta-analysis, showing that 1-h PG has both rule-in and rule-out value for T2DM. The position of the pooled estimates in the likelihood-ratio scatter plot indicates that 1-h PG provides meaningful information for both confirming and excluding T2DM, suggesting favorable overall diagnostic performance as a stand-alone screening test.


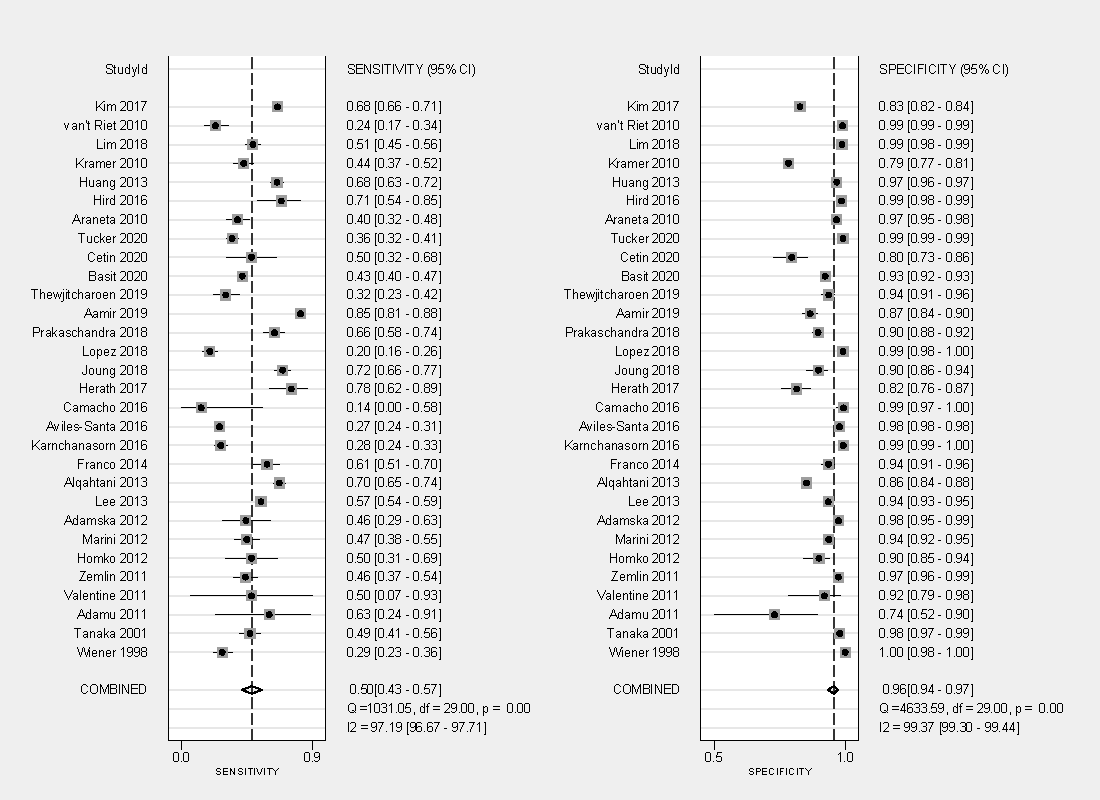
 **Supplementary Figure 2B.** Forest plots of pooled sensitivity and specificity for HbA1c in the pairwise meta-analysis.


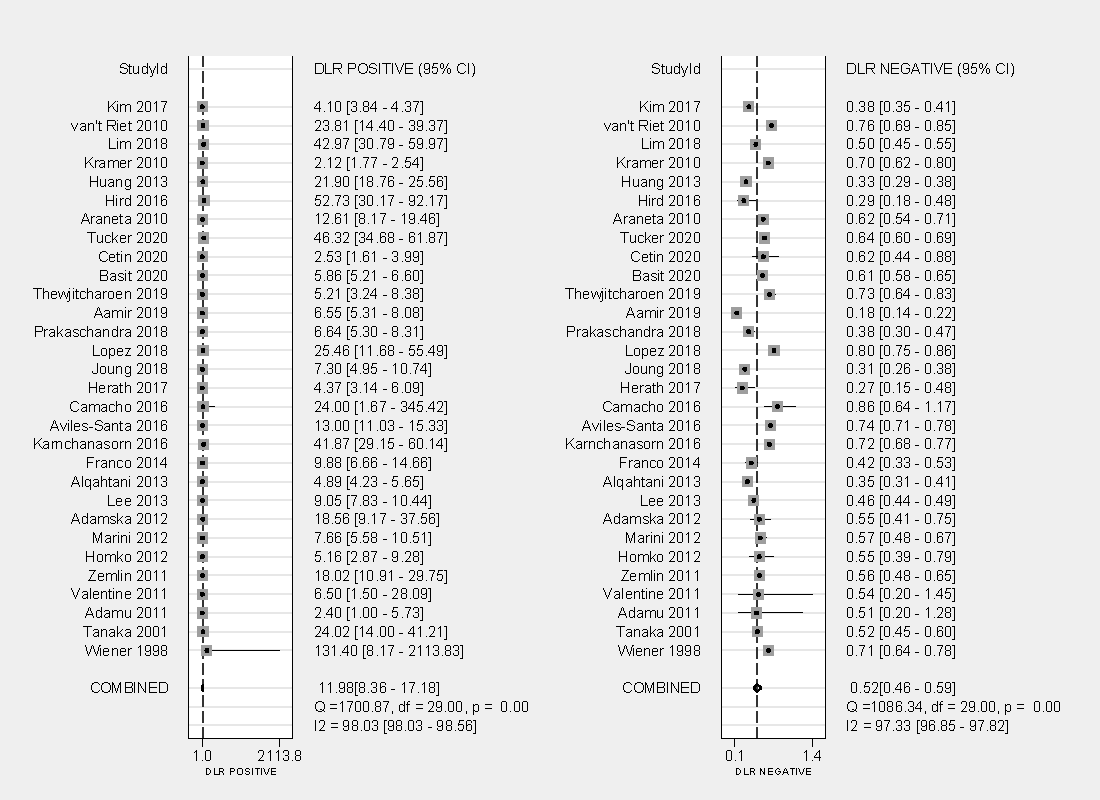


**Supplementary Figure 3B.** Forest plots of pooled positive likelihood ratio (LR+) and negative likelihood ratio (LR−) for HbA1c in the pairwise meta-analysis.


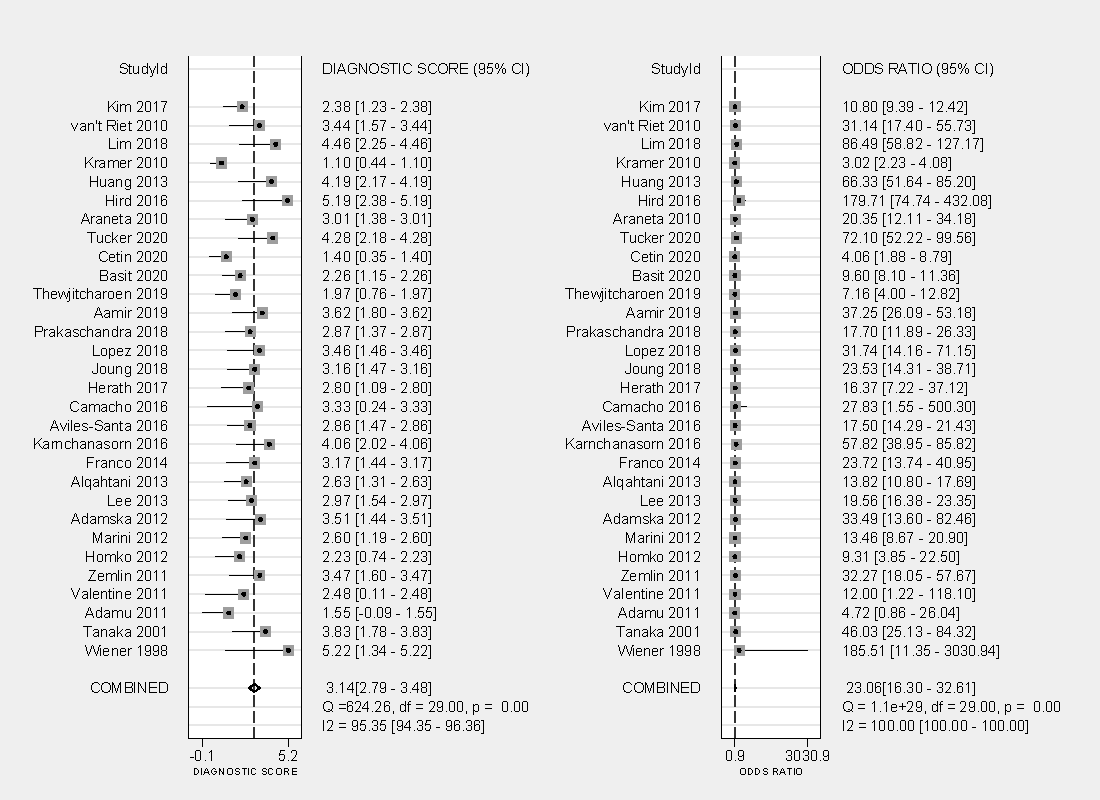


**Supplementary Figure 4B.** Forest plot of pooled diagnostic odds ratio (DOR) for HbA1c in the pairwise meta-analysis.


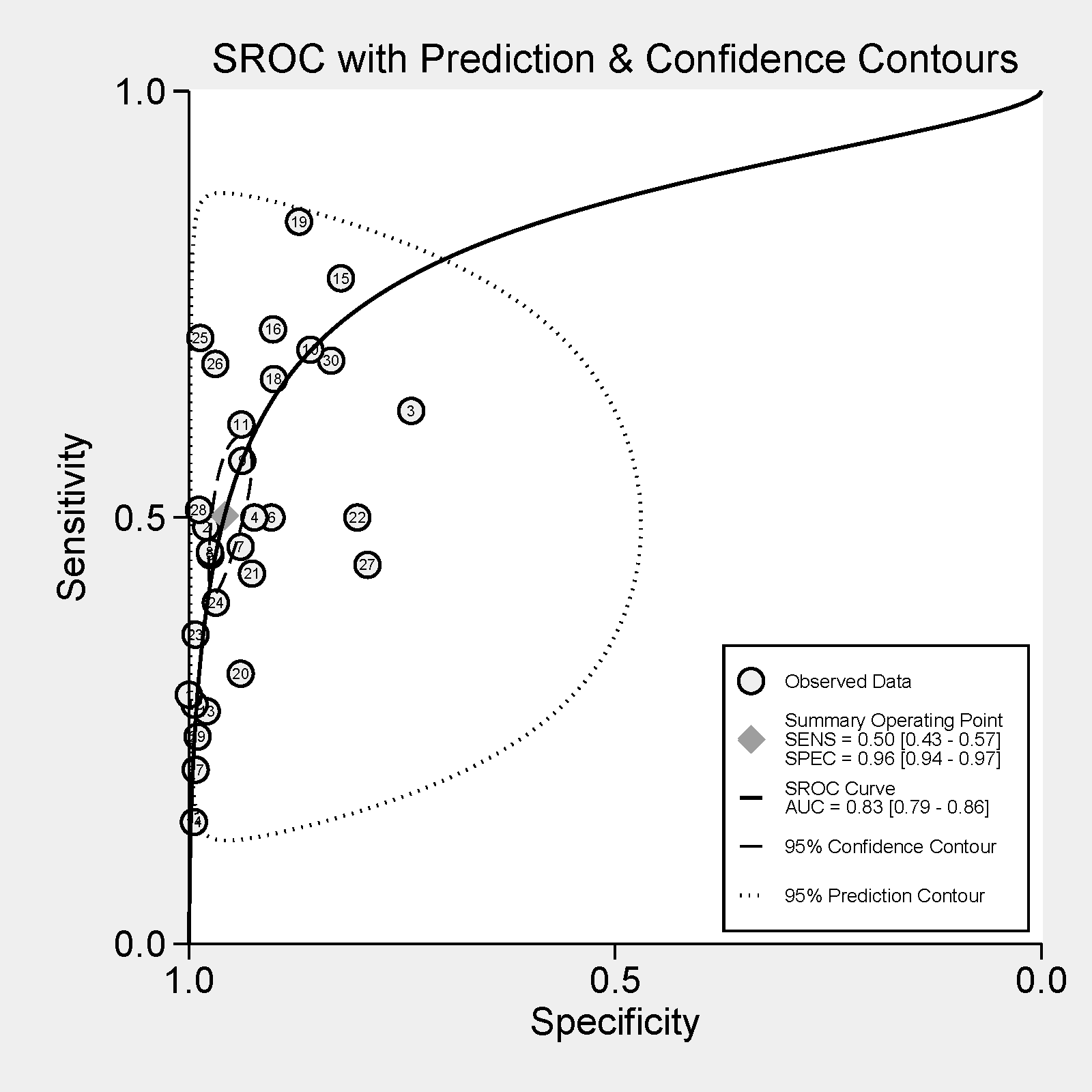


**Supplementary Figure 5B.** Summary receiver operating characteristic (SROC) curve for HbA1c in the pairwise meta-analysis.


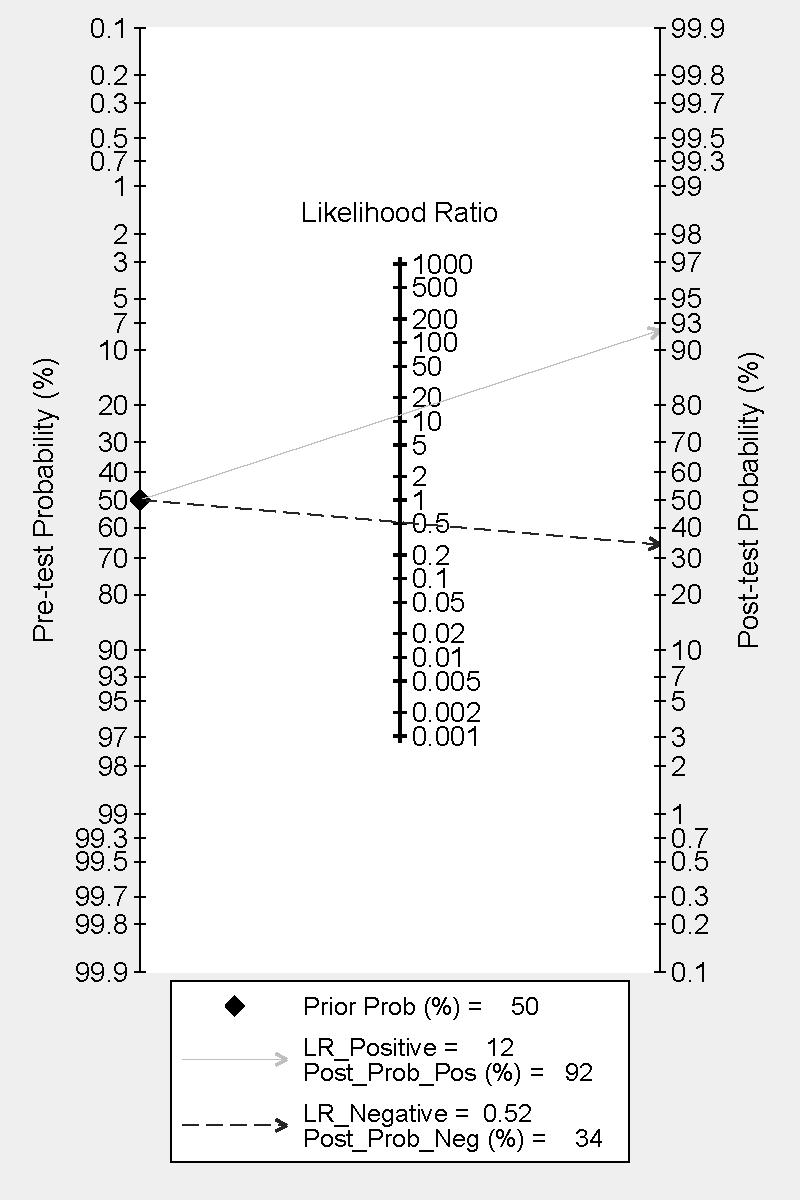


**Supplementary Figure 6B.** Fagan nomogram for HbA1c in the pairwise meta-analysis, showing its potential clinical utility for diagnosing T2DM. Based on the pooled positive and negative likelihood ratios, a positive HbA1c result markedly increased the post-test probability of T2DM, whereas a negative result substantially reduced the post-test probability, indicating that HbA1c may be clinically useful for both confirming and excluding T2DM in appropriate screening settings.


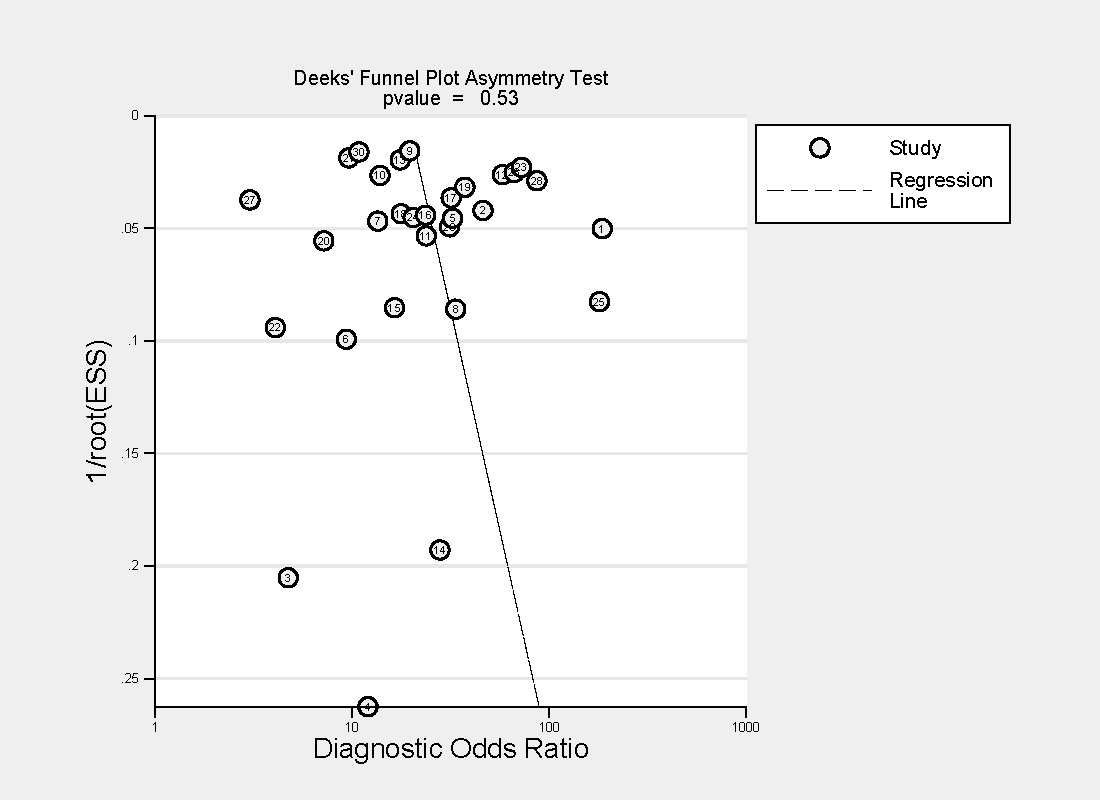
 **Supplementary Figure 7B.** Deeks’ funnel plot for publication bias assessment of HbA1c in the pairwise meta-analysis.


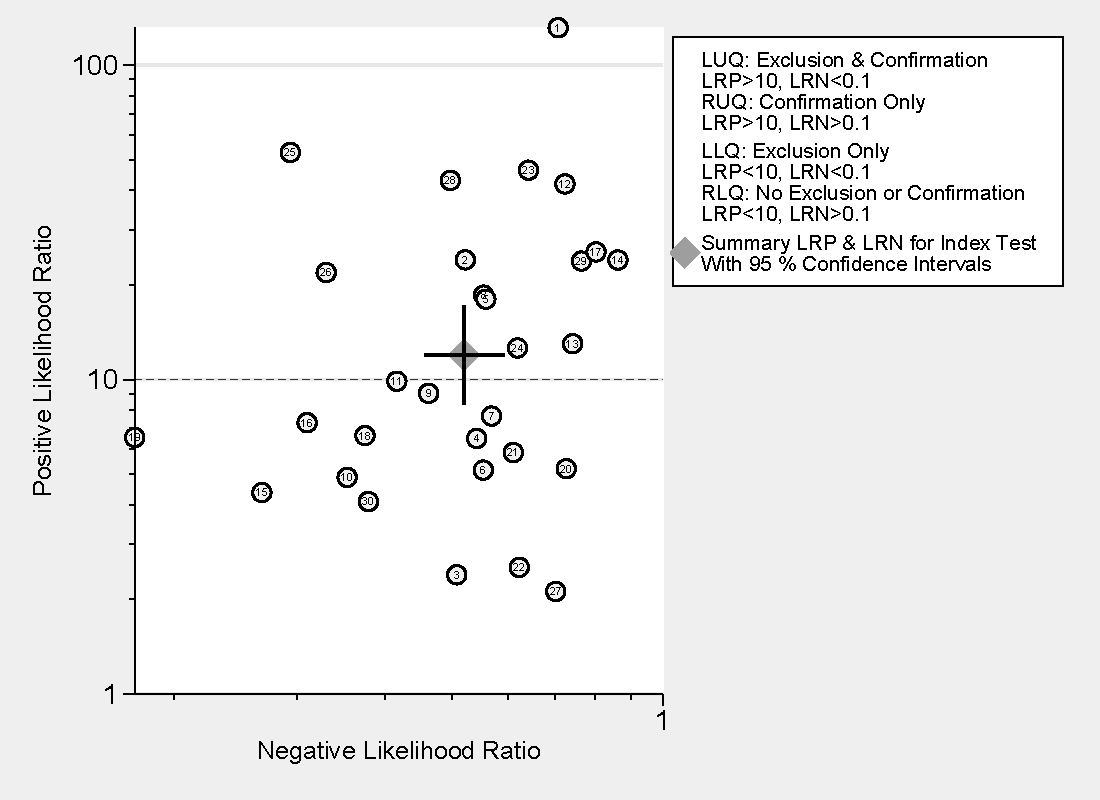
 **Supplementary Figure 8B.** Likelihood-ratio scatter plot for HbA1c in the pairwise meta-analysis, showing that HbA1c has both rule-in and rule-out value for T2DM. The position of the pooled estimates in the likelihood-ratio scatter plot indicates that HbA1c provides meaningful information for both confirming and excluding T2DM, suggesting favorable overall diagnostic performance as a stand-alone screening test.


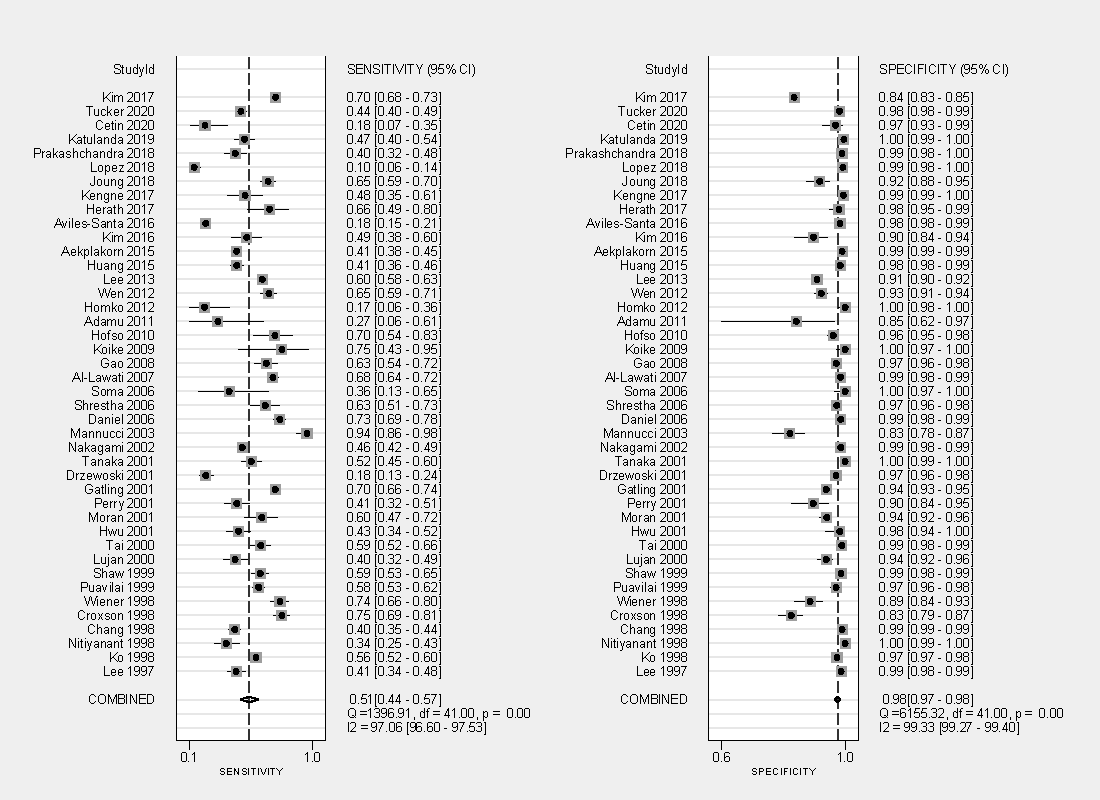
 **Supplementary Figure 2C.** Forest plots of pooled sensitivity and specificity for FPG in the pairwise meta-analysis.


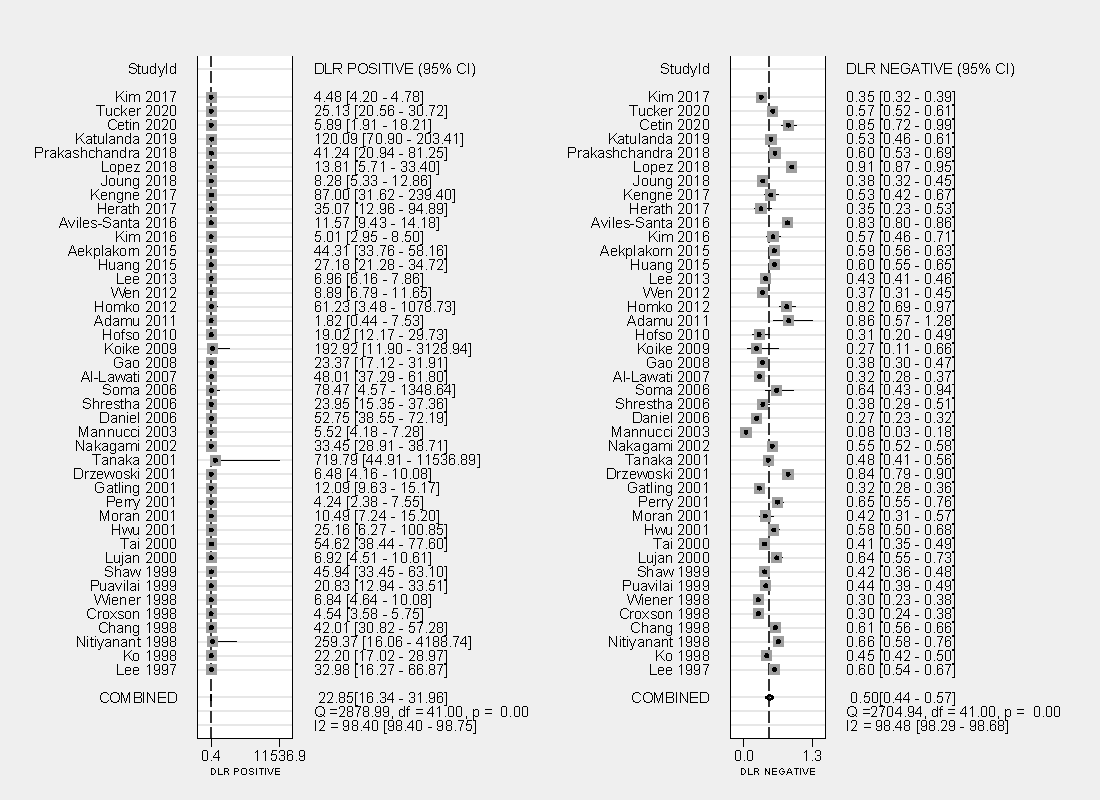


**Supplementary Figure 3C.** Forest plots of pooled positive likelihood ratio (LR+) and negative likelihood ratio (LR−) for FPG in the pairwise meta-analysis.


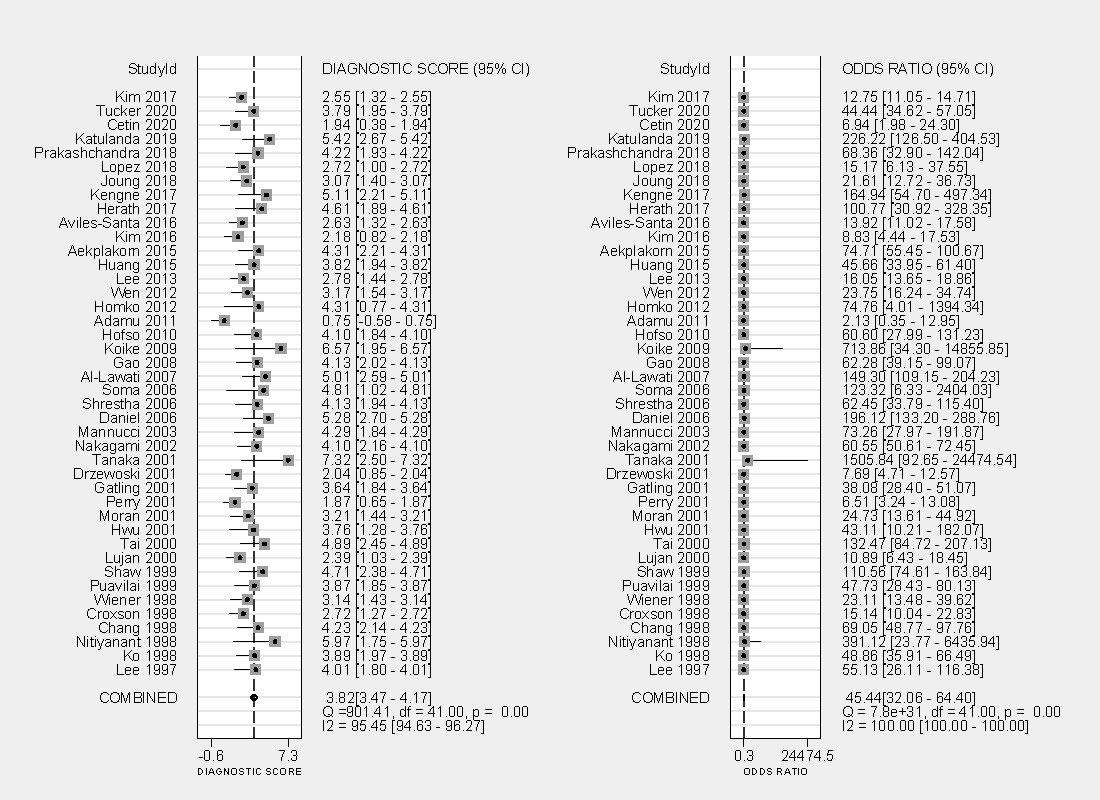


**Supplementary Figure 4C.** Forest plot of pooled diagnostic odds ratio (DOR) for FPG in the pairwise meta-analysis.


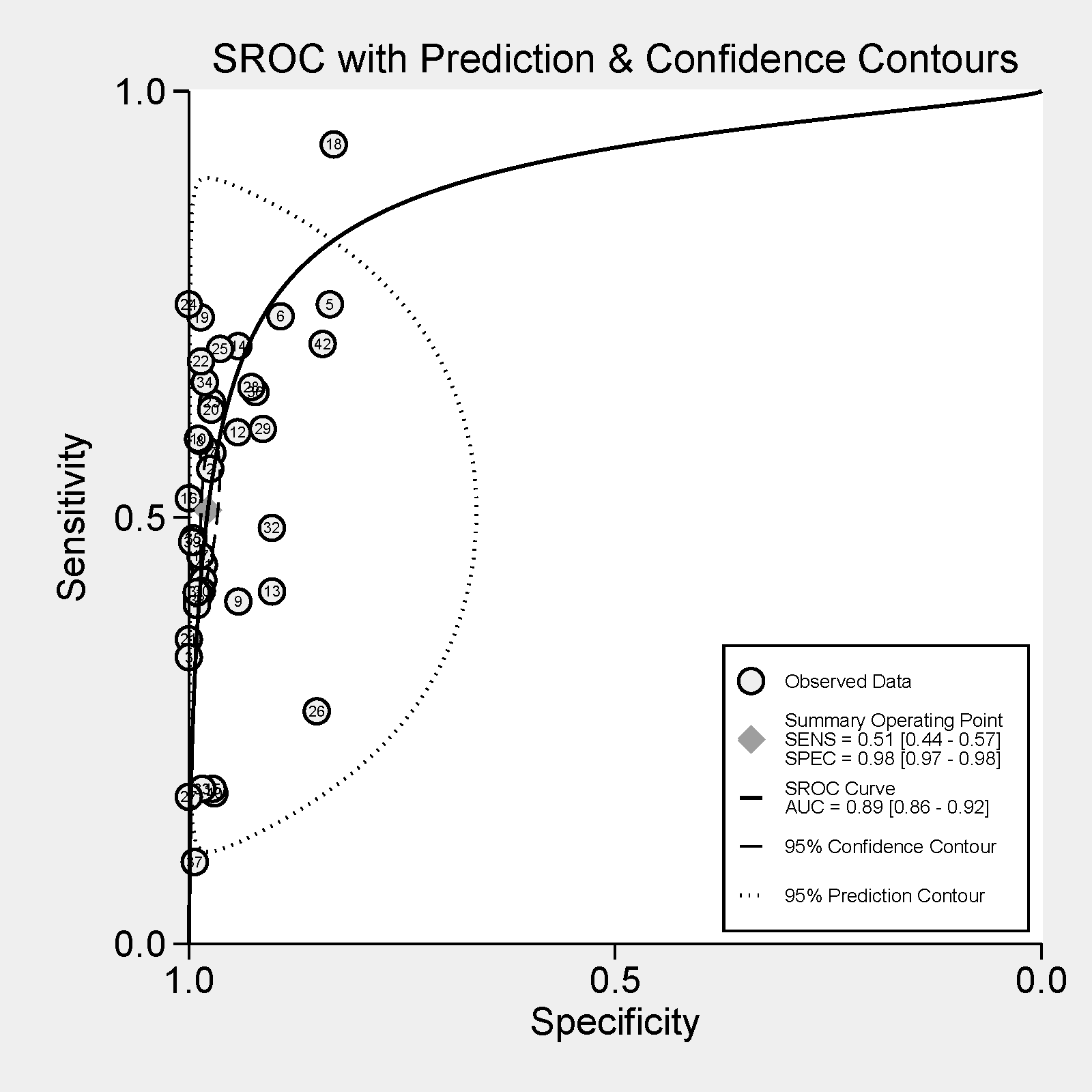


**Supplementary Figure 5C.** Summary receiver operating characteristic (SROC) curve for FPG in the pairwise meta-analysis.


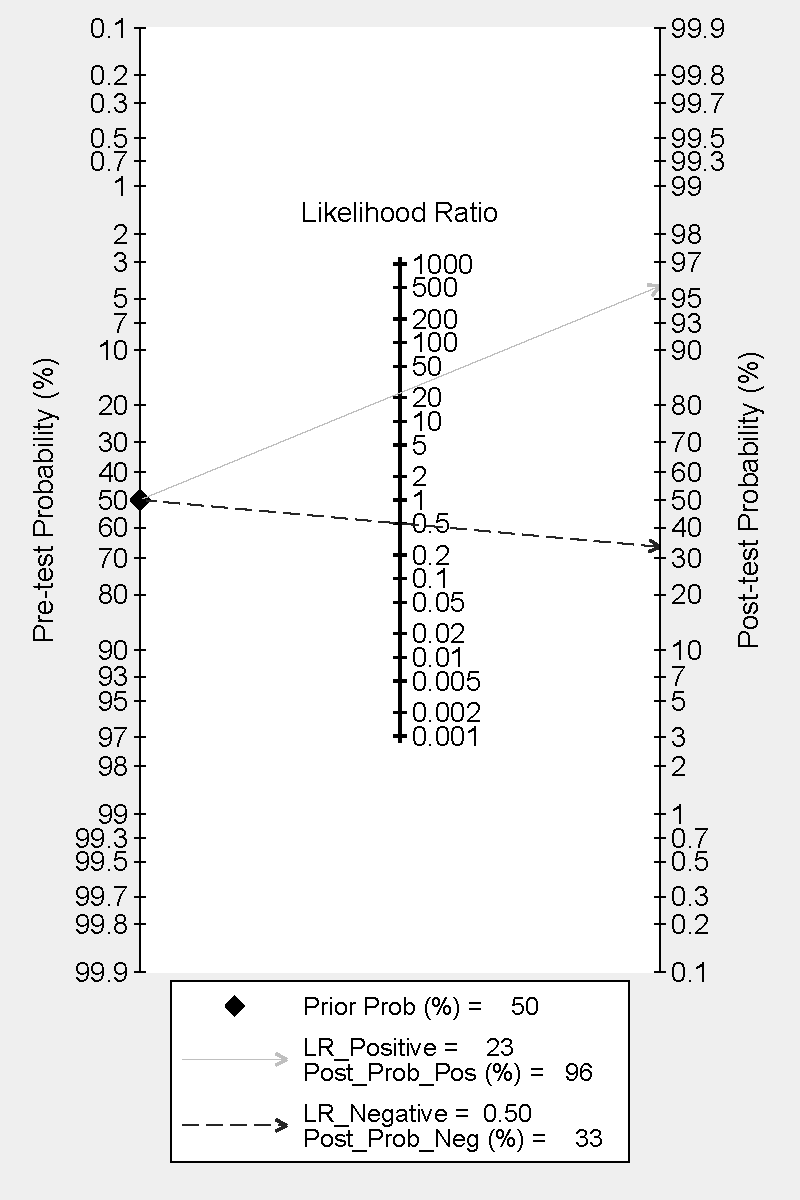


**Supplementary Figure 6C.** Fagan nomogram for FPG in the pairwise meta-analysis, showing its potential clinical utility for diagnosing T2DM. Based on the pooled positive and negative likelihood ratios, a positive FPG result markedly increased the post-test probability of T2DM, whereas a negative result substantially reduced the post-test probability, indicating that FPG may be clinically useful for both confirming and excluding T2DM in appropriate screening settings.


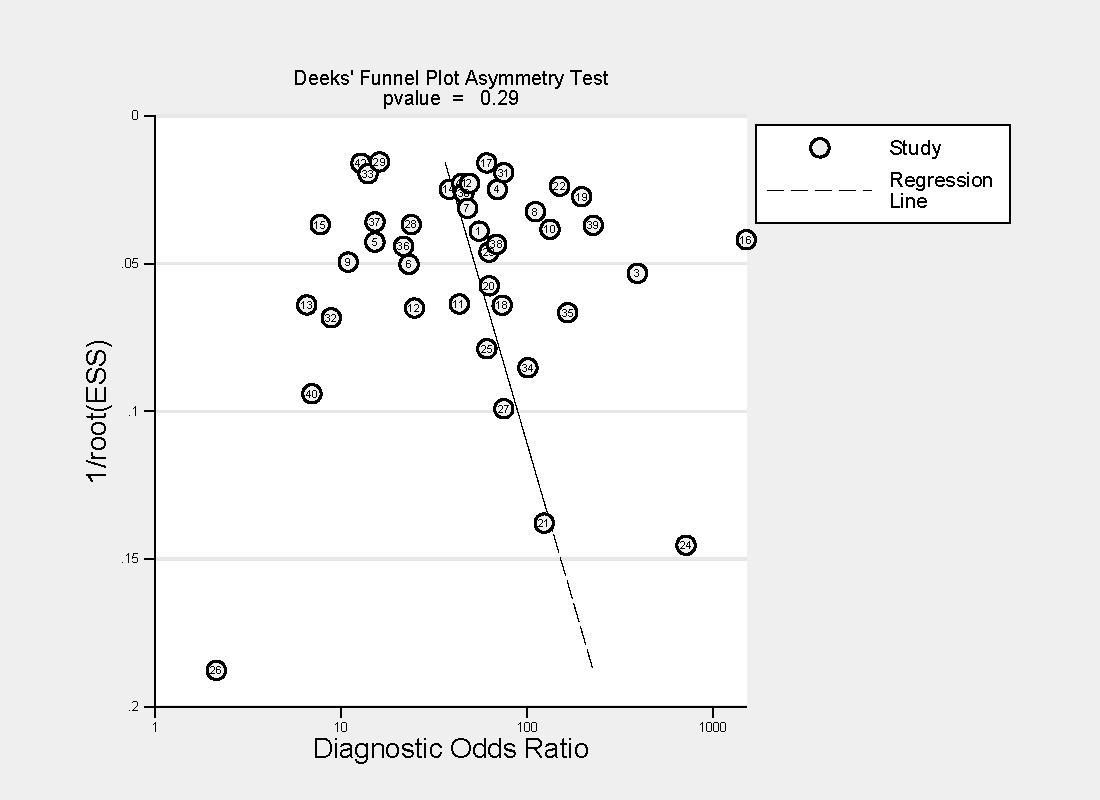
 **Supplementary Figure 7C.** Deeks’ funnel plot for publication bias assessment of FPG in the pairwise meta-analysis.


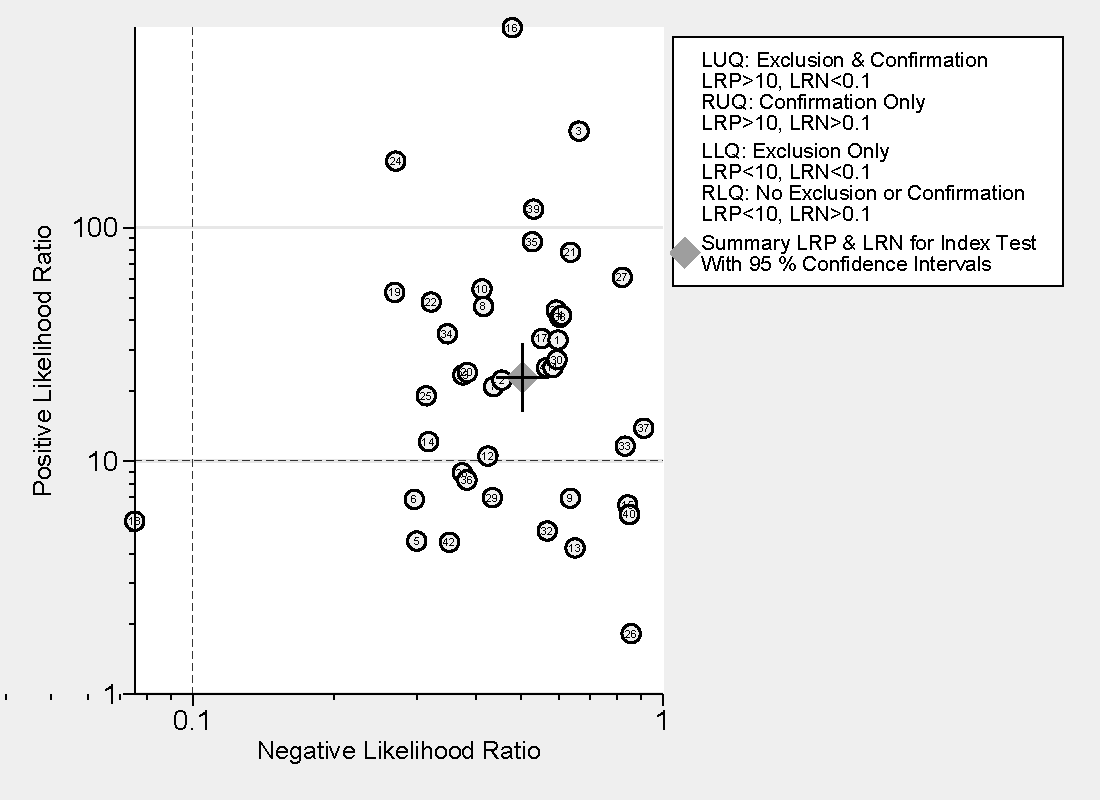
 **Supplementary Figure 8C.** Likelihood-ratio scatter plot for FPG in the pairwise meta-analysis, showing that FPG has both rule-in and rule-out value for T2DM. The position of the pooled estimates in the likelihood-ratio scatter plot indicates that FPG provides meaningful information for both confirming and excluding T2DM, suggesting favorable overall diagnostic performance as a stand-alone screening test.


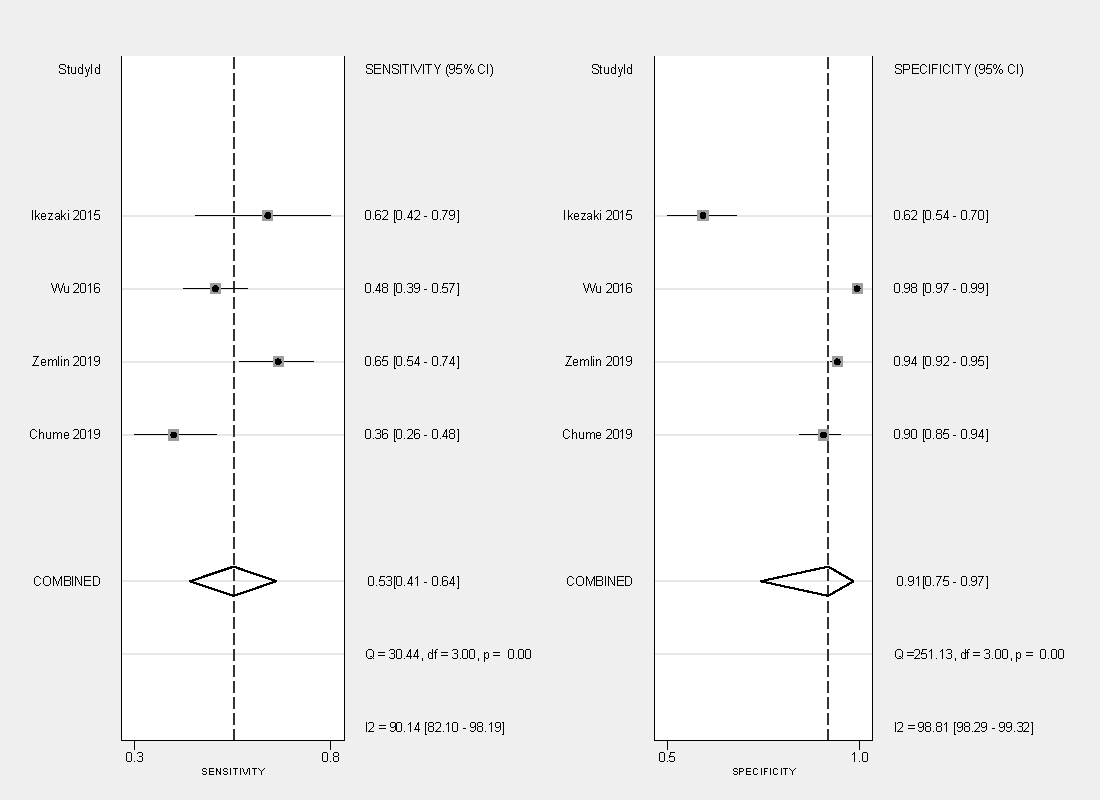
 **Supplementary Figure 2D.** Forest plots of pooled sensitivity and specificity for GA in the pairwise meta-analysis.


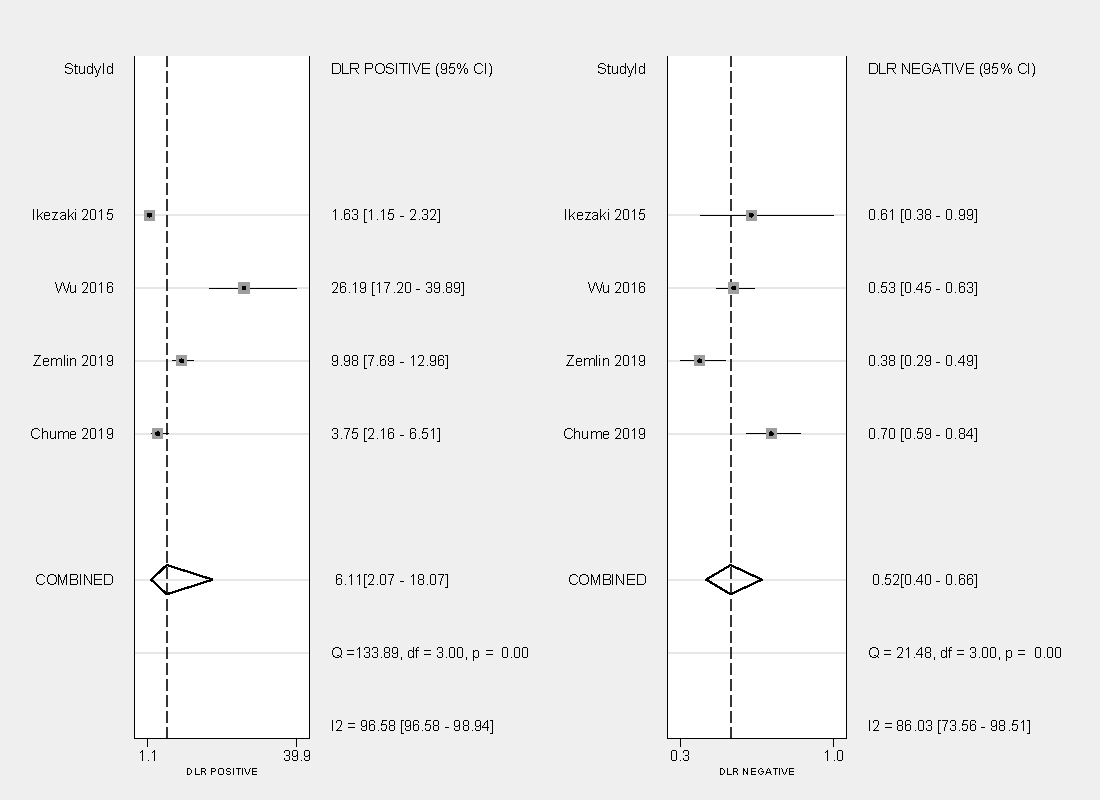


**Supplementary Figure 3D.** Forest plots of pooled positive likelihood ratio (LR+) and negative likelihood ratio (LR−) for GA in the pairwise meta-analysis.


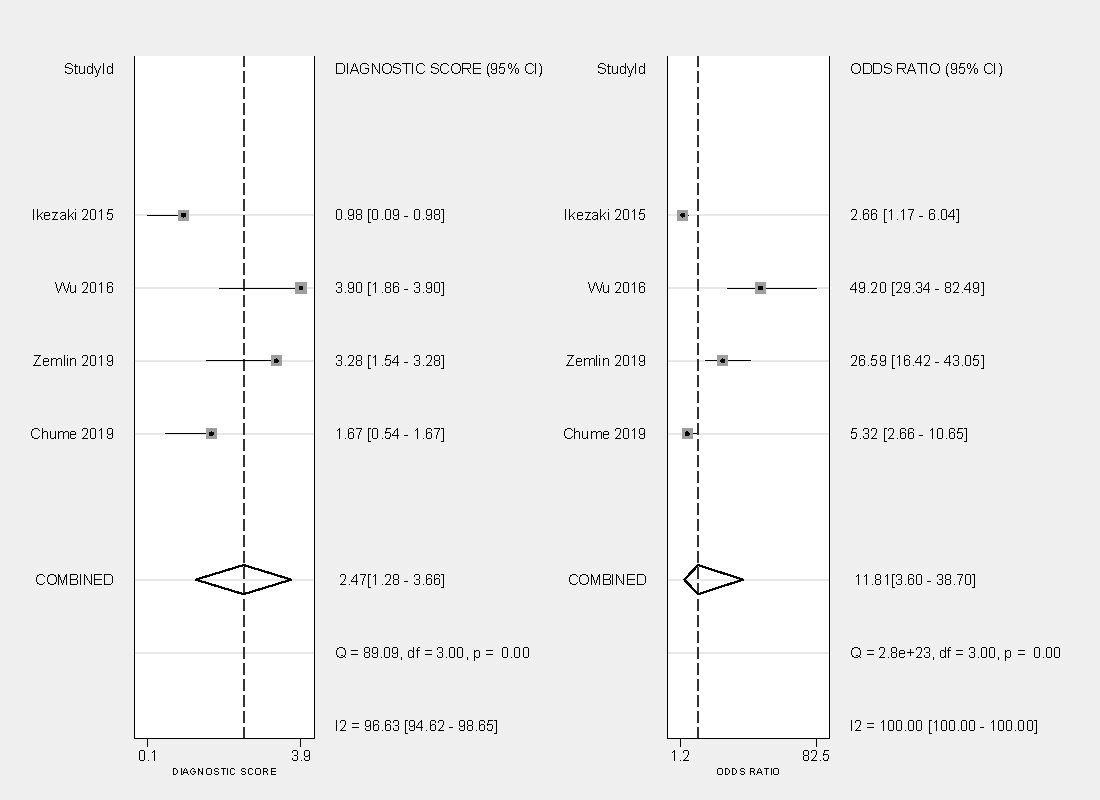


**Supplementary Figure 4D.** Forest plot of pooled diagnostic odds ratio (DOR) for GA in the pairwise meta-analysis.


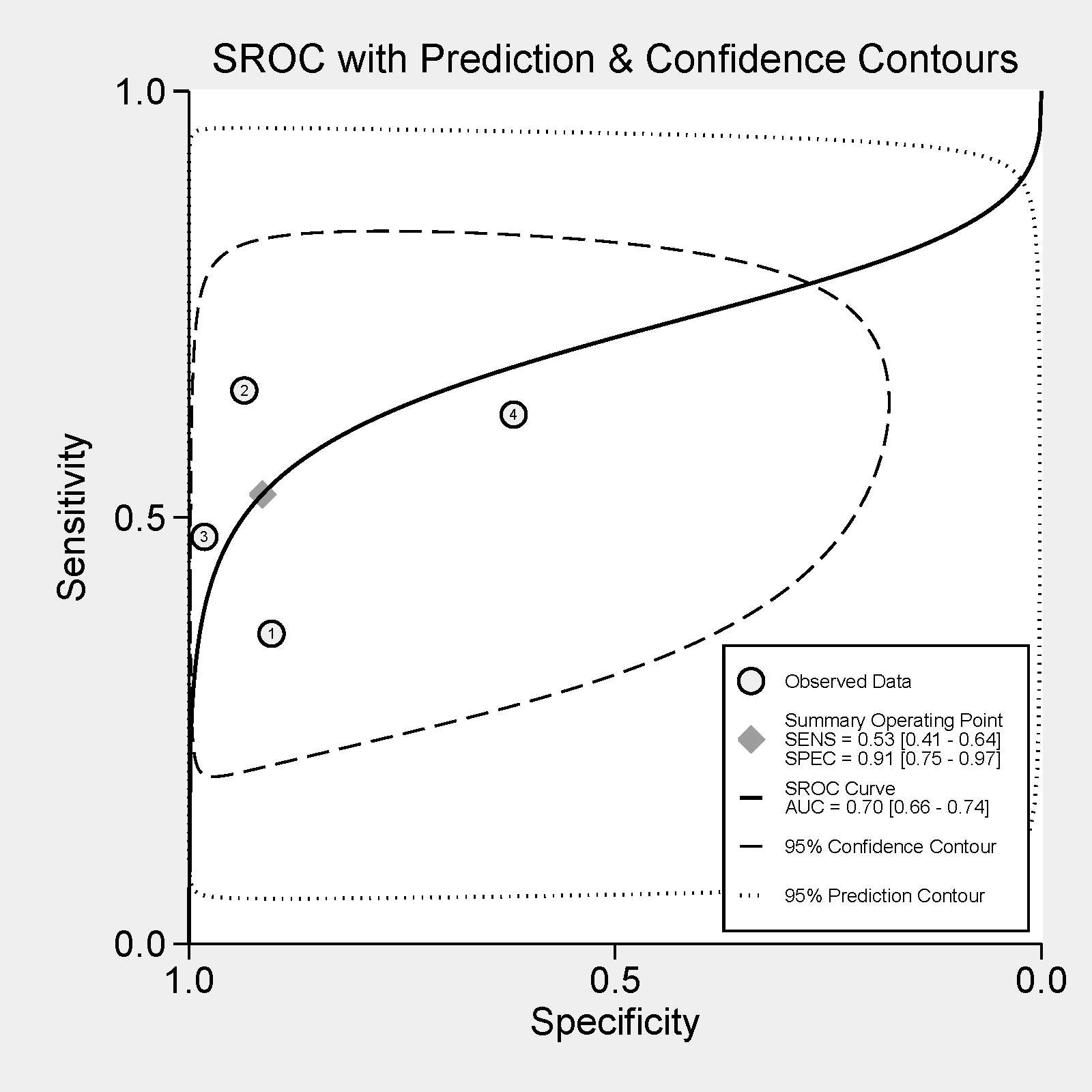


**Supplementary Figure 5D.** Summary receiver operating characteristic (SROC) curve for GA in the pairwise meta-analysis.


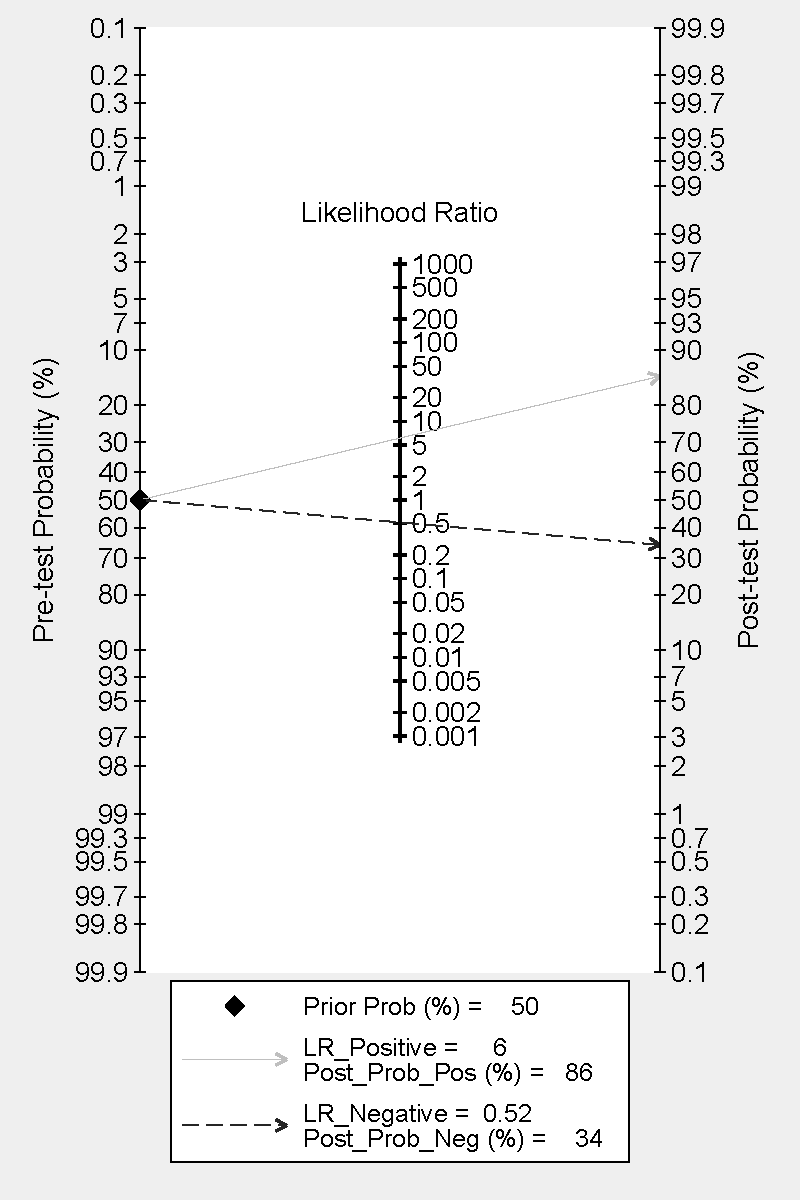


**Supplementary Figure 6D.** Fagan nomogram for GA in the pairwise meta-analysis, showing its potential clinical utility for diagnosing T2DM. Based on the pooled positive and negative likelihood ratios, a positive GA result markedly increased the post-test probability of T2DM, whereas a negative result substantially reduced the post-test probability, indicating that GA may be clinically useful for both confirming and excluding T2DM in appropriate screening settings.


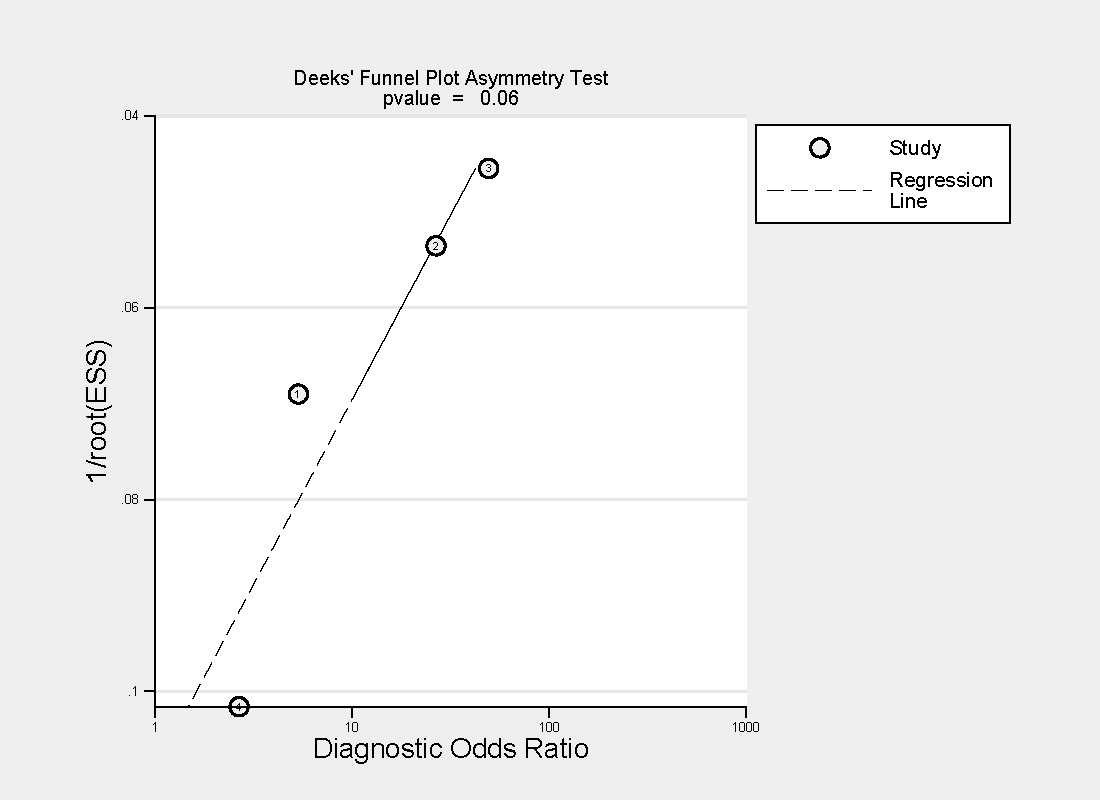
 **Supplementary Figure 7D.** Deeks’ funnel plot for publication bias assessment of GA in the pairwise meta-analysis.


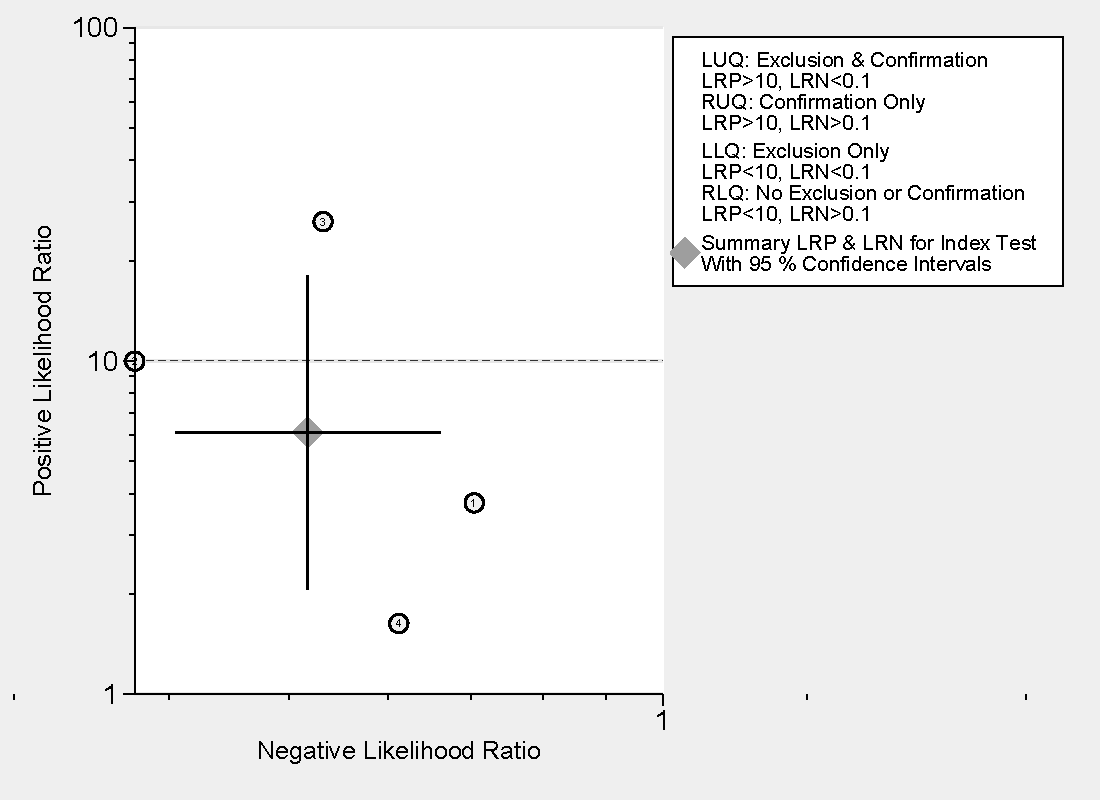
 **Supplementary Figure 8D.** Likelihood-ratio scatter plot for GA in the pairwise meta-analysis, showing that GA has both rule-in and rule-out value for T2DM. The position of the pooled estimates in the likelihood-ratio scatter plot indicates that GA provides meaningful information for both confirming and excluding T2DM, suggesting favorable overall diagnostic performance as a stand-alone screening test.


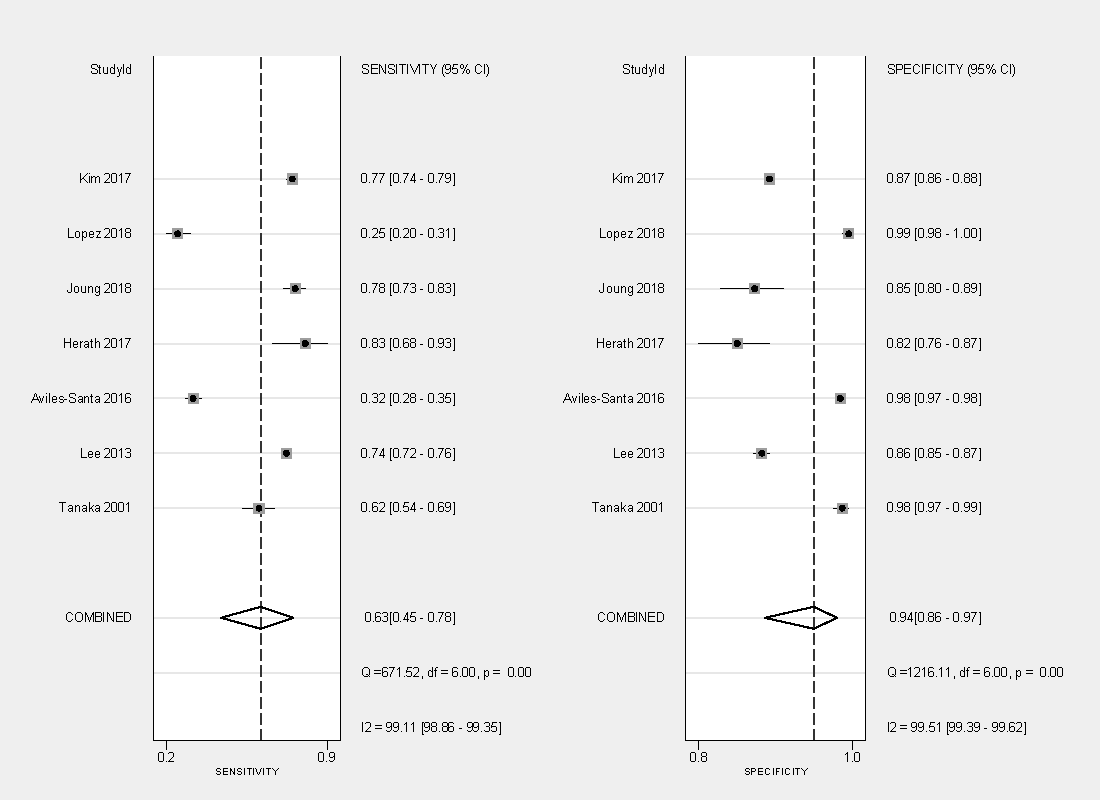
 **Supplementary Figure 2E.** Forest plots of pooled sensitivity and specificity for HbA1c or FPG in the pairwise meta-analysis.


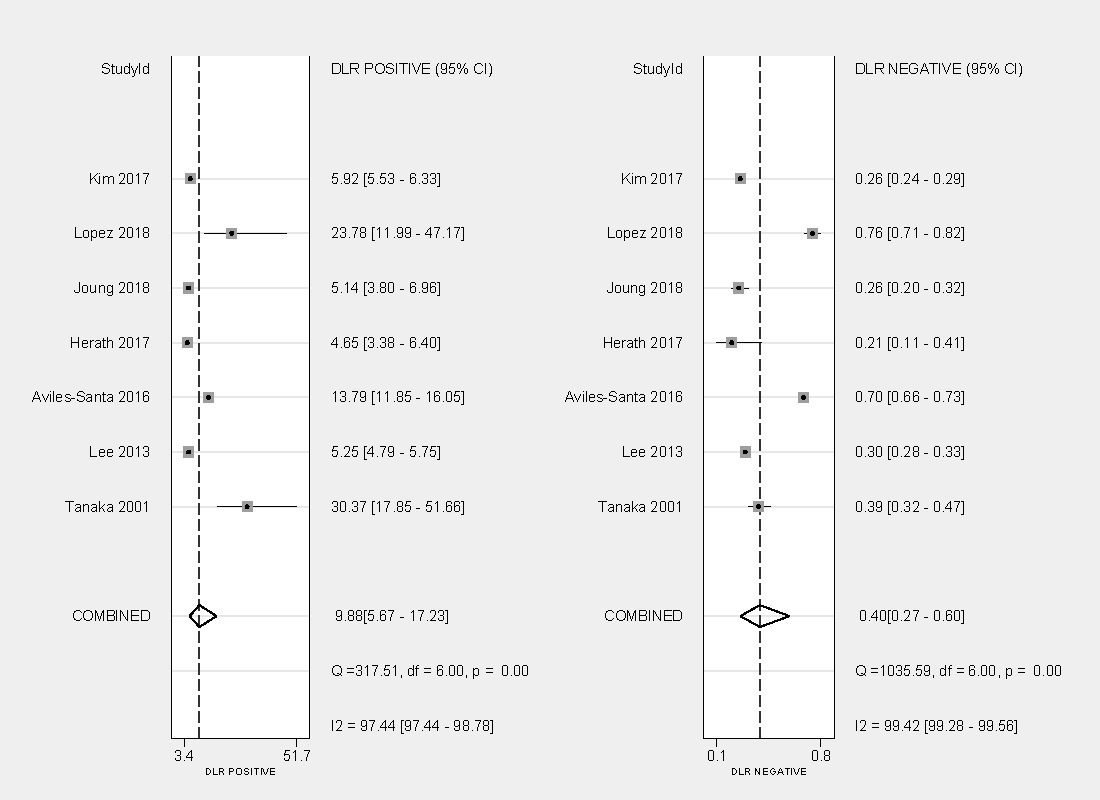


**Supplementary Figure 3E.** Forest plots of pooled positive likelihood ratio (LR+) and negative likelihood ratio (LR−) for HbA1c or FPG in the pairwise meta-analysis.


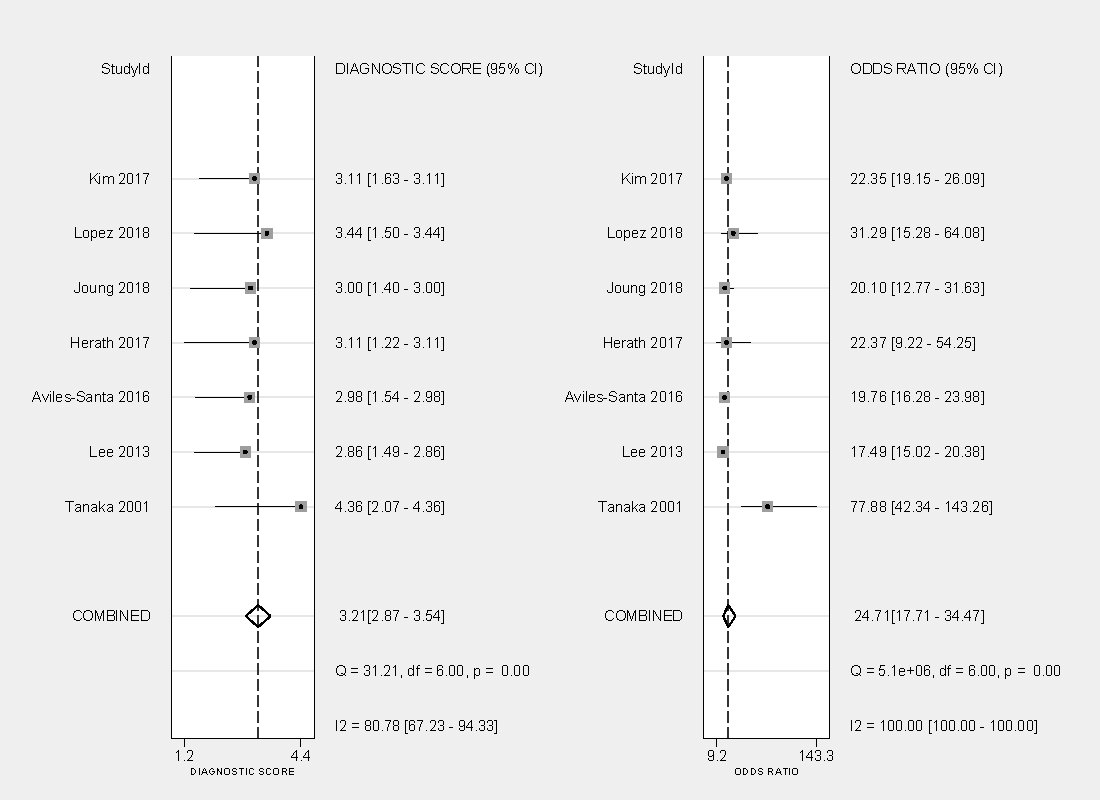


**Supplementary Figure 4E.** Forest plot of pooled diagnostic odds ratio (DOR) for HbA1c or FPG in the pairwise meta-analysis.


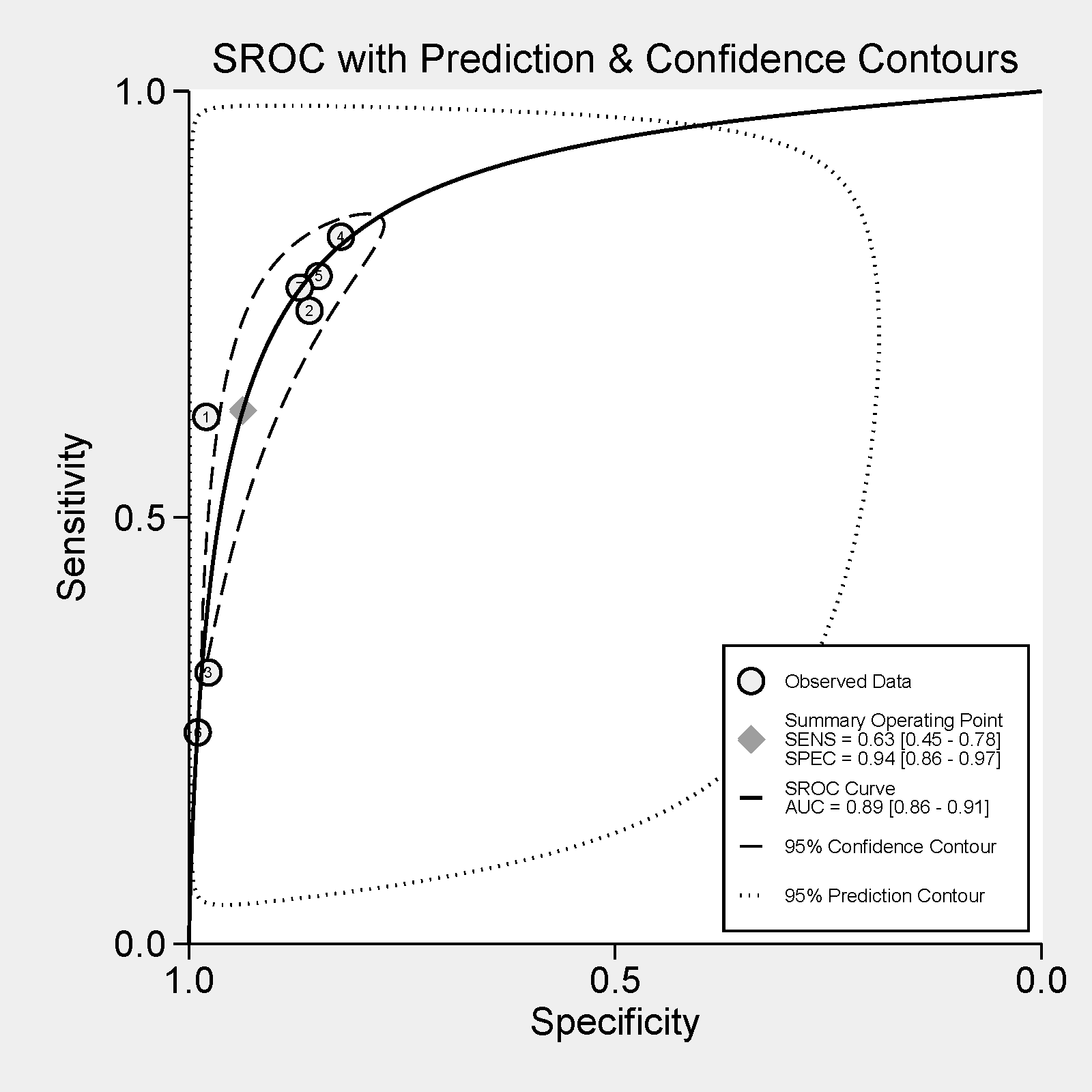


**Supplementary Figure 5E.** Summary receiver operating characteristic (SROC) curve for HbA1c or FPG in the pairwise meta-analysis.


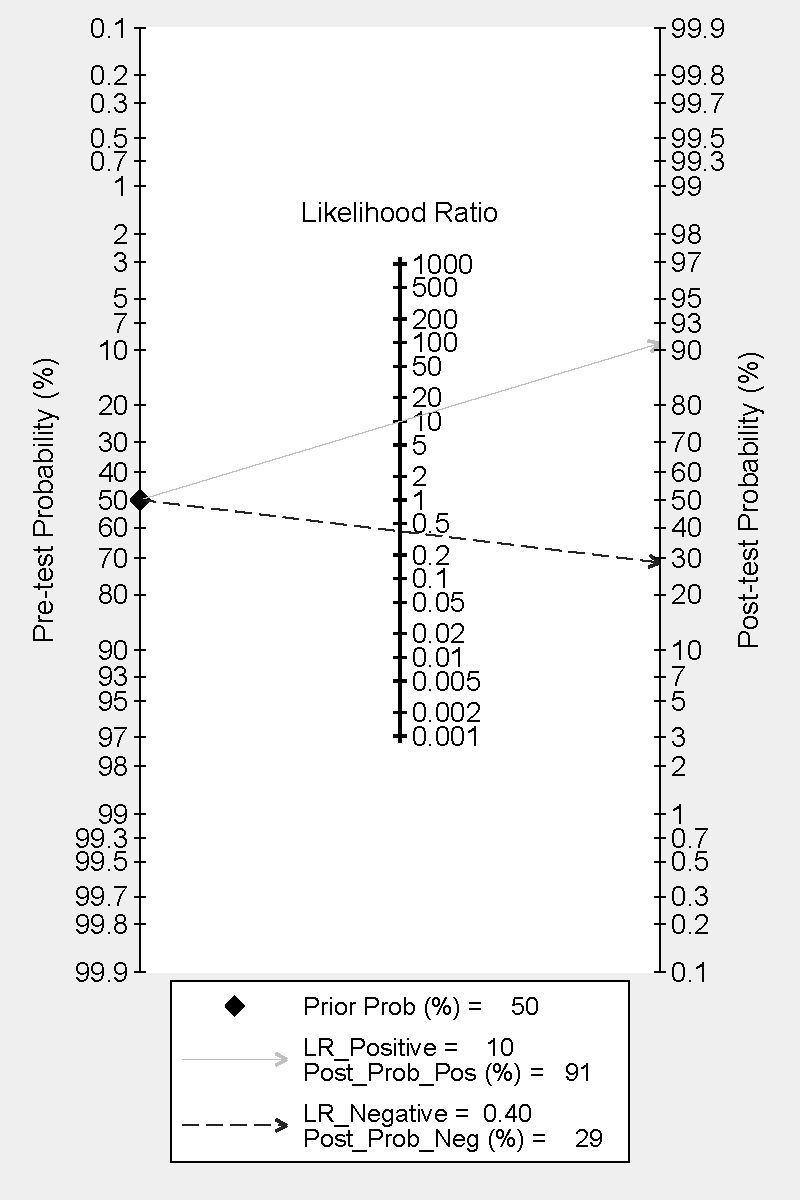


**Supplementary Figure 6E.** Fagan nomogram for HbA1c or FPG in the pairwise meta-analysis, showing its potential clinical utility for diagnosing T2DM. Based on the pooled positive and negative likelihood ratios, a positive HbA1c or FPG result markedly increased the post-test probability of T2DM, whereas a negative result substantially reduced the post-test probability, indicating that HbA1c or FPG may be clinically useful for both confirming and excluding T2DM in appropriate screening settings.


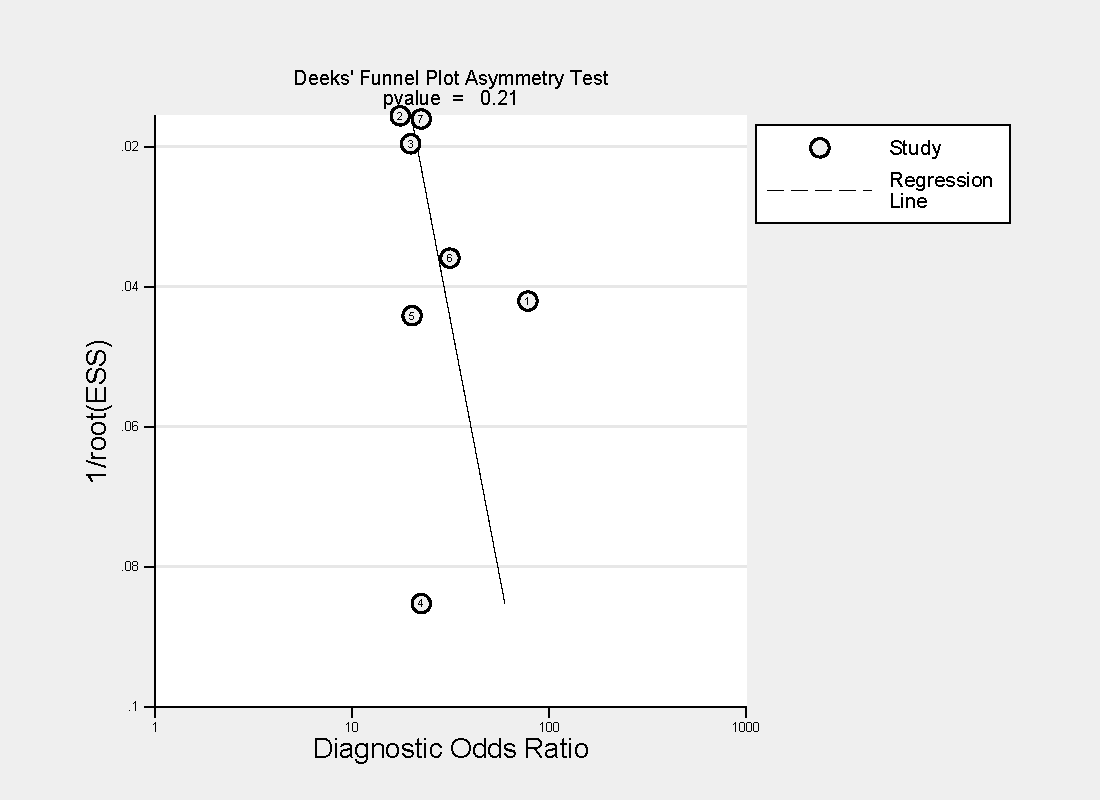
 **Supplementary Figure 7E.** Deeks’ funnel plot for publication bias assessment of HbA1c or FPG in the pairwise meta-analysis.


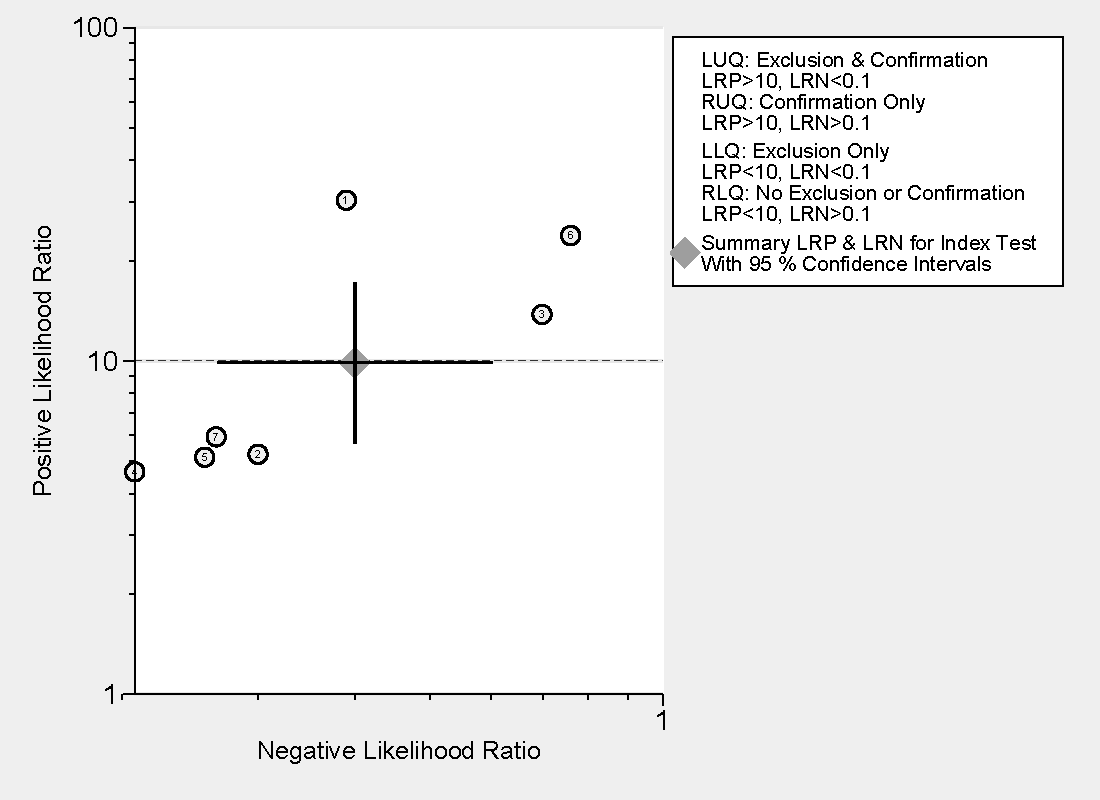
 **Supplementary Figure 8E.** Likelihood-ratio scatter plot for HbA1c or FPG in the pairwise meta-analysis, showing that HbA1c or FPG has both rule-in and rule-out value for T2DM. The position of the pooled estimates in the likelihood-ratio scatter plot indicates that HbA1c or FPG provides meaningful information for both confirming and excluding T2DM, suggesting favorable overall diagnostic performance as a stand-alone screening test.


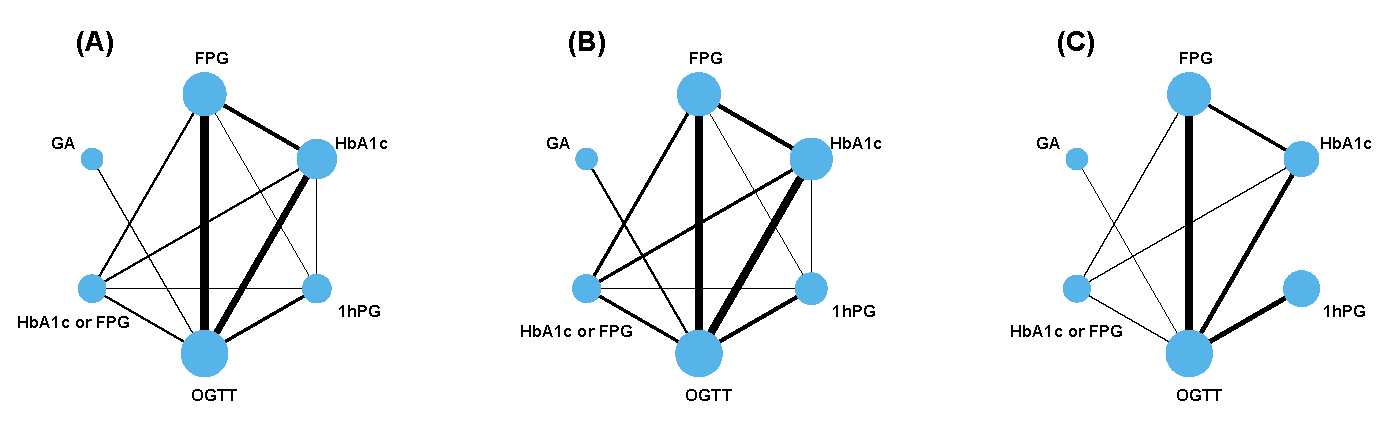


**Supplementary Figure 9.** Network plots for the subgroup analyses in the diagnostic accuracy network meta-analysis: (A) studies with a 1-h PG threshold ≥11.6 mmol/L; (B) studies in general populations; and (C) studies in high-risk populations.

1-h PG, 1-hour plasma glucose; HbA1c, glycated hemoglobin; FPG, fasting plasma glucose; GA, glycated albumin.


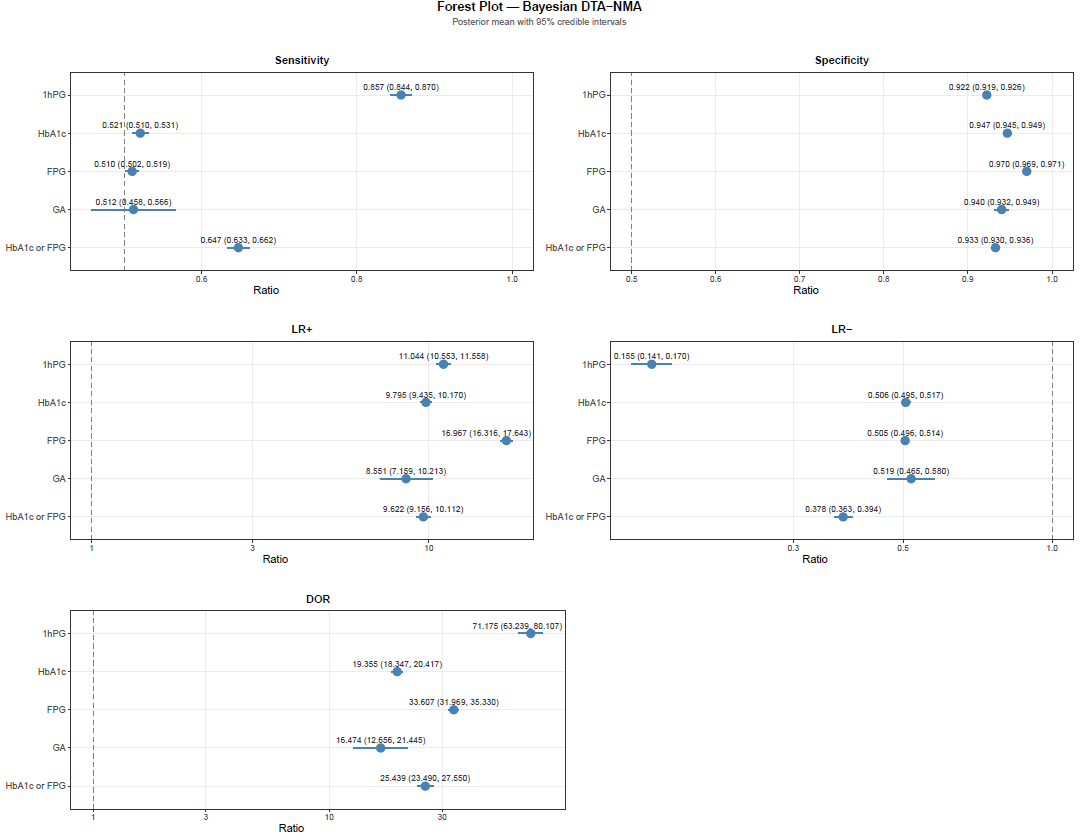


**Supplementary Figures 10.** Forest plots in subgroup analysis about studies with a 1-h PG threshold ≥11.6 mmol/L from the Bayesian diagnostic test accuracy network meta-analysis showing the pooled posterior mean estimates with 95% credible intervals for sensitivity (A), specificity (B), positive likelihood ratio (LR+) (C), negative likelihood ratio (LR−) (D), and diagnostic odds ratio (DOR) (E) across the five diagnostic strategies.

1-h PG, 1-hour plasma glucose; HbA1c, glycated hemoglobin; FPG, fasting plasma glucose; GA, glycated albumin.


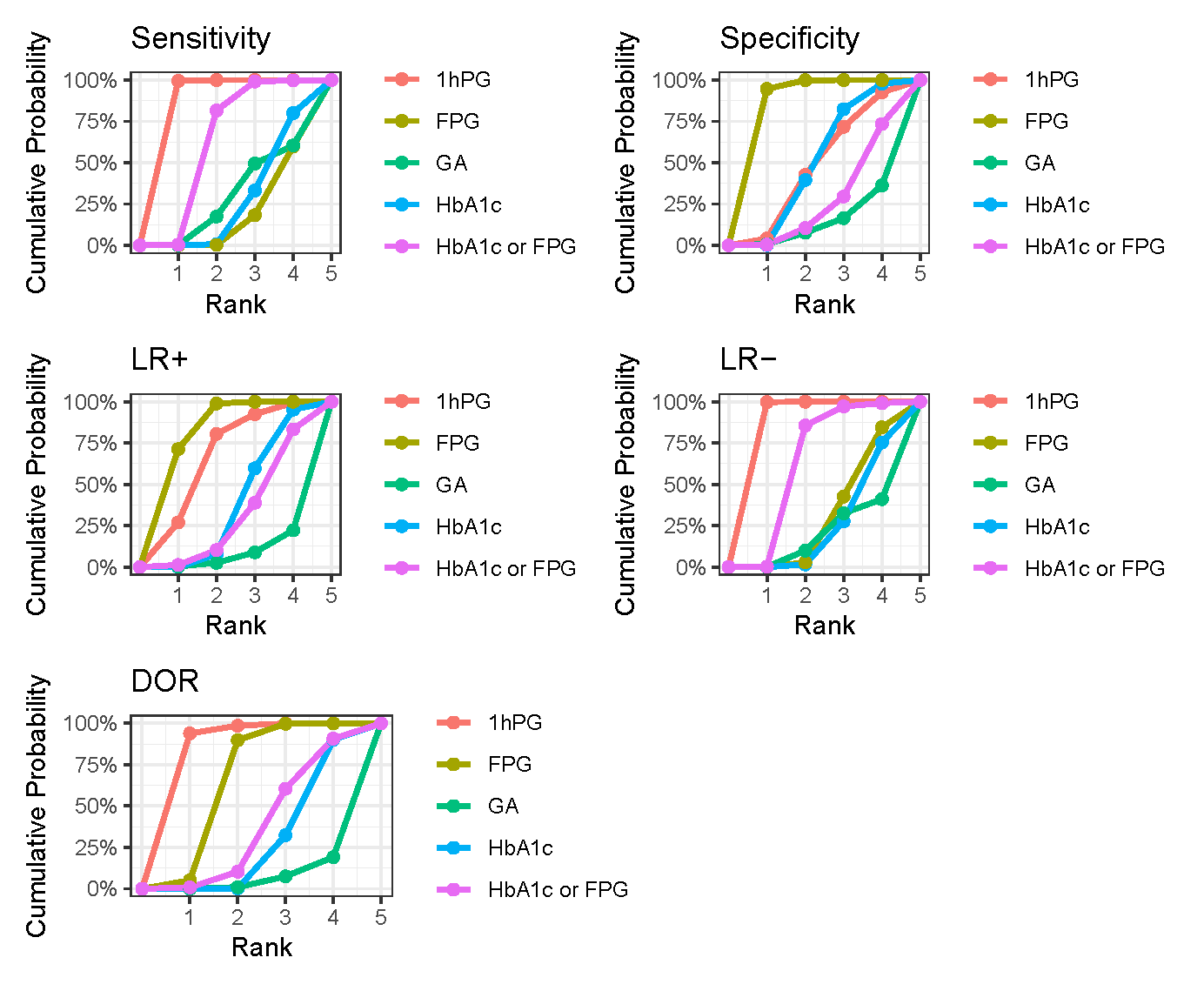
**Supplementary Figures 11.** Cumulative ranking curves for the five diagnostic strategies in subgroup analysis about studies with a 1-h PG threshold ≥11.6 mmol/L. Ranking probabilities are shown for sensitivity, specificity, positive likelihood ratio (LR+), negative likelihood ratio (LR−), and diagnostic odds ratio (DOR).

1-h PG, 1-hour plasma glucose; HbA1c, glycated hemoglobin; FPG, fasting plasma glucose; GA, glycated albumin.

**Supplementary Tables S3A–E.** League tables of relative diagnostic performance in subgroup analysis about studies with a 1-h PG threshold ≥11.6 mmol/L. Relative comparisons among 1-h PG, HbA1c, FPG, GA, and combined HbA1c or FPG are presented for sensitivity (A), specificity (B), positive likelihood ratio (LR+) (C), negative likelihood ratio (LR−) (D), and diagnostic odds ratio (DOR) (E), with posterior estimates and 95% credible intervals.

1-h PG, 1-hour plasma glucose; HbA1c, glycated hemoglobin; FPG, fasting plasma glucose; GA, glycated albumin.


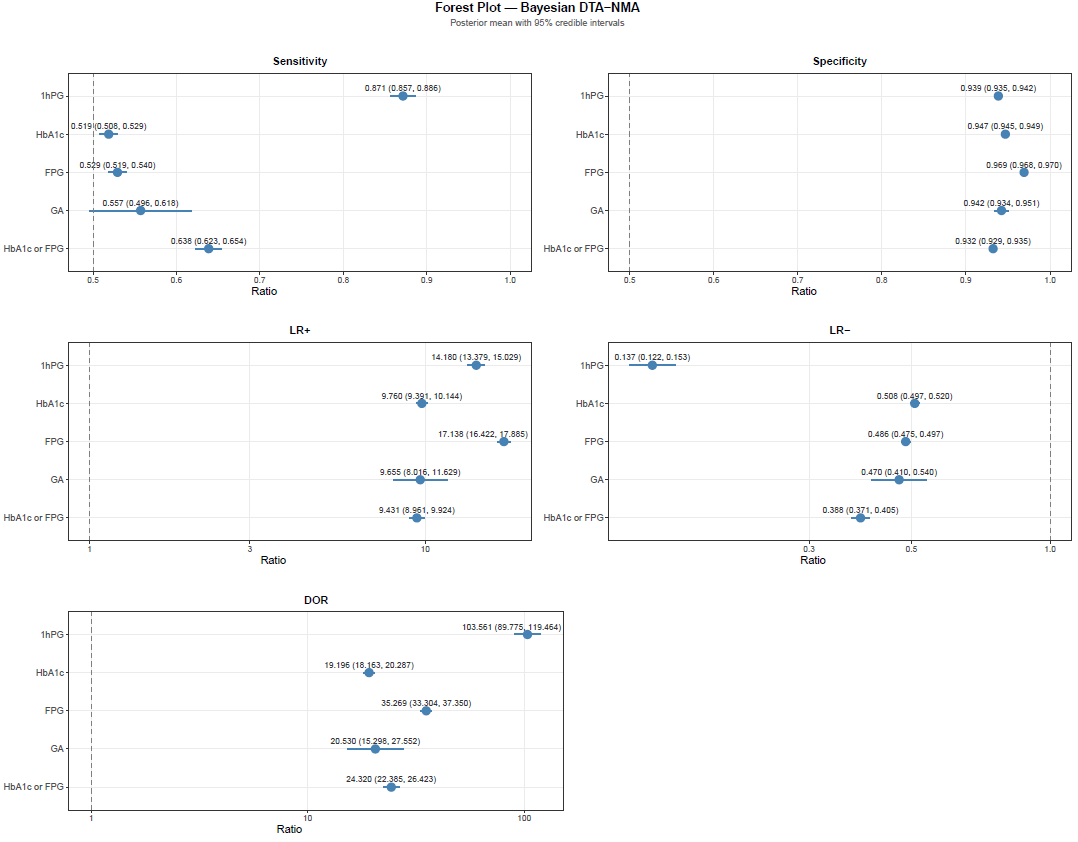
**Supplementary Figures 12.** Forest plots in subgroup analysis about studies in general populations from the Bayesian diagnostic test accuracy network meta-analysis showing the pooled posterior mean estimates with 95% credible intervals for sensitivity (A), specificity (B), positive likelihood ratio (LR+) (C), negative likelihood ratio (LR−) (D), and diagnostic odds ratio (DOR) (E) across the five diagnostic strategies.

1-h PG, 1-hour plasma glucose; HbA1c, glycated hemoglobin; FPG, fasting plasma glucose; GA, glycated albumin.


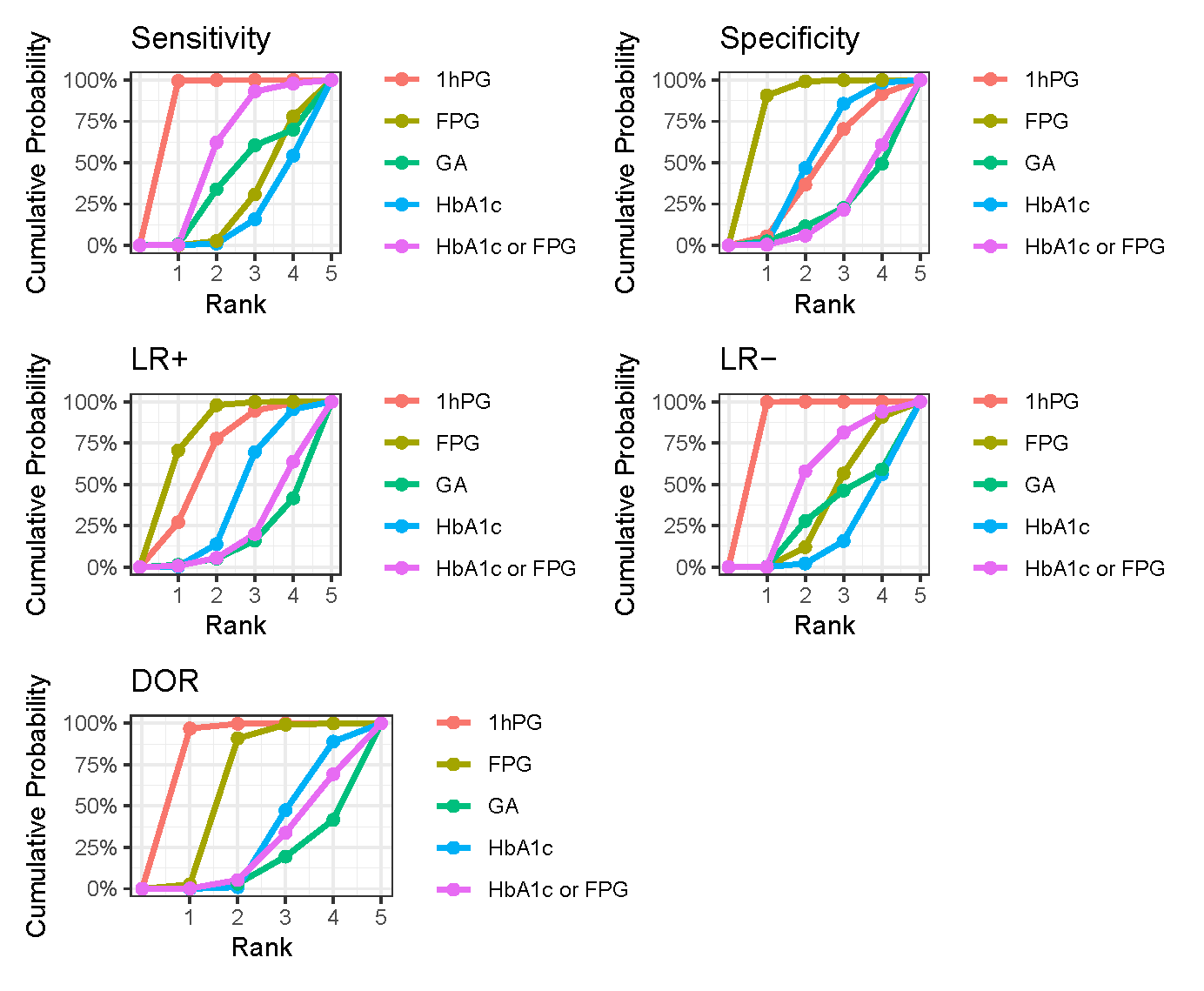
**Supplementary Figures 13.** Cumulative ranking curves for the five diagnostic strategies in subgroup analysis about studies in general populations. Ranking probabilities are shown for sensitivity, specificity, positive likelihood ratio (LR+), negative likelihood ratio (LR−), and diagnostic odds ratio (DOR).

1-h PG, 1-hour plasma glucose; HbA1c, glycated hemoglobin; FPG, fasting plasma glucose; GA, glycated albumin.

**Supplementary Tables S4A–E.** League tables of relative diagnostic performance in subgroup analysis about studies in general populations. Relative comparisons among 1-h PG, HbA1c, FPG, GA, and combined HbA1c or FPG are presented for sensitivity (A), specificity (B), positive likelihood ratio (LR+) (C), negative likelihood ratio (LR−) (D), and diagnostic odds ratio (DOR) (E), with posterior estimates and 95% credible intervals.

1-h PG, 1-hour plasma glucose; HbA1c, glycated hemoglobin; FPG, fasting plasma glucose; GA, glycated albumin.


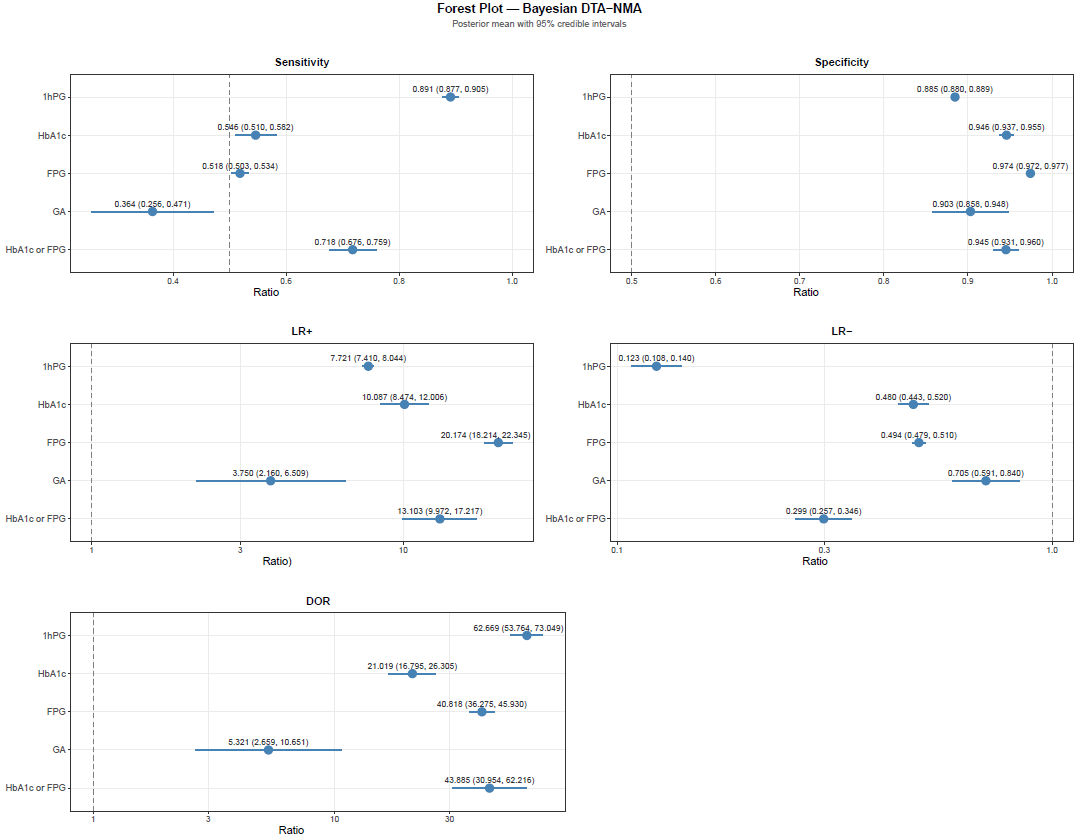
**Supplementary Figures 14.** Forest plots in subgroup analysis about studies in high-risk populations from the Bayesian diagnostic test accuracy network meta-analysis showing the pooled posterior mean estimates with 95% credible intervals for sensitivity (A), specificity (B), positive likelihood ratio (LR+) (C), negative likelihood ratio (LR−) (D), and diagnostic odds ratio (DOR) (E) across the five diagnostic strategies.

1-h PG, 1-hour plasma glucose; HbA1c, glycated hemoglobin; FPG, fasting plasma glucose; GA, glycated albumin.


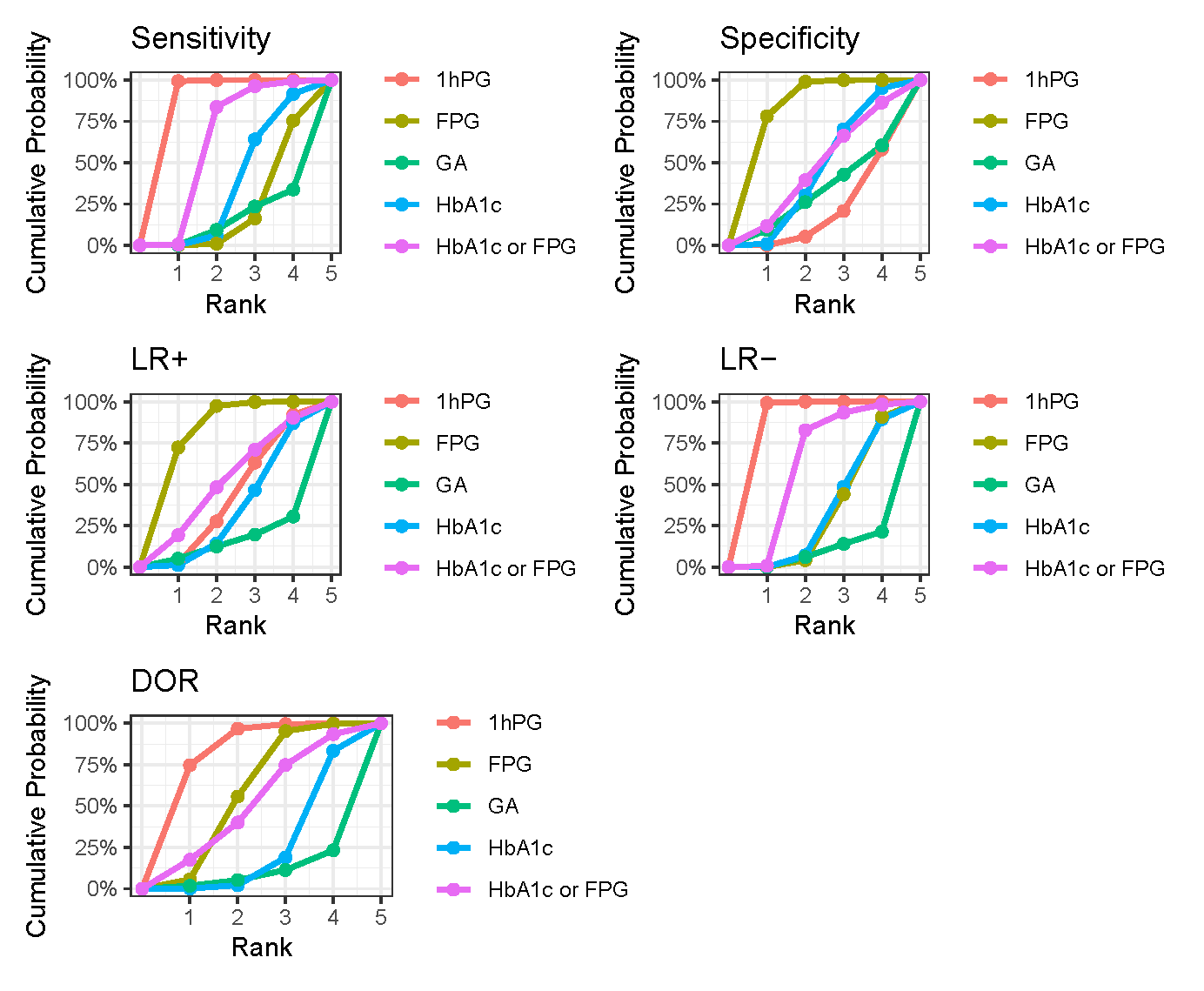
 **Supplementary Figures 15.** Cumulative ranking curves for the five diagnostic strategies in subgroup analysis about studies in high-risk populations. Ranking probabilities are shown for sensitivity, specificity, positive likelihood ratio (LR+), negative likelihood ratio (LR−), and diagnostic odds ratio (DOR).

1-h PG, 1-hour plasma glucose; HbA1c, glycated hemoglobin; FPG, fasting plasma glucose; GA, glycated albumin.

**Supplementary Tables S5A–E.** League tables of relative diagnostic performance in subgroup analysis about studies in high-risk populations. Relative comparisons among 1-h PG, HbA1c, FPG, GA, and combined HbA1c or FPG are presented for sensitivity (A), specificity (B), positive likelihood ratio (LR+) (C), negative likelihood ratio (LR−) (D), and diagnostic odds ratio (DOR) (E), with posterior estimates and 95% credible intervals.

1-h PG, 1-hour plasma glucose; HbA1c, glycated hemoglobin; FPG, fasting plasma glucose; GA, glycated albumin.


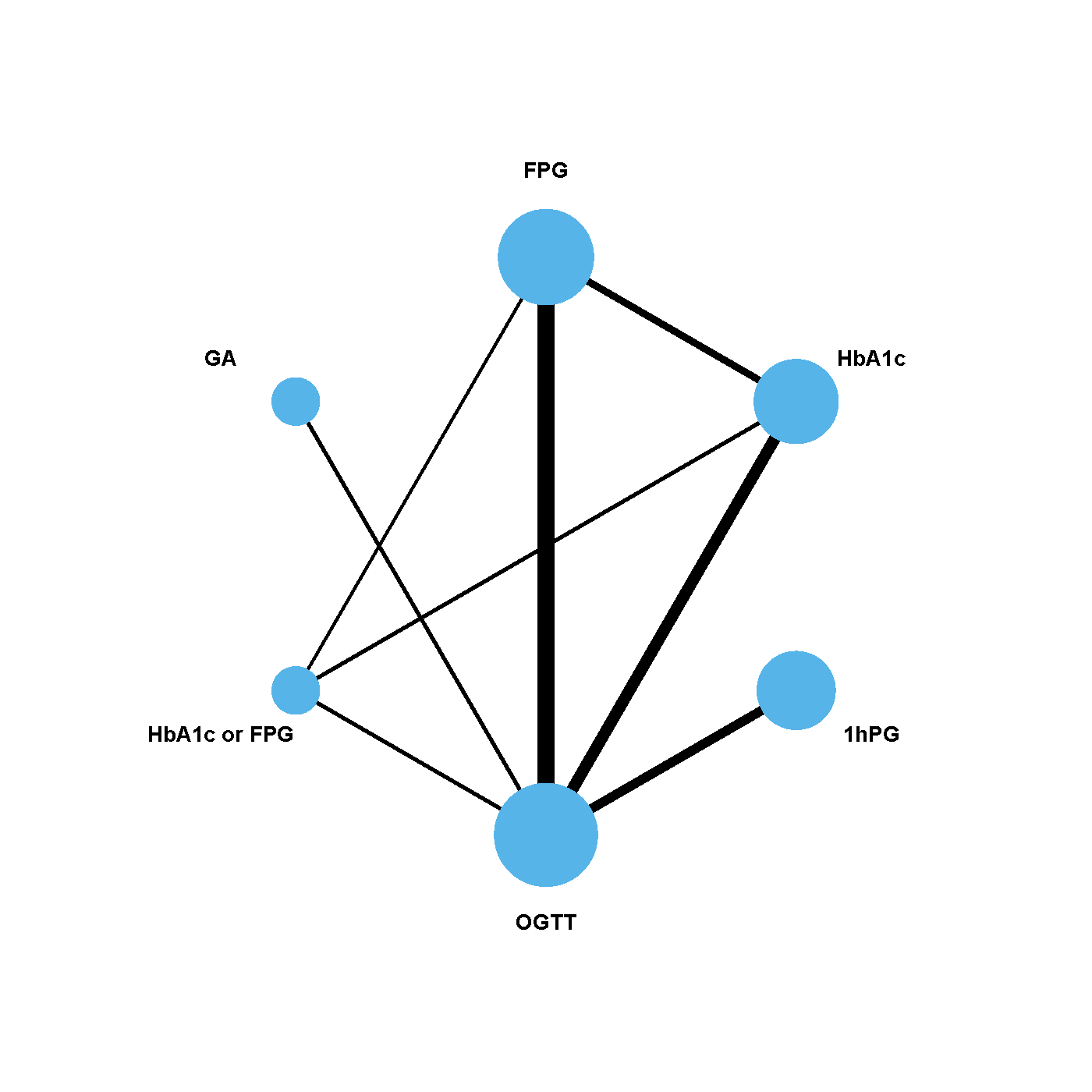
**Supplementary Figure 16.** Network plot of the diagnostic accuracy network meta-analysis in sensitivity analysis. Nodes represent the index tests and the reference standard, and connecting lines indicate direct evidence contributed by the included studies. The diagnostic strategies included 1-h PG, HbA1c, FPG, GA, and combined HbA1c or FPG, with OGTT as the reference standard.

1-h PG, 1-hour plasma glucose; HbA1c, glycated hemoglobin; FPG, fasting plasma glucose; GA, glycated albumin; OGTT, oral glucose tolerance test.


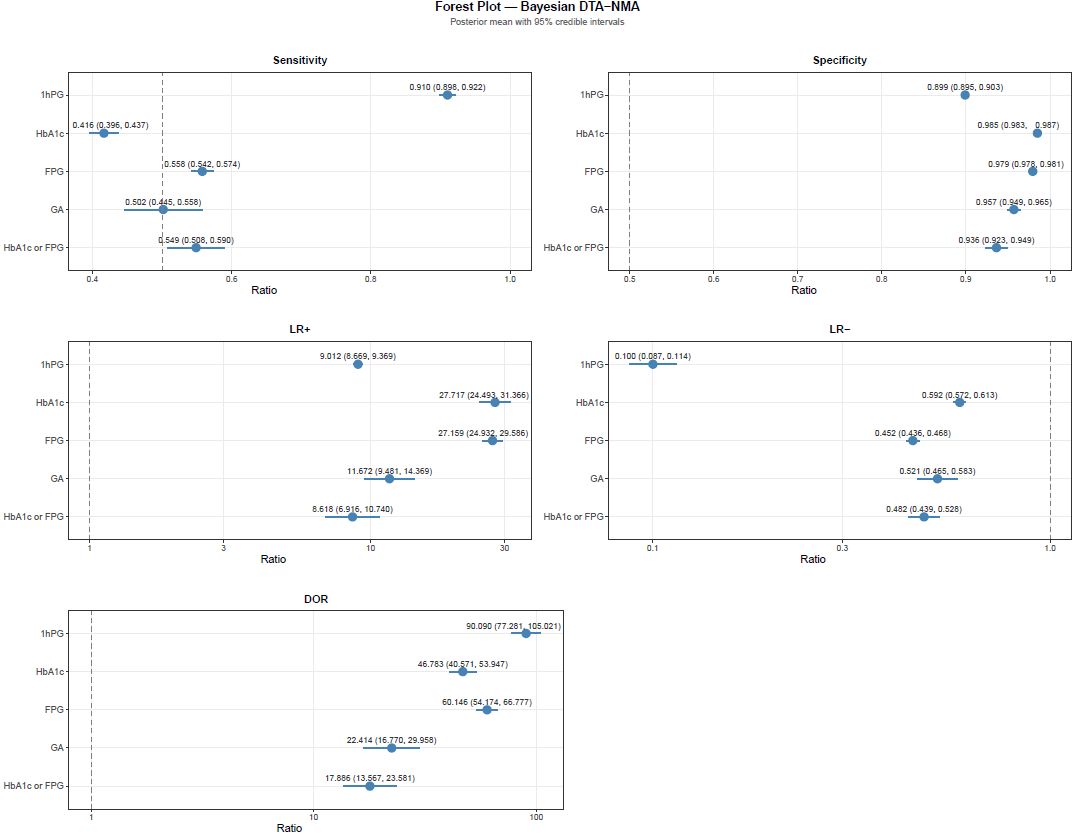
 **Supplementary Figure 17.** Forest plots in sensitivity analysis from the Bayesian diagnostic test accuracy network meta-analysis showing the pooled posterior mean estimates with 95% credible intervals for sensitivity (A), specificity (B), positive likelihood ratio (LR+) (C), negative likelihood ratio (LR−) (D), and diagnostic odds ratio (DOR) (E) across the five diagnostic strategies.

1-h PG, 1-hour plasma glucose; HbA1c, glycated hemoglobin; FPG, fasting plasma glucose; GA, glycated albumin.


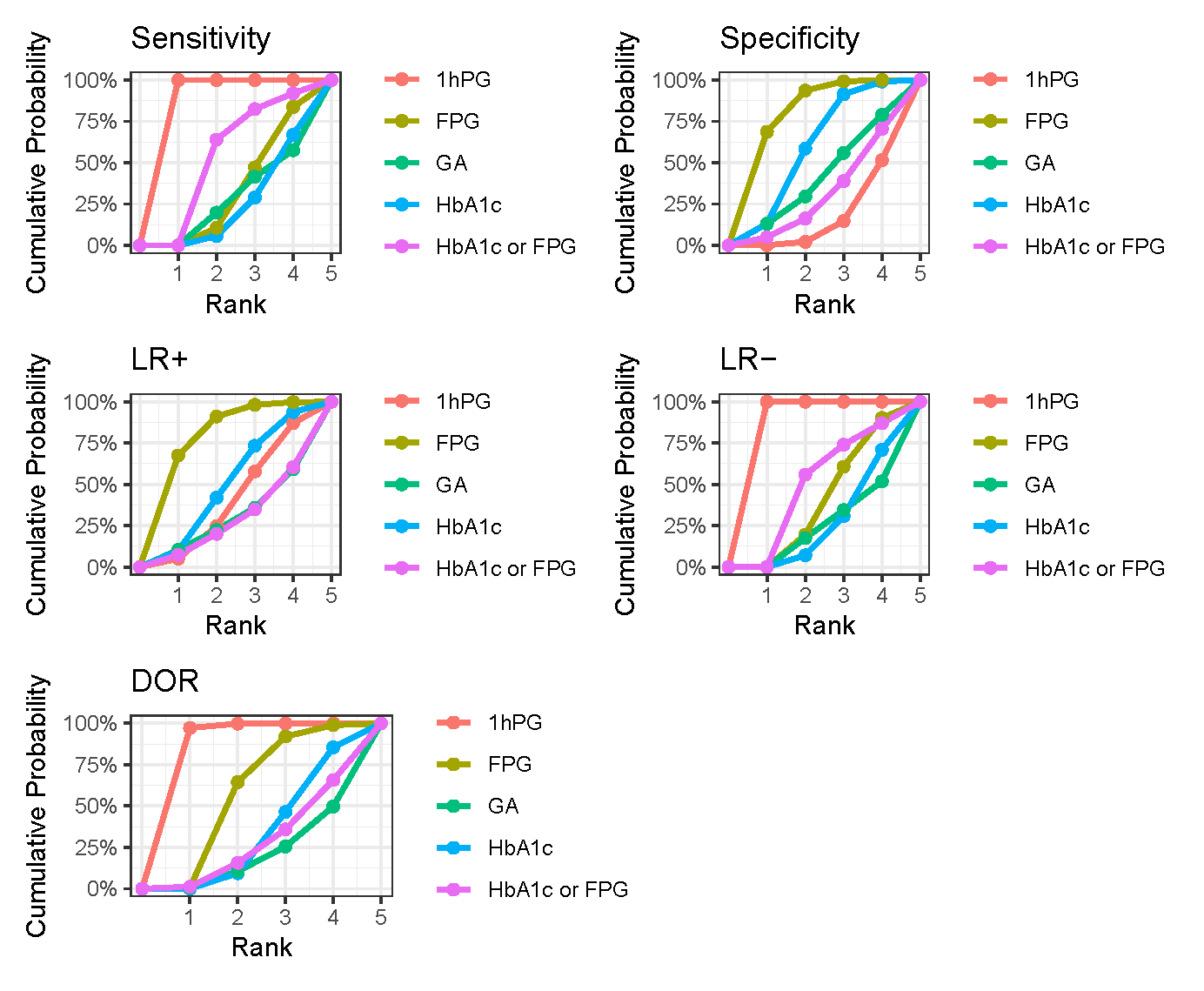
 **Supplementary Figure 18.** Cumulative ranking curves for the five diagnostic strategies in sensitivity analysis. Ranking probabilities are shown for sensitivity, specificity, positive likelihood ratio (LR+), negative likelihood ratio (LR−), and diagnostic odds ratio (DOR).

1-h PG, 1-hour plasma glucose; HbA1c, glycated hemoglobin; FPG, fasting plasma glucose; GA, glycated albumin.

**Supplementary Tables 6A–E.** League tables of relative diagnostic performance in sensitivity analysis. Relative comparisons among 1-h PG, HbA1c, FPG, GA, and combined HbA1c or FPG are presented for sensitivity (A), specificity (B), positive likelihood ratio (LR+) (C), negative likelihood ratio (LR−) (D), and diagnostic odds ratio (DOR) (E), with posterior estimates and 95% credible intervals.

1-h PG, 1-hour plasma glucose; HbA1c, glycated hemoglobin; FPG, fasting plasma glucose; GA, glycated albumin.
